# Protein Thioether Crosslinks Installed by a SPASM-Containing Radical SAM Enzyme from Methanogenic Archaea

**DOI:** 10.1021/acsbiomedchemau.6c00133

**Published:** 2026-09-21

**Authors:** Emily M. Dieter, Austin M. Woodard, Jared C. Green, Jin Xiong, Yisong Guo, William E. Broderick, Douglas A. Mitchell, Joan B. Broderick

**Affiliations:** † Department of Chemistry and Biochemistry, 33052 Montana State University, Bozeman, Montana 59717, United States; ‡ Department of Chemistry and Biochemistry, Gettysburg College, Gettysburg, Pennsylvania 17325, United States; § Department of Chemistry, University of Illinois at Urbana-Champaign, Urbana, Illinois 61801, United States; ∥ Department of Biochemistry, 5718 Vanderbilt University School of Medicine, Nashville, Tennessee 37232, United States; ⊥ Department of Chemistry, 6612 Carnegie Mellon University, Pittsburgh, Pennsylvania 15213, United States; # Department of Chemistry, Texas A&M University, College Station, Texas 77843, United States; ∇ Department of Chemistry, Vanderbilt University, Nashville, Tennessee 37232, United States

**Keywords:** RiPPs, metalloenzymes, bioinformatics, Methanococcus voltae, cysteine-rich protein, mass spectrometry, EPR

## Abstract

Cysteine is a vital amino acid with wide biological utility, serving functions in catalysis, ligand binding, and maintenance of protein structures. In a subset of natural products, termed ribosomally synthesized and post-translationally modified peptides (RiPPs), multiple subclasses are defined by the enzymatic modification of cysteine residues to form thioether bridges. However, RiPPs are short peptides and experimental validation of enzymatically installed thioether crosslinks on large protein substrates is limited. We hypothesized that cysteine-rich proteins whose genes are adjacent to those for enzymes predicted to catalyze RiPP-like modifications might be substrates for such modifications. Thus, we set out to investigate cysteine-rich prokaryotic proteomes for such examples. A bioinformatic analysis identified methanogenic archaea as suitable candidates for further investigation due to the high overall proteome cysteine content, and through targeted proteome scanning, we identified a suitable biosynthetic gene cluster (BGC; Crp) from *Methanococcus voltae* to characterize. Purification and reconstitution of an annotated radical *S*-adenosyl-l-methionine (RS) enzyme, CrpC, confirmed that the protein binds three [4Fe-4S] clusters, two of which are in a SPASM domain. Enzyme assays determined that CrpC is active after reconstitution and modifies a cysteine-rich protein substrate in the same BGC, CrpA. Mass spectrometry and chemical derivatization of modified CrpA indicates that CrpC installs ranthionine crosslinks onto CrpA. This research provides a new path for the discovery of intriguing BGCs and post-translational modifications and provides a foundation for the complete structural elucidation and assessment of the native biological function of these modified cysteine-rich proteins.

## Graphical abstract

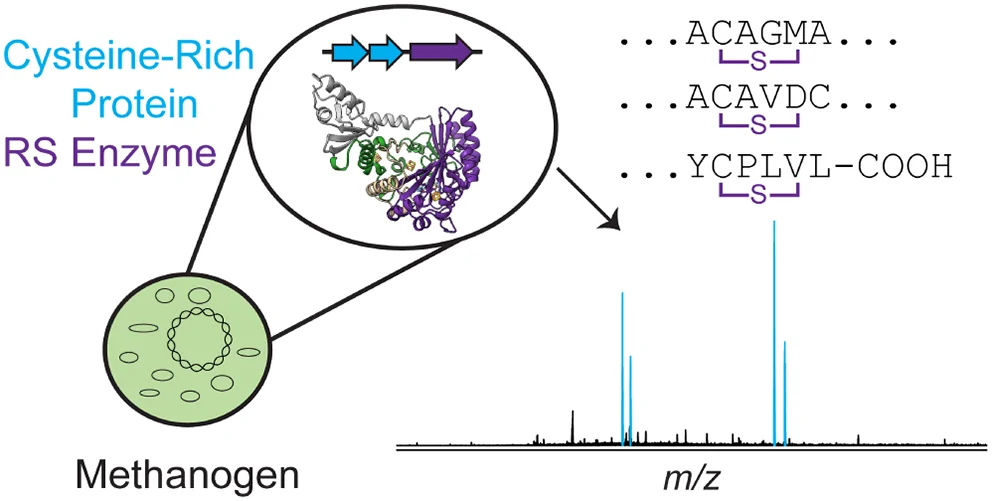

## Introduction

Iron and sulfur are essential elements utilized across all domains of life. As an enzyme cofactor, iron is found in [Fe–S] clusters, heme, and other nonheme iron proteins.^1–3^ In cells, sulfur is prominently found in amino acids such as cysteine, one of the least abundant but most functionally important amino acids. The cysteine thiol has a range of possible sulfur oxidation states, enabling multiple functions in peptides and proteins.^4,5^ Normal and modified cysteine residues help maintain protein structure and modulate function, regulate oxidative stress, and perform nucleophilic catalysis, among other functions.^4–6^

Additionally, cysteine is often utilized to ligate essential cofactors in metalloproteins, including [Fe–S] clusters.^5,7^ Proteins that ligate [Fe–S] clusters have diversified redox functions ranging from electron transfer in ferredoxins, to radical initiation in radical *S*-adenosyl-l-methionine (SAM) enzymes.^1,7^ Radical SAM (RS) enzymes comprise the largest known enzyme superfamily and are responsible for catalyzing reactions in both primary and secondary metabolism across all domains of life.^7–12^ Three cysteines in a conserved active site motif, most commonly in the form of CX_3_CX_2_C, coordinate three iron atoms in a site-differentiated [4Fe-4S] cluster, while the fourth iron is coordinated by SAM.^7,13–16^ RS catalysis is initiated when the reduced [4Fe-4S]^+^ cluster transfers an electron to SAM, promoting S–C5′ bond homolysis.^17^ Upon homolytic cleavage of SAM, an adenosyl-[4Fe-4S] organometallic intermediate is formed, followed by a highly reactive 5′-deoxyadenosyl radical (5′-dAdo•); the 5′-dAdo• generates a substrate radical by H atom abstraction, producing 5′-deoxyadenosine (5′-dAdoH), and initiating the diversity of RS enzyme-catalyzed reactions.^9,18–25^ A subset of RS enzymes ligate additional [4Fe-4S] clusters in a SPASM domain (named for the founding members of this subclass which are involved in subtilosin A, pyrroloquinoline quinone, anaerobic sulfatase, and mycofactocin maturation), or the related but smaller twitch domain.^26–29^ Canonical SPASM domains contain an eight-cysteine motif, and the auxiliary clusters have been proposed to aid in substrate binding and electron transfer.^30–33^

Functionally, a portion of SPASM/twitch domain-containing RS enzymes have been shown to modify ribosomally synthesized and post-translationally modified peptides (RiPPs).^28,34^ RiPP precursors are typically short peptides genomically encoded adjacent to modifying enzymes, transporters, and other relevant proteins in biosynthetic gene clusters (BGCs).^34,35^ These precursor peptides are composed of two portions: the N-terminal leader region that is normally implicated in enzyme-substrate recognition, and the C-terminal core region that is enzymatically modified. Oftentimes, RiPP biosynthetic enzymes utilize the RiPP recognition element (RRE) for substrate-leader peptide engagement.^36,37^ The RRE can be found fused to modifying enzymes, or as a discrete protein. Following modification, the peptides may undergo proteolysis to liberate the final natural product.^34,35,38,39^ RiPP-RS modifications include C–C, C–O, and C–S crosslink formation, methylation, epimerization, and more.^34,40–42^

Several RiPP classes contain peptide substrates enriched in cysteine. Lanthipeptides, sactipeptides, and ranthipeptides are defined by modification of cysteine residues to form thioether crosslinks (Figure S1).^34,35^ In lanthipeptides, β-thioether crosslinks are generated via the addition of a cysteine thiol to a dehydrated serine or threonine.^34,43,44^ Alternatively, sactipeptides and ranthipeptides are modified by RS enzymes to form either a sulfur to α-carbon thioether (sactionine) or a sulfur to non-α-carbon thioether (ranthionine) crosslink, respectively.^34,35,43,45,46^ Ranthionine linkages have also been observed in proteins with RiPP-like features. For example, the RS enzyme QhpD (NCBI WP_011747999.1) installs three thioether crosslinks (one Cys-Glu (S–Cγ) and two Cys-Asp (S–Cβ) crosslinks) into the small subunit (QhpC, NCBI WP_011748000.1) of quinohemoprotein amine dehydrogenase (QHNDH) (Figure S1).^47–49^ While not a RiPP-RS, QhpD shows high sequence homology to ranthisynthases like PapB and CteB and utilizes an RRE to engage the leader peptide of QhpC.^46,49,50^

In most prokaryotes, cysteine ribosomal incorporation follows tRNA^Cys^ aminoacylation with free cysteine.^51^ However, a subset of archaea use an ancestral pathway where tRNA^Cys^ is aminoacylated with *O*-phosphoserine (Sep), then Sep-tRNA^Cys^ is converted directly to Cys-tRNA^Cys^.^52^ This alternative cysteine pathway is mainly utilized by methanogenic archaea (“methanogens”), organisms that have been demonstrated to have higher than normal proteomic cysteine content compared to other archaea.^53,54^ Methanogens, anaerobic organisms that generate methane, rely heavily on [Fe–S] cluster-containing proteins to meet metabolic demands and alleviate environmental stress.^54–58^ However, the high number of [Fe–S] clusters does not fully account for the significant increase in proteome cysteine content, suggesting the presence of cysteine-rich proteins with diverse functions. Consequently, we set out to examine archaeal proteomes for cysteine-rich proteins. In this study, we developed a domain-wide proteome-level bioinformatic workflow to prioritize the discovery of proteins and BGCs with desired features (high cysteine content). Using this method, we identified a BGC from *Methanococcus voltae* with a cysteine-rich protein (CrpA) that is modified by an RS enzyme (CrpC). We characterized CrpC via site-directed mutagenesis, elemental analysis, Mössbauer spectroscopy, and electron paramagnetic resonance (EPR) spectroscopy to probe the iron–sulfur cluster content and properties, establishing that the enzyme binds three [4Fe-4S] clusters, one in the active site and two more in a SPASM domain. Extensive chemical derivatization and mass spectrometry experiments suggest that CrpC catalyzes the formation of ranthionine crosslinks onto CrpA, providing an unusual example of a RiPP-like modification on a large protein.

## Results

### Analysis of Prokaryotic Proteomes Identifies Methanogens with High Cysteine Content

Due to thiol reactivity, cysteine is often confined to functionally or structurally relevant locations in proteins, leading to the generally observed low cysteine content of proteins.^5,6,59^ Thus, when cysteine is present in high abundance in a protein, it suggests a unique role. We hypothesized that prokaryotic proteomes that are generally enriched in cysteine would provide examples of cysteine-rich proteins which could be probed for novel functions. Analysis of NCBI reference proteomes^60^ for archaea (*n* = 765) and bacteria (*n* = 21,439) demonstrates that the average proteome cysteine content is similar at 0.87% for bacteria and 0.85% for archaea (Figure 1A-B). However, archaeal proteomes show a bimodal distribution of cysteine content that is more prominent when analyzing all RefSeq annotated archaeal proteomes (*n* = 2,660), not just reference proteomes (Figure 1C). The low and high cysteine archaea have an average cysteine content of 0.69% and 1.29%, respectively, consistent with previous observations in a smaller data set.^54^ Interestingly, ∼70% of the high cysteine archaea were assigned as methanogens (*n* = 757/1,084 proteomes) based on the presence of proteins composing key methanogenesis protein complexes, Methyl-coenzyme M reductase (MCR) and N^5^-methyltetrahydromethanopterin:coenzyme M methyltransferase (MTR), while <0.2% of the low cysteine archaea were assigned as methanogens (*n* = 2/1,576 proteomes; *p* ≪ 0.0001) (Table S1).^61,62^ Experimentally validated methanogens, *Methanococcus maripaludis* and *Methanosarcina acetivorans*, were present in the high-cysteine proteome data set.^63,64^

**Figure 1:**
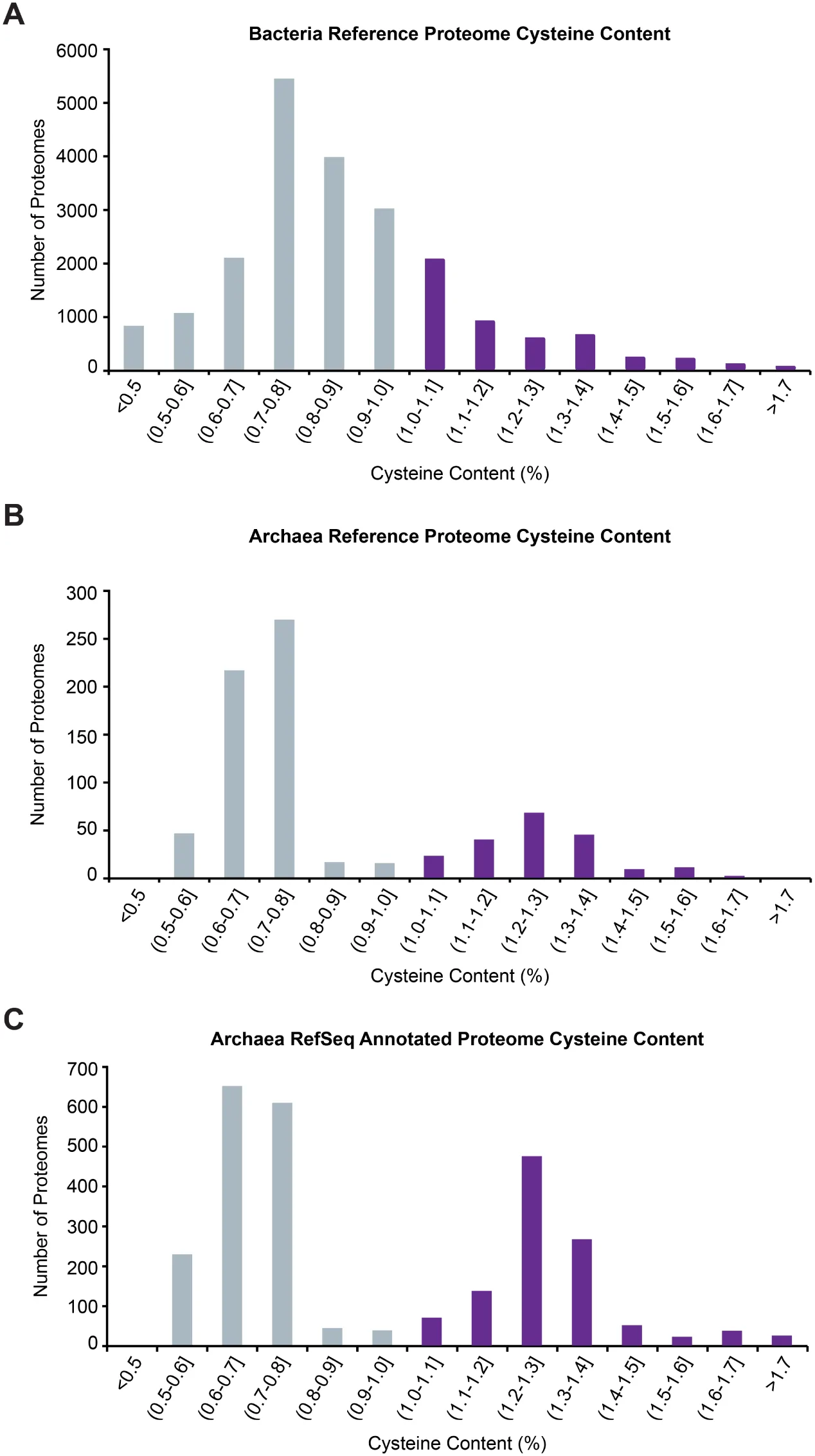
Proteome-wide cysteine content distribution in bacteria and archaea. Histograms showing the proteome cysteine content distribution for (A) bacterial NCBI reference proteomes (*n* = 21,439; mean = 0.87% cysteine), (B) archaeal NCBI reference proteomes (*n* = 765; mean = 0.85% cysteine), and (C) all archaeal NCBI RefSeq-annotated proteomes (*n* = 2,660; mean = 0.93% cysteine). High-cysteine proteomes (>1%) are colored purple.

### Bioinformatic Identification of Archaeal Cysteine-Rich Proteins Adjacent to RS Enzymes

These high-cysteine archaea (1,084 proteomes with a >1% cysteine content) were selected for further investigation to identify cysteine-rich proteins. The designated proteomes were scanned for proteins with a cysteine content ≥10% (Figure S2). We limited our investigation to larger proteins (≥100 AA) as smaller peptides can have an artificially high cysteine content due to their size. Additionally, to account for cysteine-rich domains in larger proteins that have a low overall cysteine content, we included proteins with a cysteine content ≥5% if the protein was larger than 250 AA.

Our analysis identified 6,743 unique cysteine-rich proteins, 94.3% of which were retrieved by a published Hidden Markov Model (HMM; Pfam^65^ or TIGRFAM^66,67^ ), with the remaining 5.7% unannotated. Many of the cysteine-rich proteins are predicted to ligate metallic cofactors (Table S2). The genomic context for these cysteine-rich proteins was also investigated. Although the most frequent neighboring proteins are predicted to be involved in electron shuttling and cofactor regeneration, RS enzymes are also present adjacent to cysteine-rich proteins (Table S3). RS enzymes have been demonstrated to modify cysteine-rich RiPPs; thus, we hypothesized that they would modify some of the identified cysteine-rich proteins.^34,35,43,45,46^ Furthermore, RiPP pathways in archaea have started to be more thoroughly investigated and present an intriguing area of current research.^68–70^ Unannotated cysteine-rich proteins were filtered to retain only those in which a non-nucleic acid-modifying RS enzyme was adjacent, resulting in 65 cysteine-rich proteins of interest associated with 41 unique RS enzymes. When analyzed in a sequence similarity network (SSN), the associated RS enzymes cluster in 4 groups and 3 singletons (Figure 2A). RS enzymes in groups 1, 2, and 4 are highly conserved in predicted functions based on HMM retrieval, while RS enzymes in group 3 are diverse (Table S4). Cysteine-rich proteins associated with RS enzymes in group 3 contain repeating cysteine motifs implying a functional role (Figures 2B, S3). Of those, we identified a BGC from *Methanococcus voltae* A3 (*M. voltae*) for further investigation (Figure 2B). Interestingly, all three of the proteins in this BGC had been shown to be upregulated when *M. voltae* was grown on pyrite (FeS_2_).^71–73^ This indicates a possible role for these proteins in the reductive dissolution of iron and sulfur to meet biosynthetic needs.

**Figure 2:**
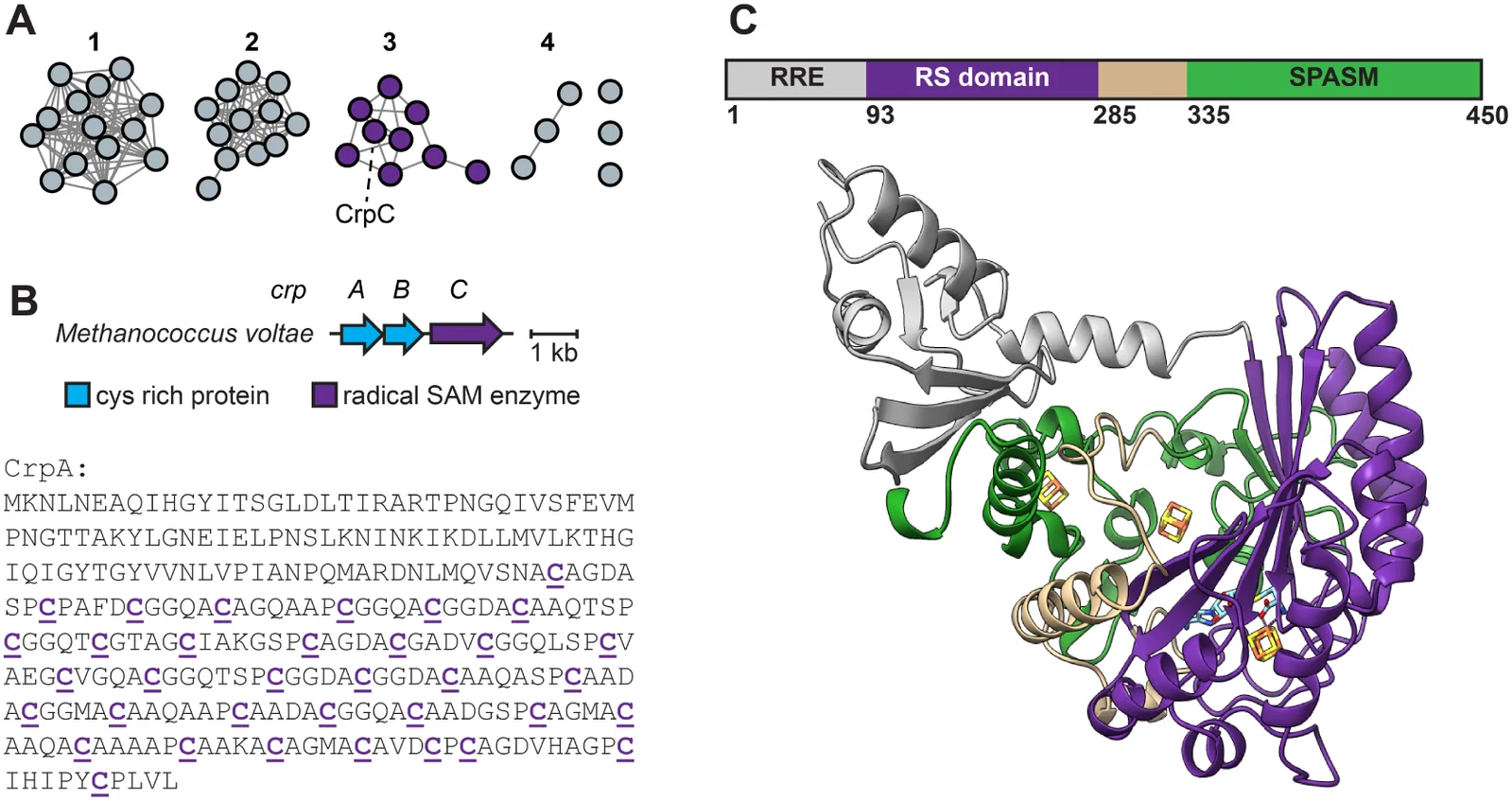
Bioinformatic discovery of archaeal cysteine-rich proteins. (A) Sequence similarity network (SSN) of RS enzymes associated with cysteine-rich proteins at an alignment score of 8. Each node represents a single protein sequence. RS enzymes of interest are colored purple (see Figure S3 for further analysis), and CrpC is indicated. (B) Crp BGC diagram. The sequence of CrpA is shown with C-terminal cysteines colored purple and underlined. (C) Domain architecture diagram and AlphaFold3 ^74^ structure of CrpC. The predicted RRE, RS, and SPASM domains are shown in gray, purple, and green, respectively. Bridging regions are colored tan. SAM and [4Fe-4S] clusters were inserted from alignment with the crystal structure of CteB (PDB: 5WHY).^32^

The immediate Crp BGC contains three proteins, CrpA (cysteine-rich protein; NCBI WP_013180081.1), CrpB (NCBI WP_013180082.1), and CrpC (RS enzyme; NCBI WP_013180083.1). CrpC belongs to an orphan RS family (TIGR04463 ^66,67^ ) whose members are predicted to modify large, cysteine-rich proteins encoded proximally in the genome. CrpA and CrpB share almost an identical first 47 N-terminal residues but diverge after this region (Figure S4). The first 47 residues and the C-terminal localization of cysteine residues are similar to the leader peptide and core region in RiPPs, respectively.^34,35^ In CrpA, there is a 17-residue cysteine motif that is repeated 10 times, whereas in CrpB, a 34-residue motif is repeated twice (Figure S4). Both C-terminal repetitive regions contain a C-X-X-Q/D motif, suggestive of RS-catalyzed thioether formation.^46,75^ However, as CrpB did not meet our threshold (4.7% cysteine content), we decided to focus only on CrpA as a potential precursor due to the much higher abundance of cysteine residues. AlphaFold3 structural modeling predicts that the C-terminal cysteine-rich region of CrpA forms a β-helix, reminiscent of pectin lyase (PDB: 1IDJ); however, this C-terminal region has high predicted alignment error (PAE) and low local confidence (predicted local distance difference test; pLDDT) (Figure S5).^74,76^ CrpA homology searches via BLAST-P^77^ identified additional RS-associated single- and multi-domain cysteine-rich proteins found in bacteria, archaea, and eukaryota suggesting a diversity of roles (Supplemental Data set 1).

### CrpC Is an RS Enzyme That Contains a Predicted SPASM Domain and RRE

CrpC domain architecture and [Fe–S]-binding cysteine residues were investigated with RadicalSAM.org, a web-based resource with SSNs and genome neighborhood diagrams for the entire RS enzyme superfamily.^78^ CrpC is located in Megacluster-1–1, the SPASM/twitch domain-containing subgroup. Cys104, Cys108, and Cys111 of CrpC are predicted to coordinate the SAM-binding [4Fe-4S] cluster, while Auxiliary cluster I (AuxI) is coordinated by Cys333, Cys352, and Cys403, and finally, AuxII is coordinated by Cys390, Cys393, Cys399, and Cys422.^29,74^ Residues 93–271 are predicted to compose the RS domain, while residues 335–450 make up the SPASM domain (Figure 2C). The remaining unannotated residues, 1–92, were assigned as a potential RRE based on HHpred and AlphaFold3 modeling.^74,79^ Using these insights, we hypothesized that CrpC is an RS enzyme containing both a SPASM domain and an RRE.

### CrpC Contains Three [4Fe-4S] Clusters

We next set out to experimentally validate the number and configuration of predicted [Fe–S] clusters. Wild-type (WT) CrpC was successfully expressed in *Escherichia coli* (*E. coli*) as an N-terminal His_10_-tag fusion, then purified anaerobically (Figure S6). Furthermore, cysteine residues that were predicted to be involved in iron–sulfur cluster coordination were substituted with alanine, resulting in three cluster variants: ΔRS (C104A, C108A, and C111A), ΔAuxI (C333A, C352A, and C403A), and ΔAuxII (C390A, C393A, C399A, and C422A) (Figure S7). All purified cluster variants were soluble, free of major contaminants, and predominantly found in either the monomeric or dimeric state, with peaks at retention times corresponding to ∼50 kDa and ∼100 kDa (expected monomeric mass: 54.2 kDa) (Figures S6, S8). Following purification, the CrpC WT and cluster variants were chemically reconstituted with ferrous ammonium sulfate (FAS), lithium sulfide (Li_2_S), and dithiothreitol (DTT) (Figure 3A). Reconstituted protein exhibited a broad spectral feature at 420 nm, characteristic of [4Fe-4S] clusters, while as purified protein lacked this 420 nm feature indicating successful cluster incorporation upon reconstitution (Figure 3B). The reconstituted enzymes were subjected to atomic absorption analysis and Beinert’s assay to quantify iron and sulfur content, respectively (Table S5).^80^ The WT enzyme contained ∼12 Fe and S equivalents per monomer and single cluster alanine variants contained ∼8 Fe and S equivalents per monomer (Table S5).

**Figure 3:**
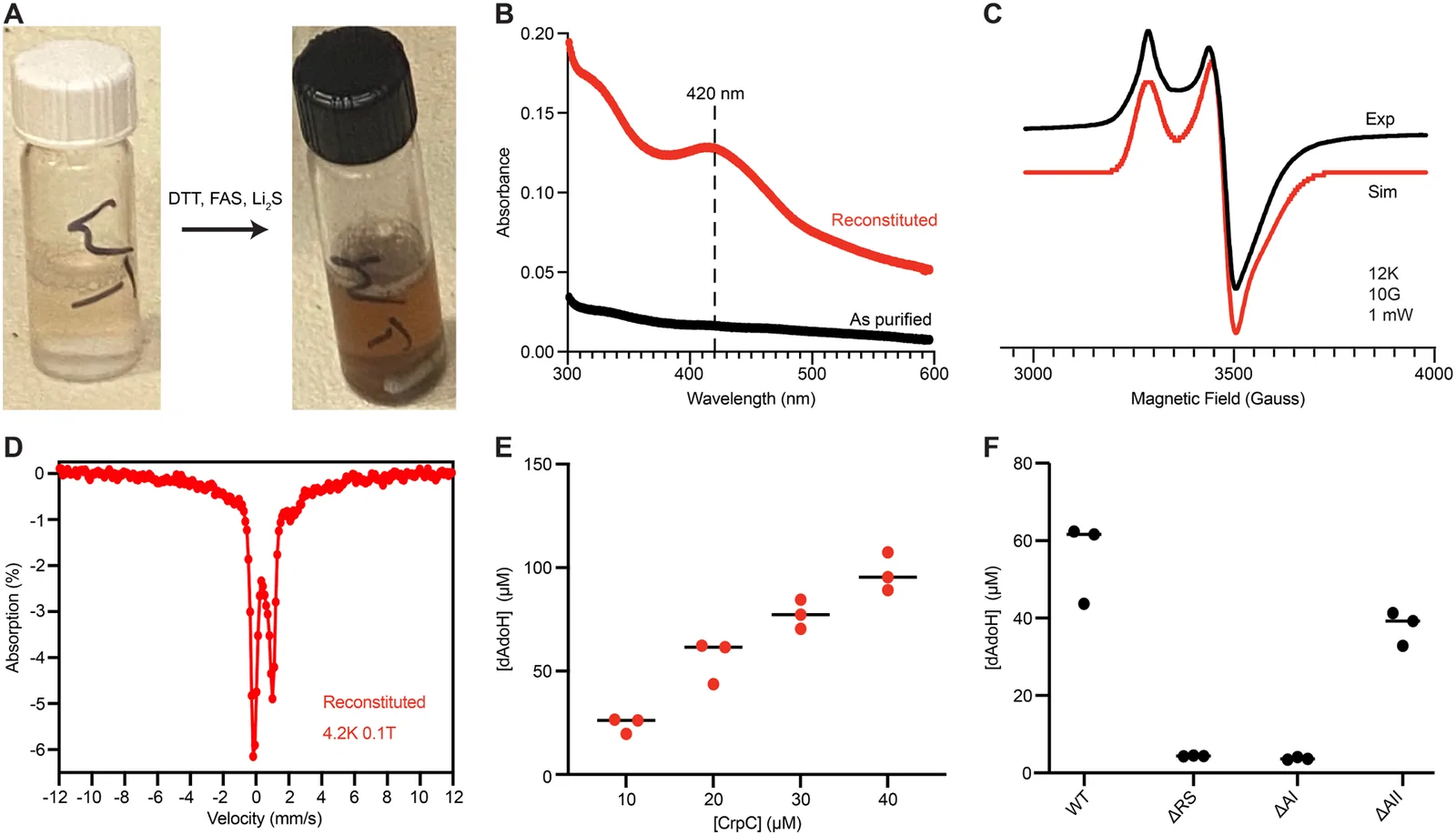
Spectroscopic and functional characterization of CrpC. (A) Images of purified WT CrpC *left:* before and *right:* after reconstitution. (B) UV–Vis spectra of WT CrpC before (black) and after (red) reconstitution. (C) Continuous wave X-band EPR spectrum of reconstituted WT CrpC following reduction with sodium dithionite (NaDT) (black) and the simulated spectrum (red). The spectrum was collected at 12 K with a microwave frequency of 9.375 GHz and a modulation amplitude of 10 G. Simulation was generated with EasySpin.^81^ (D) Mössbauer spectrum of CrpC after reconstitution. The spectrum reveals an isomer shift of δ = 0.43 mm/s and quadrupole splitting of Δ*E*_q_ = 1.08 mm/s consistent with [4Fe-4S]^2+^ clusters. (E) Enzymatic activity of reconstituted CrpC as assessed by measuring 5′-dAdoH production after a 3 h incubation with dithionite. (F) Enzymatic activity assays for different CrpC [4Fe-4S] cluster alanine variants. Assays included CrpC, DTT, NaDT, and SAM and were carried out for 3 h, after which the production of 5′-dAdoH was quantified following HPLC.

Electron paramagnetic resonance spectroscopy was used to probe the composition of the [Fe–S] clusters in CrpC. Reconstituted WT CrpC was reduced with 10 equiv of sodium dithionite (NaDT) and then analyzed using EPR spectroscopy (Figures 3C, S9). The protein was EPR active and exhibited an axial signal with *g* values of 2.04 and 1.94, which are typical of [4Fe-4S]^+^ clusters. Mössbauer spectroscopy was carried out on the reconstituted WT protein, and the 4.2 K/0.1 T spectrum shows a single quadrupole doublet, indicating that most of the iron is found within [4Fe-4S] clusters (Figure 3D). Together with the iron and sulfur contents determined for the WT and the alanine cluster variants, the spectroscopic characterization of CrpC is consistent with the presence of three [4Fe-4S] clusters in the WT enzyme.

### All Three [4Fe-4S] Clusters Are Necessary for Maximal SAM Reductive Cleavage Activity

CrpC was assessed *in vitro* for the ability to reductively cleave SAM, an enzymatic activity that is shared by all RS enzymes.^7,21,24^ Reactions that contained CrpC (either WT or alanine cluster variants), SAM, NaDT, and DTT were allowed to react for 3 h, then quenched with acetic acid. Reactions were analyzed using HPLC, and the absorbance at 254 nm was monitored to quantify 5′-dAdoH production. We observed that 5′-dAdoH was produced in a concentration-dependent manner by WT CrpC (Figure 3E). When the RS cluster was absent, minimal 5′-dAdoH production was observed, illustrating the necessity of the RS [4Fe-4S] cluster for the reductive cleavage of SAM (Figure 3F). If AuxII was removed, SAM reductive cleavage activity was still observed, albeit to a lesser extent than the WT protein. However, the AuxI cluster variant had minimal SAM reductive cleavage activity, potentially due to its postulated role in regenerating the RS cluster for catalysis via electron transfer.^30,31,33^ These results are consistent with those previously reported with other RS enzymes containing a SPASM domain, indicating that the RS cluster remains intact when the auxiliary clusters are removed.^82^ This ability to produce 5′-dAdoH, combined with the spectroscopic and computational analyses of CrpC, conclusively demonstrates that CrpC is a RS enzyme.

### Installation of Thioether Crosslinks in CrpA

It has previously been shown that RS enzymes can cleave SAM and produce 5′-dAdoH in the absence of a substrate, but the amount of 5′-dAdoH usually increases in the presence of the preferred substrate.^7,83^ As an initial test of the hypothesis that CrpA is a substrate of CrpC, CrpA with an N-terminal fusion to maltose-binding protein (MBP) containing a His_6_-tag was expressed in *E. coli*, then anaerobically purified using Ni-NTA affinity chromatography (Figure S6). Upon treating purified CrpA with CrpC and SAM in the presence of dithionite, we observed increased 5′-dAdoH production compared to assays carried out in the absence of CrpA, supporting CrpA as a substrate of CrpC (Figure 4A).

**Figure 4:**
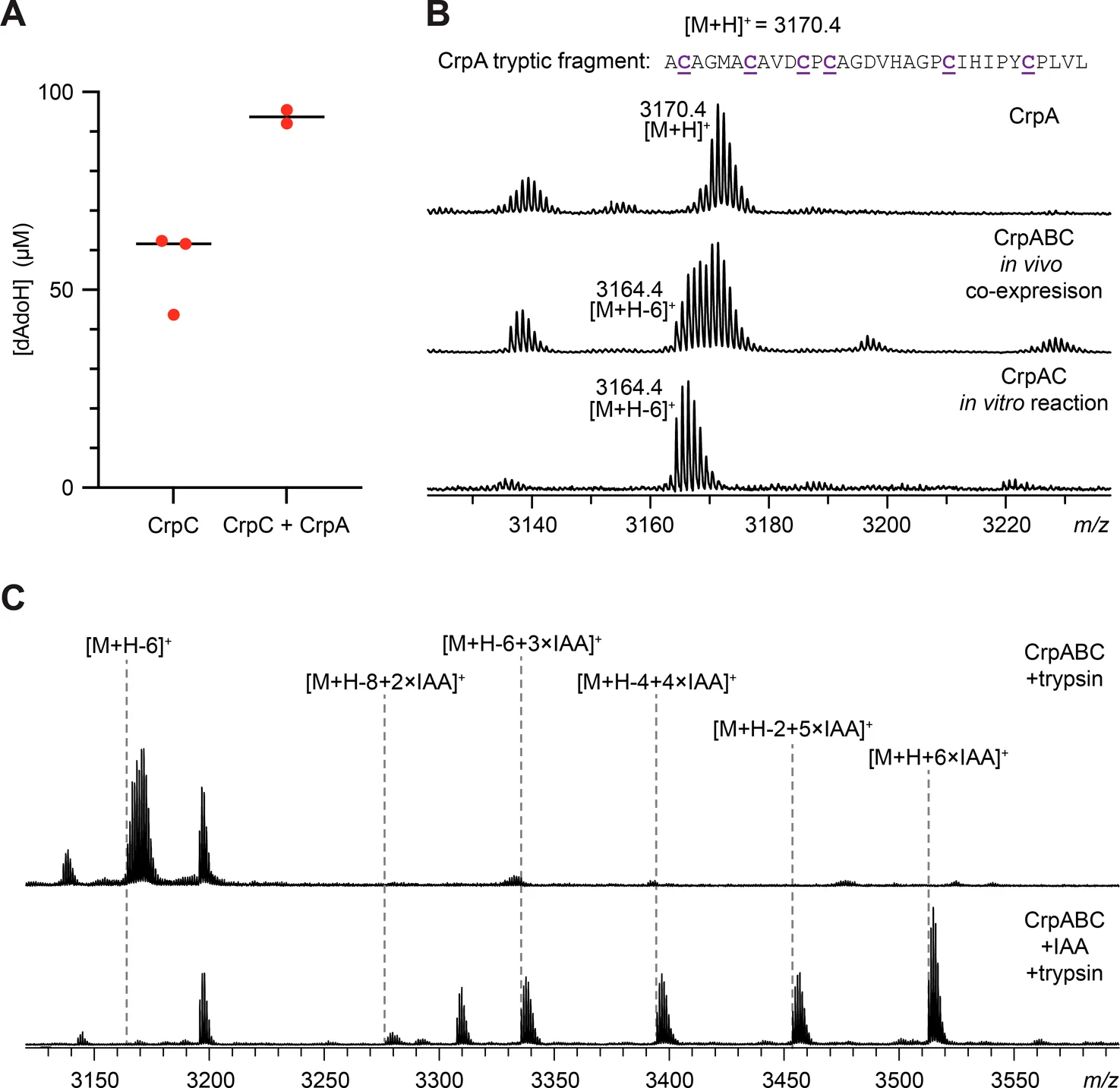
Analysis of modified CrpA. (A) 5′-dAdoH produced following the reaction of CrpC with SAM and dithionite in the absence and presence of CrpA. See Materials and Methods for further experimental details. (B) MALDI-TOF mass spectra of the C-terminal CrpA tryptic peptide from different conditions. The sequence for the tryptic fragment of interest is provided with the unmodified mass given. Spectra shown are from CrpA purified from *E. coli* expressing only CrpA (*top*), CrpA purified from *E. coli* expressing CrpA, CrpB, and CrpC (*middle*), or CrpA following the *in vitro* activity assay (*bottom*). (C) Iodoacetamide (IAA; +57 Da) labeling reaction with trypsin-digested and modified CrpA. The same C-terminal fragment as in (B) was analyzed. HR-MS/MS spectra were collected for observed species (Figures S11–S20 and Tables S6–S14).

We next characterized the modifications installed by CrpC onto CrpA. To generate modified products, two approaches were pursued. For the *in vitro* approach, activity assays containing CrpC, CrpA, SAM, and dithionite were run as previously described. For the *in vivo* approach, a plasmid encoding His_10_-CrpC and His_6_-MBP-CrpA was cotransformed with a plasmid encoding CrpB into *E. coli*. Following expression, CrpA was selectively purified through a two-step affinity purification utilizing both the His_6_ and MBP tag. The tags were removed using tobacco etch virus (TEV) protease. Products from the *in vitro* activity assay and purified CrpA from the coexpression were then treated with trypsin and analyzed by matrix-assisted laser desorption/ionization-time-of-flight-mass spectrometry (MALDI-TOF-MS). Tris­(2-carboxyethyl)­phosphine hydrochloride (TCEP) was included in all samples to prevent disulfide formation. Trypsinolysis of CrpA would produce four separate cysteine-rich peptide fragments ranging from 3000 to 9000 Da; however, the size and hydrophobicity of the larger fragments limited detection via MALDI-TOF-MS. Only the C-terminal fragment, which contained six cysteines, was observed with high resolution. Negative ion mode acquisition was attempted as well with the abundance of Asp and Glu residues, but it did not improve observation of the larger cysteine-rich fragments. Additional proteases were tested, but coverage of the cysteine-rich region was not substantially improved. The C-terminal CrpA tryptic fragment (CrpA_CTryp_) analyzed from both the *in vitro* and *in vivo* approaches had a mass of 3164.4 Da, corresponding to a loss of 6 Da from the unmodified peptide fragment (Figure 4B). Furthermore, the isotopic ratio for the *in vivo* purified sample was broader than expected for a peptide at this mass range, suggestive of multiple species with different numbers of modifications (Figure S10).

To further understand the different extents of modification, modified CrpA from the in vivo experiments was then treated with thiol-reactive iodoacetamide (IAA), followed by trypsinolysis (CrpA_CITryp_). After reaction with IAA, five new species at 3276.4 Da (2×IAA, −8 Da), 3335.4 Da (3×IAA, −6 Da), 3394.4 Da (4×IAA, −4 Da), 3453.5 Da (5×IAA, −2 Da), and 3512.5 Da (6×IAA, −0 Da) were detected (Figure 4C). The number of modifications (each matching the loss of 2H) is inversely correlated with the number of IAA alkylations, consistent with cysteine thiol modification. The IAA-labeled sample was then analyzed by LC-MS/MS with higher-energy collisional dissociation (HCD) fragmentation. Both the 5×IAA and 4×IAA CrpA_CITryp_ peptides exist as a mixture of isomers as each peptide eluted at three separate retention times. In contrast, the 6×IAA, 3×IAA, and 2×IAA CrpA_CITryp_ peptides exist as single isomers as each eluted at a single retention time (Figures S12–S20 and Tables S6–S14). Tandem MS analysis of all CrpA_CITryp_ peptides localized the −2 Da modifications to four different motifs, each with an unalkylated cysteine (ACAGM, CAVD, PCAGD, and CPLVL) (Figure 5A). All four motifs were modified in the 2×IAA peptide, while the ACAGM, CAVD, and CPLVL motifs were modified in the 3×IAA peptide. The 4×IAA and 5×IAA peptides had different combinations of the ACAGM, CAVD, and CPLVL motifs modified in their respective isomers. Notably, no fragmentation was observed within any modified motifs (Figures S12–S20 and Tables S6–S14).

**Figure 5:**
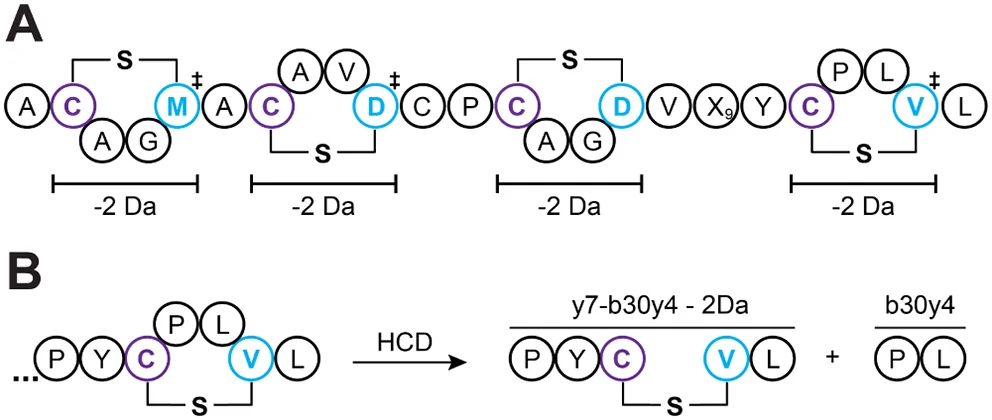
Thioether crosslink formation on CrpA. (A) CrpA_CTryp_ shown in bubble diagram with modified cysteine residues colored purple and acceptor residues colored blue. Observed mass losses are indicated based on MS/MS. ‡ indicates acceptor residues with an additional deuterium after desulfurization. (B) Formation of observed fragmentation pattern suggestive of a ranthionine crosslink. See Figures S12–S24 and Tables S6–S16 for full MS/MS and exact masses.

HCD-based MS/MS produced useful diagnostic daughter ions. For example, we consistently observed a prominent *m/z* value of 211.14 in all MS/MS spectra, which can be attributed to the b30y4 internal fragment (PL) from within the C-terminal cysteine motif (CPLVL). The b30y4 internal fragment is still observed even when the CPLVL motif is modified. The complementary y7-b30y4 (PYC-VL −2 Da) daughter ion can be observed, albeit at low abundance, indicating that the thioether crosslink is retained after internal fragmentation (Figure 5B). Sactionine crosslinks are known to dissociate in CID whereas ranthionine crosslinks do not.^46^ Furthermore, internal fragmentation within a thioether macrocycle has precedence in lanthipeptides which have thioether crosslinks reminiscent of RS-installed ranthionine crosslinks.^46,84,85^ Combined, these results suggest that ranthionine thioether crosslinks were installed by CrpC. A previous report demonstrated that the identity of six RS active-site residues, termed the catalytic proximity residues, can differentiate sactisynthases from ranthisynthases.^50^ Catalytic proximity profiling indicates that CrpC is more closely related to ranthisynthases (e.g., PapB; NCBI WP_019688962.1) than sactisynthases (e.g., AlbA; NCBI WP_003242599.1) and other non-thioether forming RS enzymes, aligning with our experimental results (Figure S25).

With evidence suggestive of thioether crosslink formation, we performed reductive desulfurization under deuterated conditions, a reaction that will incorporate deuterium into cysteine, methionine, and thioether acceptor residues.^50,86^ Desulfurization of cysteine and methionine residues with concurrent deuterium replacement was successful as determined by LC-MS/MS (Figure S21). However, the observed isotopic ratio was broader than expected, indicating that a mixture of products was produced as observed with *in vivo*-modified CrpA (Figure 4B). These different products were identified as desulfurized peptide fragments with incorporation of up to 4 additional deuteriums. Each additional deuterium incorporated correlates with a labeled acceptor residue involved in a thioether modification. Overlapping ^13^C isotopes and precursor ions complicated MS/MS assignment for the 3× modified peptide, but comparison of daughter ion isotopic distributions and mass differences (^13^C–^12^C: 1.0033 Da vs ^2^H–^1^H: 1.0062 Da) localized additional deuterium incorporation to previously observed modified motifs (ACAGM, CAVD, and CPLVL) (Figures 5, S21–S24 and Tables S15–S17). These results agree with our other data demonstrating RS-installed thioether crosslinks. Key internal fragments were able to narrow down the sites of modification for the ACAGM and CPLVL motifs to four residues (CAGM and CPLV).

### CrpC Substrate Engagement Is Facilitated by N-Terminal Protein Domains

As CrpC is predicted to harbor an RRE domain, we set out to interrogate leader binding. AlphaFold3 multimer prediction of CrpAC shows a canonical RRE-leader interaction; however, unlike most other RiPP precursor peptides, CrpA has an additional N-terminal domain that appears to also contribute to binding (Figure S26).^36^ CrpA leader truncations were generated with the removal of up to the full N-terminal domain. Separately, the RRE domain was removed from CrpC. After *in vivo* coexpression of CrpAC, MBP-CrpA was purified, alkylated, proteolyzed, and analyzed by MALDI-TOF-MS to determine the extent of processing (Figure S27). CrpB was omitted from the experiments as it has an almost identical N-terminus to CrpA. The processing of CrpA was more limited in a CrpAC coexpression compared to a CrpABC coexpression, but up to 3 modifications were still observed. After 5 residues were removed from the N-terminus of CrpA (i.e., the predicted RRE recognition sequence), the extent of processing decreased, and with additional truncations up to 20 AA, processing was reduced even further, resulting in only the 6×IAA and 5×IAA species present in substantial abundance. Removal of the full CrpA N-terminal domain and the CrpC RRE domain did not completely abolish processing, as a portion of CrpA was modified at least once (Figure S27). This suggests that substrate-enzyme interactions may be facilitated by domain–domain interactions rather than exclusively by RRE-leader binding.

## Discussion

Methanogens are intriguing organisms for a variety of reasons, including their ability to perform methanogenesis. A hallmark of these organisms is their relatively high cysteine content compared to other prokaryotes (Figure 1).^54^ We performed a deeper bioinformatic investigation into high-cysteine archaea and uncovered a group of cysteine-rich proteins adjacent to RS enzymes. While our method prioritized high cysteine content in proteomes and proteins, we envision that this method could be adapted to search for other protein/proteome sequence features of interest. Similar BGCs are also observed across all domains of life, providing an opportunity for further discovery and characterization of these protein products (Supplemental Data set 1). CrpA, CrpB, and CrpC are clustered together in a RiPP-like BGC; however, further work will need to identify if there are other proteins in the vicinity of these genes that could be involved in further substrate modification, proteolysis, or transport. Currently, the surrounding genes have not been biochemically verified, although an adjacent protein is annotated as a periplasmic copper-binding protein, which could signify BGC involvement in metal transport.

Here, we demonstrate that CrpC is an RS enzyme. Through site-directed mutagenesis and spectroscopy, we established that CrpC ligates three [4Fe-4S] clusters, one in the RS active site and two in an auxiliary SPASM domain. Although the role of these auxiliary clusters remains enigmatic, we show that the AuxI cluster is necessary for enzymatic activity, allowing us to postulate that this cluster could be involved in electron shuttling as observed with previous RS enzymes.^30,31,33^ The SPASM domain in CrpC contains seven cysteines, three of which are predicted to coordinate the AuxI cluster, and four of which are predicted to coordinate the AuxII cluster. Like the active site RS cluster, this coordination environment for the AuxI cluster would leave a unique iron in the cluster that could coordinate a cysteine residue in the substrate.^31,32^ Although AuxII does not seem to be necessary for CrpC enzymatic activity, AuxII cluster alanine variants do show decreased activity compared to the WT enzyme. This cluster is predicted to reside close to the surface of the protein, which could potentially support a role in electron transport as well.

Using chemical derivatization and mass spectrometry, we show that CrpC installs an intramolecular thioether bond on CrpA. Inclusion of reducing agent in samples, observation of daughter ions harboring a single cysteine and a −2 Da mass loss, and IAA alkylation results refute disulfide bond formation. Furthermore, postdesulfurization, fragmentation was observed through the modified motifs demonstrating that the crosslink was lost, which would not be observed with C–C and other non-thioether crosslinks. Thioether crosslinks in peptides are common in RiPP pathways, but instead of producing a modified peptide, the Crp BGC produces a modified protein (MW: 28,766 Da), of which the final fully modified structure requires further investigation. In this way, the Crp product shares similarity to QHNDH, MCR, and the *E. coli* ribosome as examples of proteins with RiPP-like modifications.^47,49,87–89^ Cysteine residues consistently precede putative acceptor residues in CrpA which suggests that each motif enters the CrpC active site in a similar manner; however, the exact order of processing for the full protein is still uncertain. The biological function of modified CrpA also remains unclear, but BGC upregulation when *M. voltae* is grown on pyrite suggests that it may bind metals.^71–73^ Further research will be required to uncover the biological function of CrpA.

RiPP-RS maturases install two different types of thioether bonds, generating either sactionine or ranthionine crosslinks. Current data suggest that CrpC is a ranthisynthase, due to the lack of MS fragmentation through the modified CrpA motifs and the loss of residues within a modified motif without loss of the crosslink (Figure 5B). Tandem MS thioether dissociation is a defining characteristic of sactionine crosslinks and was not observed.^46^ These results also suggest that the AlphaFold3-predicted β-helix for CrpA may not be accurate as the bond distances are too far apart with the cysteine and acceptor residues on opposite faces of the predicted β-helix. However, the C-terminal region is low confidence, limiting firm conclusions on the structure of the final product (Figure S5). Furthermore, CrpA-CrpC binding may be a combination of RRE-leader and domain–domain interactions. Although protein–protein substrate-enzyme engagement is relatively rare in RiPPs, it is not unprecedented.^34,36,90,91^ An AlphaFold3 multimer model for CrpA-CrpC shows the lowest error between the RRE and N-terminus of CrpA suggesting a more extensive interaction than just the short RRE recognition sequence (Figure S26). Thus, this work provides an additional example of noncanonical RRE binding and recognition in RiPPs. As CrpB has an identical N-terminal sequence to CrpA, it is possible that CrpB would bind in a similar manner. It is possible that CrpB could act as a chaperone and facilitate binding between CrpA and CrpC since the extent of modification observed was higher when CrpB was included in *in vivo* coexpressions, but further research will be required to either confirm or refute that hypothesis.

## Conclusion

In this report, we bioinformatically identified a group of high-cysteine archaea with cysteine-rich proteins adjacent to RS enzymes and selected a BGC from *M. voltae* (Crp) to biochemically characterize. Functional and structural characterization of the Crp proteins demonstrated that CrpC is an active SPASM-containing RS enzyme that modifies a protein substrate, CrpA, to install thioether crosslinks. Our work adds to an expanding group of RiPP-like modifications on proteins, describes a proteome to BGC prioritization workflow, and paves the way for additional structural and functional analysis of CrpA.

## Materials and Methods

### General Materials and Methods

Unless otherwise noted, chemical reagents were purchased from Sigma-Aldrich, Gold Biotechnology, or Thermo Fisher Scientific. Reagents for molecular biology experiments were purchased from New England Biolabs. *E. coli* DH5α was used for plasmid maintenance and molecular cloning. An Avanti JXN-26 centrifuge (Beckman Coulter) was used for cell harvesting, and a Sorvall RC6 Plus centrifuge (Thermo Fisher Scientific) was used for lysate centrifugation during protein purifications. MALDI-TOF-MS data were collected with an UltrafleXtreme instrument (Bruker Daltonics) at the Mass Spectrometry Core at Vanderbilt University.

### Bioinformatic Analysis of Cysteine-Rich Proteins

Protein FASTA files for bacterial reference genomes (*n* = 21,439), archaeal reference genomes (*n* = 756), and all archaeal RefSeq-annotated genomes (*n* = 2,660) were downloaded from NCBI.^60^ A custom Python script (Note S1) was used to calculate and compile the cysteine content of each proteome. A cutoff of 1% was used to differentiate high cysteine archaea (>1%) from low cysteine archaea (<1%) based on the sample distribution (Figure 1). The high- and low-cysteine archaeal proteomes were then separated and analyzed via HMMER^92^ to determine the presence or absence of the McrABCDG (Methyl-coenzyme M reductase) and MtrABCDEGH (N^5^-methyltetrahydromethanopterin:coenzyme M methyltransferase) proteins. If an organism encodes all selected proteins, it was classified as a methanogen. If any proteins were missing, the organism was classified as a nonmethanogen. The *p*-value between the high- and low-cysteine proteome data sets was calculated with Fisher’s exact test.

All cysteine-rich proteins (protein length ≥100 AA and cysteine frequency ≥10%, or protein length ≥250 AA and cysteine frequency ≥5%), in every high-cysteine archaeal proteome were collected and tabulated with a custom Python script (Note S2). After analysis, 6,743 unique proteins met the stated conditions and were analyzed via RODEO.^93^ Protein annotation and co-occurrence were determined via the RODEO output files. Unannotated cysteine-rich proteins were filtered to collect only those in which a BGC contained a predicted RS enzyme (PF04055). The associated RS enzymes were analyzed with a SSN generated by the enzyme function initiative-enzyme similarity tools (EFI-EST) and visualized with Cytoscape 3.10.2.^94–96^

CrpC (UniProt: D7DT95) was submitted to radicalSAM.org^78^ to examine conserved cysteines among similar RS enzymes. CrpC was also submitted to HHpred^79^ for domain analysis. Cysteine residues from CrpC were matched to the canonical RS cluster binding motif, as well as the cysteine motif identified in RS enzymes that contain SPASM domains. CrpAC individual and multimer protein structure models were generated using AlphaFold3^74^ and visualized in ChimeraX 1.10.1.^97^

CrpA homologs were collected via BLAST-P,^77^ with default settings, and analyzed by RODEO.^93^ Accession codes assigned to incomplete protein sequences were removed. The accessions, sequences, annotations, cysteine percentage, taxonomy, and nearby RS enzymes were tabulated and deposited in Supplemental Data set 1.

### Generation of Crp Plasmids and Variants

The nucleotide sequences for *crpA*, *crpB,* and *crpC* from *M. voltae* were optimized for *E. coli* expression then synthesized by a commercial vendor (Integrated DNA Technologies; IDT). *crpC* was inserted into a modified pET19b vector (EMD Millipore), while *crpA* and *crpB* were inserted into a modified pCDF vector (EMD Millipore). Synthesized genes were inserted using Gibson assembly^98^ between the NdeI and BamHI restriction sites for pET19b, and the NcoI and XhoI sites in pCDF. Additionally, a pETduet vector was synthesized by Genewiz that contained *E. coli*-optimized sequences for His_10_-CrpC and His_6_-MBP-CrpA. To generate CrpC [4Fe-4S] Ala cluster variants, Gibson assembly^98^ was used to insert commercially synthesized DNA (IDT) into the corresponding expression plasmids. CrpAC leader truncations were generated by site-directed mutagenesis (Kinase Ligase DpnI; NEB) according to manufacturer’s specifications. All plasmids were sequence-verified by Functional Biosciences or Plasmidsaurus. Supplemental Data set 2 contains DNA sequences, primers, synthetic DNA, and final protein sequences used in this study.

### CrpC Expression and Purification

Plasmids with CrpC WT, alanine cluster variants, CrpA, or CrpB were transformed into *E. coli* BL21 DE3 pLysS Rosetta cells (EMD Millipore). A single colony from these plates were used for starter cultures in LB medium supplemented with 50 μg/mL carbenicillin and 33 μg/mL chloramphenicol for CrpC cultures, and 33 μg/mL chloramphenicol, and 50 μg/mL spectinomycin for CrpA and CrpB culture. The overnight cultures were used to inoculate 9 L of LB medium in six 2.8 L Fernbach flasks, each containing 1.5 L of LB medium. CrpC cultures (WT or Ala cluster variants) were grown at 37 °C with 200 rpm shaking until OD_600_ ∼0.3 was reached. Cultures containing CrpC WT, or Ala cluster variants were then transferred to an ice bath for 1 h, then induced with 1 mM isopropyl-β-d-1-thiogalactopyranoside (IPTG), and supplemented with 300 μM ferrous ammonium sulfate (FAS) and 300 μM l-cysteine. Following induction, cultures were returned to the shaker incubator and allowed to express overnight at 18 °C, 200 rpm. For CrpA cultures, once cells reached OD_600_ ∼0.3, protein expression was induced with 1 mM IPTG, then cultures were grown for 3 h at 37 °C, 200 rpm. Cultures containing CrpB were grown to a density of OD_600_ ∼0.5 at 37 °C with 200 rpm shaking, then induced with 1 mM IPTG, then allowed to express for 3 h at 37 °C. Cultures were then transferred to 4 °C and sparged with N_2_ gas overnight.

For the triple expression cultures, the pETDuet plasmid containing His_10_-CrpC and His_6_-MBP-CrpA were cotransformed with a pCDF plasmid containing His_10_-CrpB into *E. coli* BL21 DE3 pLysS Rosetta cells. A single colony from these plates was used for starter cultures in LB medium supplemented with 50 μg/mL carbenicillin, 33 μg/mL chloramphenicol, and 50 μg/mL spectinomycin. Overnight cultures were used to inoculate LB cultures in 2.8 L Fernbach flasks. Cultures were grown to a density of OD_600_ ∼0.3 at 37 °C at 200 rpm then supplemented with 300 μM FAS and 300 μM l-cysteine. Cells were then returned to 37 °C with 200 rpm shaking and allowed to grow until they reached a density of OD_600_ ∼0.8. Cultures were then induced with 1 mM IPTG, then allowed to express at 18 °C and 50 rpm shaking overnight. Cells from all cultures were harvested by centrifugation (5,000 rpm, 4 °C, 15 min), then flash-frozen in liquid nitrogen and stored at −80 °C until purification.

At the time of purification, cell pellets were brought into an anaerobic chamber (Coy Laboratory Products). Pellets containing CrpC were resuspended in anaerobic Tris lysis buffer (75 mM Tris, pH 8, 100 mM KCl, 5% glycerol with 25 mg/mL MgCl_2_, 0.2 mg/mL DNase, 0.2 mg/mL lysozyme, protease inhibitor tablet (Sigma) and 1% Triton-X added fresh). The cell pellets were allowed to thaw in the lysis buffer for 1 h, then lysed by sonication for 5 min (5 s on/15 s off pulses, 40% amplitude). The lysate was then transferred to a sealable centrifuge tube, removed from the chamber, and clarified by centrifugation (26,790 × g, 30 min, 4 °C), then reintroduced into the Coy chamber. All CrpC constructs contain an N-terminal His-tag, which was used to bind the protein to a 5 mL HisTrap FF Ni-NTA column (GE Healthcare) in Tris buffer A (75 mM Tris, pH 8, 100 mM KCl, 5% glycerol) using an AKTA Pure FPLC (GE Healthcare). Following loading at 0% B, the column was washed with 20% B, then the protein was eluted with 80% Tris elution buffer B (75 mM Tris, pH 8, 100 mM KCl, 5% glycerol, and 500 mM imidazole). Eluted protein was buffer-exchanged into storage buffer (75 mM Tris, pH 8, 100 mM KCl, 5% glycerol) using a HiPrep Desalting column (Cytiva), then concentrated using an Amicon Ultra 30 kDa molecular weight cutoff (MWCO) centrifugal filter (MilliporeSigma) and stored at −80 °C.

Purifications of CrpA followed the above procedure with slight modifications. The cell pellets were thawed in MBPTrap lysis buffer (20 mM Tris, pH 7.4, 200 mM NaCl, 1 mM EDTA with 1 mM DTT, with 25 mg/mL MgCl_2_, 0.2 mg/mL DNase, 0.2 mg/mL lysozyme, protease inhibitor tablet (Sigma) and 1% Triton-X added fresh). Following sonication and centrifugation, lysates were loaded onto a 5 mL MBPTrap HP column (Cytiva) in MBPTrap buffer A (20 mM Tris, pH 7.4, 200 mM NaCl, 1 mM EDTA with 1 mM DTT added fresh). Following loading, bound proteins were eluted with 100% MBPTrap buffer B (20 mM Tris, pH 7.4, 200 mM NaCl, 1 mM EDTA, 10 mM maltose with 1 mM DTT added fresh). For purifications that were expressed from a single protein expression, the eluate was buffer-exchanged into Tris storage buffer then concentrated using an Amicon Ultra 30 kDa MWCO centrifugal filter and stored at −80 °C. For purifications of CrpA from the triple expression system, following buffer exchange, the eluate was loaded onto a 5 mL HisTrap FF Ni-NTA column using the same conditions above, then eluted with 20% Tris elution buffer. Protein was exchanged into storage buffer using a HiPrep desalting column, then concentrated using an Amicon Ultra 30 kDa MWCO centrifugal filter, and stored at −80 °C.

### Reconstitution of CrpC WT and Ala Cluster Variants

All reconstitutions were performed in an anaerobic chamber. Reagents (DTT, FAS, and Li_2_S) were brought into the chamber in powder form and then dissolved in storage buffer. DTT (1 mM) was added to the purified protein (100 μM) and stirred at room temperature. For WT CrpC, 12 mol equivalents of FAS (10 mM stock solution) and Li_2_S (50 mM stock solution) were added in 5 aliquots over 15 min. For single-cluster Ala variants, 8 mol equivalents were added, and double Ala cluster variants were mixed with 4 mol equivalents. Reactions were allowed to stir for 3 h, then the solution was centrifuged to remove particulates. The supernatant was then buffer-exchanged into storage buffer using a PD-10 column (GE Healthcare). Protein was concentrated using an Amicon Ultra 30 kDa MWCO centrifugal filter, aliquoted, flash-frozen, and stored at −80 °C.

### Iron and Sulfur Quantification

Protein concentrations were determined using a Bradford assay.^99^ Iron quantitation was completed using a SpectrAA 220 Fast Sequential Atomic Absorption Spectrometer (Varian). An iron AA standard (Bicca, 1000 ppm in 3% HCl) was used to generate a calibration curve by creating standards ranging from 0.4 mg/L to 2.0 mg/L. Protein was diluted to 1 μM in triplicate, then assayed using the spectrometer. Sulfur was quantified using Beinert’s method following previously published methods.^80^

### Preparation of CrpC for Mössbauer Spectroscopy

^57^Fe was purchased from Cambridge Isotope Laboratories, Inc., dissolved in hot, concentrated hydrochloric acid, and the pH was adjusted to 7.0–7.2 with NaOH. An overnight starter culture containing *E. coli* BL21 (DE3) pLysS Rosetta cells transformed with CrpC WT was used to inoculate 1.5 L of minimal media, with 300 μM ^57^Fe added upon induction. Lysis, purification, and reconstitution steps were as described above, with ^57^Fe used rather than ^56^Fe. Protein samples (623.1 μM as purified and 387.6 μM for the reconstituted protein) were loaded into 450 μL Delrin Mössbauer cups and immediately stored in liquid nitrogen. Spectra were collected with home-built spectrometers using Janis Research Super-Varitemp dewars.

### Size-Exclusion Chromatography of CrpC Variants

Analytical size-exclusion chromatography (SEC) of CrpC variants was carried out anaerobically using a HiPrep 16/60 Sephacryl S-200 High Resolution column with a flow rate of 0.5 mL/min in 75 mM Tris, pH 8, 100 mM KCl, and 5% glycerol buffer. A standard curve was prepared using Gel Filtration Standards (Biorad) which contained protein standards for 670 kDa (thyroglobulin), 158 kDa (γ-globulin), 44 kDa (ovalbumin), 17 kDa (myoglobin), and 1.35 kDa (vitamin B_12_). Injections of 250 μL containing 20 μM protein for each variant were used for analysis.

### Enzyme Activity Assays

All activity assays were carried out in a Unilab glove box (O2 ≤ 1 ppm; MBraun). SAM was prepared enzymatically as previously described.^13^ In the glove box, 20 μM of the CrpC constructs were transferred to a clean Eppendorf tube containing storage buffer. Protein was reduced for 10 min following the addition of 1 mM DTT and 200 μM sodium dithionite (NaDT). Following reduction, 200 μM SAM with or without 100 μM substrate (CrpA) was added to initiate the reaction. The reaction was allowed to proceed for 3 h, then quenched with 1:1 (v/v) of 1 M acetic acid. Samples were kept on ice for 30 min to allow protein to precipitate, then centrifuged twice at 14,000 rpm for 10 min. The resulting supernatant was transferred to a clean HPLC vial and stored at −20 °C then analyzed on a Shimadzu LC-2060C liquid chromatograph equipped with a reversed-phase column (Phenomenex Kinetex F5, 2.6 μm, 100 Å, 150 × 4.6 mm) equilibrated in 98% solvent A (water with 0.1% formic acid) and 2% solvent B (acetonitrile with 0.1% formic acid). The column was washed with 2% solvent B at 1 mL/min for 3.5 min, followed by a linear gradient, reaching 100% solvent B at 11.5 min. 100% solvent B was maintained for 2.5 min prior to re-equilibrating the column with 2% solvent B. SAM eluted in the void volume, while 5′-dAdoH eluted with a retention time near 6.1 min. SAM and 5′-dAdoH were detected by their absorbance at 254 nm. 5′-dAdoH eluates were identified by comparison of their retention times to those of a commercially available 5′-dAdoH standards that were run separately or by standard addition to assay samples. Quantification was carried out by integrating peaks and generating standard curves using GraphPad Prism software. To generate chromatograms, the raw chromatographic data was processed in the LabSolutions software by subtracting a blank run from each data set.

### Electron Paramagnetic Resonance Spectroscopy

EPR spectroscopy followed the general procedures described previously.^100^ Continuous-wave X-band EPR spectroscopy was performed using a Bruker EMX EPR spectrometer equipped with a Bruker/ColdEdge (Sumitomo Cryogenics) 10 K waveguide cryogen-free system with an Oxford Mercury iTC controller unit and a helium Stinger recirculating unit (Sumitomo Cryogenics, ColdEdge Technologies). For analysis of iron–sulfur clusters, CrpC constructs were reduced with 10 equiv of NaDT and allowed to sit for 10 min. Samples were either transferred to EPR tubes, capped, and flash-frozen in liquid nitrogen outside the glove box, or 30 equiv of SAM was added, then the samples were transferred, capped, and frozen. Each sample was measured at varying temperatures and powers as indicated in figure captions. Microwave signals were recorded with a microwave frequency of 9.38 GHz, microwave power of 1 mW, receiver gain of 10,000, modulation frequency of 100 kHz, modulation amplitude of 10 G, and time constraint of 168.84 ms, with a scanning range from 3,000 to 4,000 G. EPR simulations were performed with EasySpin.^81^ All spectra were cavity-subtracted and baseline-corrected.

### Protease Digests and MALDI Mass Spectrometry

Purified *in vivo* CrpABC and CrpAC coexpressions and *in vitro* reactions were treated with 5 mM TCEP for 30 min at room temperature. Next, samples were either treated with 15 mM iodoacetamide (IAA) for 1 h, at room temperature, in the dark, or treated directly with trypsin as described below. The reaction was quenched with 15 mM DTT. Alkylated CrpA samples were then digested with tobacco etch virus protease (TEV) for 1 h at room temperature to remove the MBP tag from CrpA. As both the MBP-tag and TEV protease contained a His_6_-tag, they were removed via Ni-NTA purification (Thermo Fisher). The CrpA TEV digests were loaded onto pre-equilibrated Ni-NTA resin and allowed to equilibrate at room temperature for 5 min. The flowthrough was collected, and then the column was washed with 5 column volumes of Ni-NTA wash buffer (30 mM imidazole, 0.5 M NaCl, 50 mM Tris, pH 7.5), which was also collected (full-length CrpA was found in both the FT and wash fractions). CrpA samples were next treated with trypsin for up to 5 h according to the manufacturer’s specifications (Promega). After protease digestion, a small amount of each sample was desalted with a C18 ZipTip (EMD Millipore), eluted into 60% acetonitrile, and mixed 1:1 with 50 mg/mL super DHB (dissolved in 60% acetonitrile with 0.1% formic acid) matrix for MALDI-TOF-MS analysis. Trypsin-digested samples, prepared as described above, were also submitted directly for LC-MS.

### Desulfurization and Deuterium Labeling of CrpA

MBP-CrpA was purified from a 2 L coexpression with CrpBC and digested with TEV overnight (1:100 enzyme:MBP-CrpA) at room temperature. Following proteolysis, the digest was mixed with pre-equilibrated HisPur Ni-NTA resin (Thermo Fisher) to remove MBP and TEV as described earlier. The resulting flowthrough and wash fractions were buffer-exchanged into 50 mM ammonium bicarbonate, pH 8, with Amicon Ultra 10 kDa MWCO centrifugal filters. Next, the TEV-digested and buffer-exchanged CrpA proteins were treated with sequencing-grade trypsin (Promega, 1:100 enzyme:substrate) for 5 h at 37 °C. Successful proteolysis was confirmed by MALDI-TOF-MS. After trypsin digestion, the samples were flash-frozen in liquid nitrogen and lyophilized to dryness overnight. The approximate mass of trypsin-digested CrpA (considering all tryptic fragments, not just the C-terminal fragment of interest) was 1 mg.

The desulfurization reaction workflow was adapted from previous work on sactipeptides.^50,86^ Once dry, the samples were redissolved in 200 μL of a 60% methanol-D_4_:40% D_2_O solution. To a screw-capped 2 mL vial (already flushed with nitrogen), 3 mg of anhydrous NiCl_2_ (Thermo Fisher) was added followed by the redissolved CrpA solution. Immediately, 3 mg of NaBD_4_ (MilliporeSigma) was added into the vial, then the vial was quickly sealed. Successful formation of a nickel boride catalyst was observed by the formation of a black particulate. The reaction vial was heated for 5 min at 50 °C. Two additional 3 mg aliquots of NaBD_4_ were added to the reaction vial with 5 min of heating at 50 °C in between. After completion, the reaction was quenched with 30 μL of trifluoroacetic acid (TFA). To remove the black particulate, the reaction was transferred to a 1.7 mL Eppendorf tube and centrifuged at 10,000 × g for 5 min. The supernatant was collected and neutralized on ice with 400 μL of 1 M ammonium bicarbonate in 50 μL aliquots. The sample was dried via lyophilization overnight, then reconstituted in 100 μL of 1% methanol in water and analyzed directly by LC-MS/MS.

### LC-MS/MS Methods

All LC-MS/MS methods were performed with a Thermo Fisher Scientific Vanquish UHPLC coupled to a Q Exactive HF MS. The MS was calibrated and tuned with Pierce LTQ ESI Positive Ion Calibration Solution (Thermo Fisher). The MS was operated using the following parameters: Full MS: polarity, positive; scan range, 150–2000 m/z; resolution, 120,000. Parallel reaction monitoring (PRM, MS/MS): polarity, positive; isolation width, 0.5–1 *m/z*; normalized collision energy (NCE), 25. Data analysis was conducted using the Qual Browser application of Xcalibur software (Thermo Fisher Scientific), Protein Prospector, Annotator,^101^ SIRIUS,^102^ and ClipsMS.^103^

Mobile phases of A: H_2_O + 0.1% formic acid (FA) and B: acetonitrile +0.1% FA were used. Samples were run on a Hypersil Gold C18 analytical column (50 × 2.1 mm, 1.9 μm particle size). A flow rate of 0.3 mL/min was used for all injections. The column was equilibrated at 5% B in between injections. Linear gradients were used for all methods. *IAA-alkylated and trypsin-digested CrpA*: *t* = 0 min, 5% B; *t* = 2 min, 5% B; *t* = 4 min, 15% B; *t* = 20 min, 55% B; *t* = 21 min, 90% B; *t* = 22 min, 90% B; *t* = 25 min, 5% B. MS was collected between 2 and 22 min. Molecular ions of interest were selected for fragmentation with PRM. *Trypsin digested CrpA postdesulfurization*: *t* = 0 min, 5% B; *t* = 2 min, 5% B; *t* = 14 min, 60% B; *t* = 15 min, 90% B; *t* = 17 min, 90% B; *t* = 18 min, 5% B. MS was collected between 2 and 17 min. Molecular ions of interest were selected for fragmentation with PRM.

## Supplementary Material







## Notes

Supporting figures (S1–S27), and supporting tables (S1–S17) (PDF)
CrpA homologs retrieved by BLAST-P (XLSX)
Oligonucleotide primers and plasmid sequences used in this study (XLSX)
