# Supplementary material for: Protein Thioether Crosslinks Installed by a SPASM-Containing Radical SAM Enzyme from Methanogenic Archaea
Source: ACS Bio Med Chem Au. 2026 Sep 21;6(5):694–707. doi: 10.1021/acsbiomedchemau.6c00133 (PMC13692751; doi:10.1021/acsbiomedchemau.6c00133)
Supplement: Supplementary file 1 [file bg6c00133_si_001.pdf]

Supporting Information for:

Protein Thioether Crosslinks Installed by a SPASM-Containing Radical SAM Enzyme from Methanogenic Archaea

Emily M. Dieter[1,2‡], Austin M. Woodard[3,4‡], Jared C. Green[1], Jin Xiong[5], Yisong Guo[5], William E. Broderick[1,6], Douglas A. Mitchell[4,7*], and Joan B. Broderick[1,6*].

[1]Department of Chemistry and Biochemistry, Montana State University, Bozeman, MT 59717, United States

[2]Department of Chemistry and Biochemistry, Gettysburg College, Gettysburg, PA 17325, United States

[3]Department of Chemistry, University of Illinois at Urbana-Champaign, Urbana, IL 61801, United States

[4]Department of Biochemistry, Vanderbilt University School of Medicine, Nashville, TN 37232, United States

[5]Department of Chemistry, Carnegie Mellon University, Pittsburgh, PA 15213, United States

[6]Department of Chemistry, Texas A&M University, College Station, TX 77843, United States

[7]Department of Chemistry, Vanderbilt University, Nashville, TN 37232, United States

‡ These authors contributed equally

*Corresponding Authors

Douglas A. Mitchell (douglas.mitchell@vanderbilt.edu)

Joan B. Broderick (joan_broderick@tamu.edu)

Table of Contents:

Note S1: Per-proteome cysteine content analysis python code 5
Note S2: Proteome-wide protein cysteine content calculation and tabulation python code...........6
Figure S1: Thioether modifications in RiPPs and QHNDH 7
Table S1: Analysis of high and low cysteine archaea proteomes for methanogen assignment8
Figure S2: Bioinformatic discovery of archaeal cysteine-rich proteins...9
Table S2: Retrieved HMMs for identified archaeal cysteine-rich proteins 10
Table S3: Top co-occurring HMMs adjacent to archaeal cysteine-rich proteins 11
Table S4: Descriptions of RS enzymes associated with archaeal cysteine-rich proteins 13
Figure S3: Archaeal BGCs with cysteine-rich proteins and RS enzymes 14
Figure S4: Sequence analysis of CrpA and CrpB 15
Figure S5: AlphaFold3 predicted structure of CrpA...16
Figure S6: SDS-PAGE of purified CrpC and CrpA 17
Figure S7: Line diagrams of CrpC [4Fe-4S] cluster variants 18
Figure S8: Size exclusion chromatography of CrpC WT and [4Fe-4S] cluster variants..............19
Table S5: Quantified iron and sulfur content of CrpC constructs 20
Figure S9: EPR spectroscopic characterization of WT CrpC 21
Figure S10: Isotopic distribution analysis of modified CrpA..22
Figure S11: LC-MS chromatograms of IAA-labeled and trypsin-digested CrpA 23
Figure S12: HR-LC-MS/MS analysis of 6×IAA labeled CrpA tryptic fragment 24
Table S6: Assignment of HCD daughter ions for the 6×IAA-labeled CrpA tryptic fragment......26
Figure S13: HR-LC-MS/MS analysis of 5×IAA labeled CrpA^{C2}-tryptic fragment 28
Table S7: Assignment of HCD daughter ions for the 5×IAA-labeled CrpA^{C2}-modified tryptic fragment 30
Figure S14: HR-LC-MS/MS analysis of 5×IAA labeled CrpA^{C7}-modified tryptic fragment.......32
Table S8: Assignment of HCD daughter ions for the 5×IAA-labeled CrpA^{C7}-modified tryptic fragment 34
Figure S15: HR-LC-MS/MS analysis of 5×IAA labeled CrpA^{C28}-modified tryptic fragment 35
Table S9: Assignment of HCD daughter ions for the 5×IAA-labeled CrpA^{C28}-modified tryptic fragment 37
Figure S16: HR-LC-MS/MS analysis of 4×IAA labeled CrpA^{C2C7}-modified tryptic fragment....39

Table S10: Assignment of HCD daughter ions for the 4×IAA-labeled CrpA^{C2C7}-modified tryptic fragment 41
Figure S17: HR-LC-MS/MS analysis of 4×IAA labeled CrpA^{C2C28}-modified tryptic fragment 43
Table S11: Assignment of HCD daughter ions for the 4×IAA-labeled CrpA^{C2C28}-modified tryptic fragment 45
Figure S18: HR-LC-MS/MS analysis of 4×IAA labeled CrpA^{C7C28}-modified tryptic fragment 47
Table S12: Assignment of HCD daughter ions for the 4×IAA-labeled CrpA^{C7C28}-modified tryptic fragment 49
Figure S19: HR-LC-MS/MS analysis of 3×IAA labeled CrpA^{C2C7C28}-modified tryptic fragment ... .51
Table S13: Assignment of HCD daughter ions for the 3×IAA-labeled CrpA^{C2C7C28}-modified tryptic fragment...53
Figure S20: HR-LC-MS/MS analysis of 2×IAA labeled CrpA^{C2C7C13C28}-modified tryptic fragment 55
Table S14: Assignment of HCD daughter ions for the 2×IAA-labeled CrpA^{C2C7C13C28}-modified tryptic fragment...57
Figure S21: Summary of CrpA desulfurization 58
Figure S22: HR-LC-MS/MS analysis of unmodified and trypsin-digested CrpA after reaction post desulfurization 59
Table S15: Assignment of HCD daughter ions for unmodified and trypsin digested CrpA post desulfurization...61
Figure S23: HR-LC-MS/MS analysis of modified and trypsin-digested CrpA post desulfurization 62
Table S16: Assignment of HCD daughter ions for modified and trypsin digested CrpA post desulfurization...64
Figure S24: Analysis of isotopic ratios for assignment of selected HCD daughter ions for trypsin digested CrpA post desulfurization..65
Table S17: Comparison ^{13}C isotope and deuterium labeling *m/z* errors for selected daughter ions for modified and trypsin-digested CrpA post desulfurization 67

Figure S25: Catalytic proximity profiling of CrpC...68
Figure S26: AlphaFold3 multimer structures of CrpAC...69
Figure S27: Mass spectrometry analysis of CrpAC N-terminal truncations 70
References..71

Note S1: Per-proteome cysteine content analysis python code

```
import os
from Bio import SeqIO
import csv

OUTPUT_FILE = 'archaea_per_genome_cys_content_testing.csv'
proteome_dict = {}
proteome_cys_content_list = []
def genome_cys_content_calculation(directory):
    # directory is the filepath to the directory with all of the genomes. If downloading from
NCBI datasets, this is the "data" directory
    for root, dirs, files in os.walk(directory):
        for file in files:
            filepath = os.path.join(root, file)
            root_split = root.split("data/")
            proteome_ID = root_split[1]
            total_proteome_length = 0
            proteome_cys_count = 0
            if file.lower().endswith('.faa'):
                #can replace with different type of fasta/sequence file extension
                for record in SeqIO.parse(filepath, "fasta"):
                    protein_length = len(record.seq)
                    protein_sequence = record.seq.upper()
                    proteome_cys_count += protein_sequence.count("C")
                    #this can be changed to any other residue or feature of interest for
different analyses
                    total_proteome_length += protein_length

                proteome_cys_content = round((proteome_cys_count/total_proteome_length)*100,
4)
                proteome_dict['Proteome'] = proteome_ID
                proteome_dict['Total_Length'] = total_proteome_length
                proteome_dict['Total_Cys_Residues'] = proteome_cys_count
                proteome_dict['Cys_Percent'] = proteome_cys_content
                proteome_cys_content_list.append(proteome_dict.copy())

    # writing the csv output for further data analysis
    with open (OUTPUT_FILE, 'w') as output:
        fields = ['Proteome', 'Total_Length', 'Total_Cys_Residues', 'Cys_Percent']
        output_writer = csv.DictWriter(output, fieldnames=fields)
        output_writer.writeheader()
        for item in proteome_cys_content_list:
            output_writer.writerow(item)

genome_cys_content_calculation('directory')
#Runs the above function. Change the input to desired directory location. See comment above as
well.
```

Note S2: Proteome-wide protein cysteine content calculation and tabulation python code

```
import csv

Cys_content_dict = {}
#This function will go through every protein, calculate the cys content, and save the output
to a new file
def calculate_cys_and_save_protein(input_file):
    with open(input_file) as input_csv:
        csv_reader = csv.DictReader(input_csv)
        data = [row for row in csv_reader]
        Cys_content_list = []
        for dict in data:
            protein_sequence = dict['sequence']
            if int(len(protein_sequence)) >= 100:
                Cys_number = protein_sequence.count("C")
                Cys_content = (Cys_number/int(len(protein_sequence)))*100
            else:
                continue

            if Cys_content >= 10 or len(protein_sequence) >= 250 and Cys_content >= 5:
                Cys_content_dict['accession'] = dict['accessions']
                Cys_content_dict['sequence'] = dict['sequence']
                Cys_content_dict['Cys_number'] = Cys_number
                Cys_content_dict['Cys_percent'] = Cys_content
                Cys_content_list.append(Cys_content_dict.copy())

        #Writes output CSV
        with open('Cys_rich_proteins.csv', 'w') as output_file:
            fields = ['accession', 'sequence', 'Cys_number', 'Cys_percent']
            output_writer = csv.DictWriter(output_file, fieldnames=fields)
            output_writer.writeheader()
            for item in Cys_content_list:
                output_writer.writerow(item)

calculate_cys_and_save_protein('fasta_to_CSV_combined.csv')
#Calculates Cys content of every protein in CSV file.
#Include full file path to CSV file
#First need to convert all fasta files to CSV files for every proteome then merge into a
single file
```

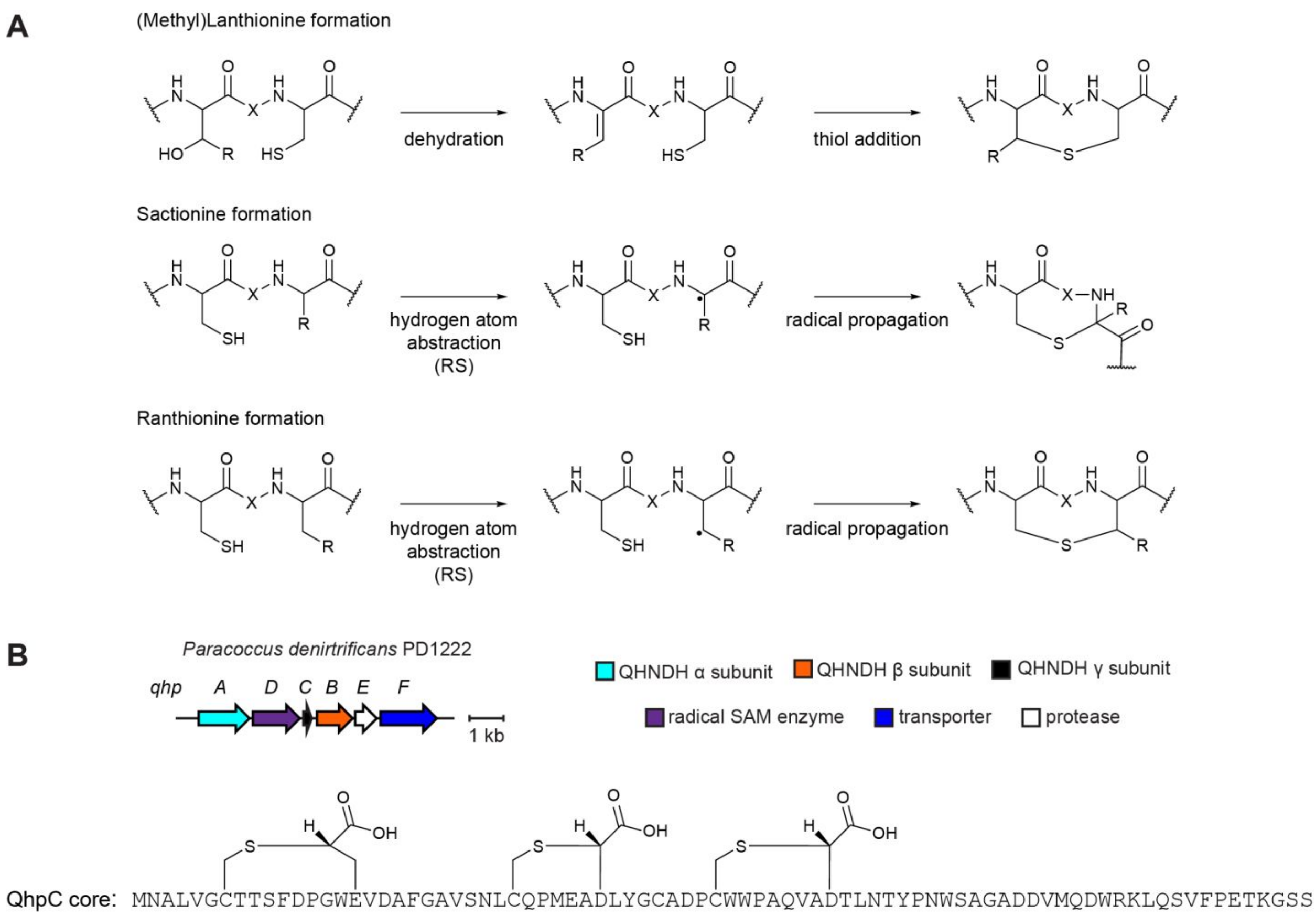

Figure S1: Thioether modifications in RiPPs and QHNDH. (A) Schematic for thioether crosslink formation among *(top-bottom)* lanthipeptides, sactipeptides, and ranthipeptides. Sactionine and ranthionine thioether crosslinks are catalyzed by RS enzymes whereas (methyl)lanthionine thioethers form via cysteine thiol addition to dehydroamino acids (R=Ser for dehydroalanine or R=Thr for dehydrobutyrine).[1–3] Stereochemistry is omitted for clarity. (B) *Top:* Quinohemoprotein amine dehydrogenase (QHNDH) BGC (*qhp*). *Bottom:* Modified core sequence of QhpC (QHNDH γ-subunit) with QhpD-installed thioether crosslinks shown.[4,5]

Table S1: Analysis of high and low cysteine archaeal proteomes for methanogen assignment. If an organism encoded all of the McrABCDG and MtrABCDGH proteins, it was classified as a methanogen. If any of the above proteins were missing, the organism was classified as a non-methanogen. There is a significant correlation between the presence of methanogens in high cysteine archaea vs low cysteine archaea ($p \ll 0.0001$).

	High cysteine archaea	Low cysteine archaea
Putative Methanogen (All Mcr and Mtr proteins present)	757	2
Putative Non-Methanogen (Missing any Mcr or Mtr proteins)	327	1574
Total Proteomes	1084	1576

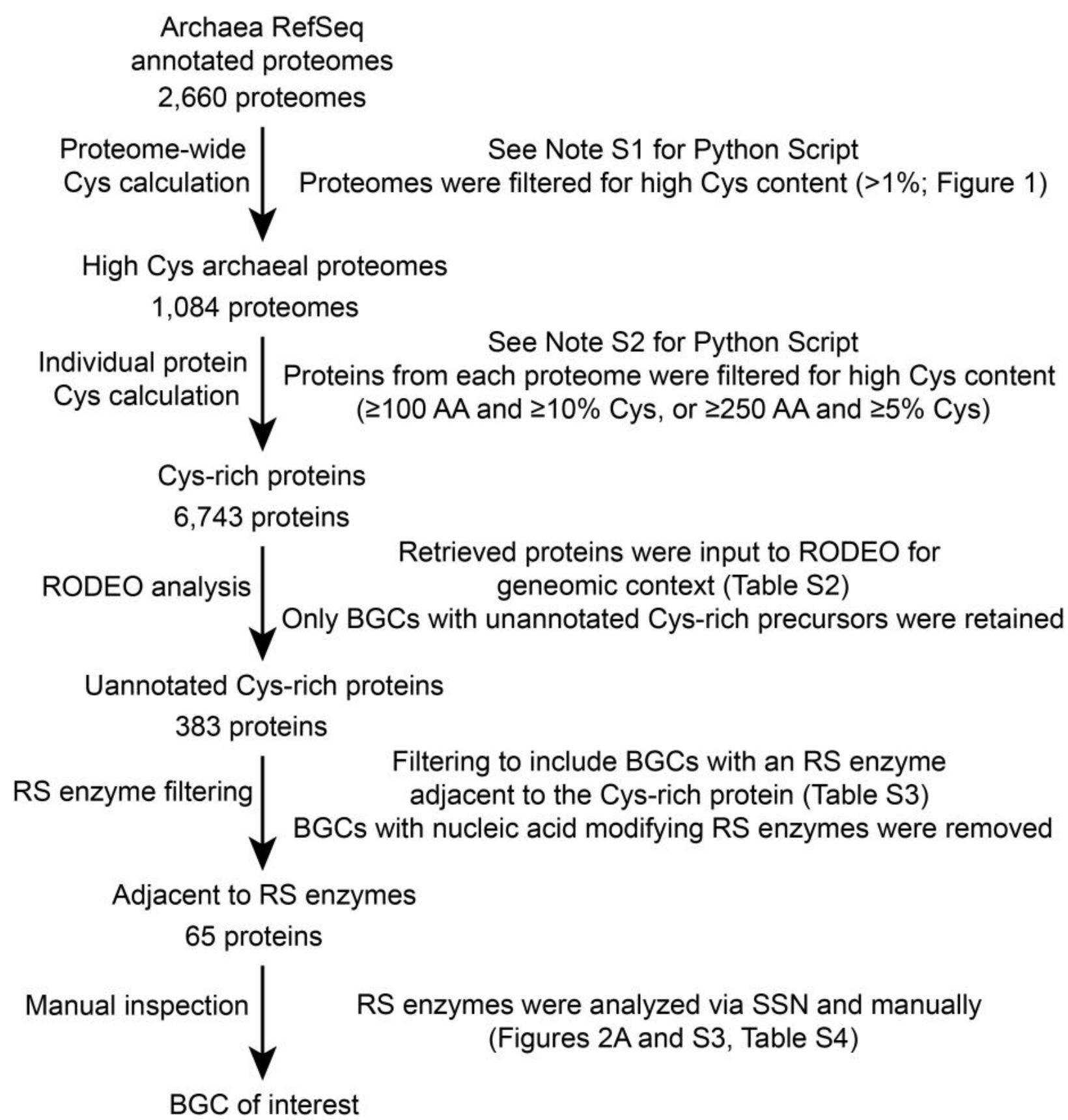

Figure S2: Bioinformatic discovery of archaeal cysteine-rich proteins. Bioinformatic workflow to identify high cysteine archaeal proteomes and cysteine-rich proteins. See **Methods** for detailed workflow.

Table S2: Retrieved HMMs for identified archaeal cysteine-rich proteins. Retrieved HMMs were collected following analysis by RODEO.[6] The number of proteins retrieved by each HMM (count) and percent of all proteins (percent) were calculated.

HMM	HMM Description	Count	Percent
PF00037	4Fe-4S binding domain	2471	36.7
TIGR03294	FrhG: coenzyme F420 hydrogenase, subunit gamma	328	4.9
PF02754	Cysteine-rich domain	301	4.5
TIGR00384	dhsB: succinate dehydrogenase and fumarate reductase iron-sulfur protein	262	3.9
PF12675	Protein of unknown function (DUF3795)	257	3.8
PF12773	Double zinc ribbon	247	3.7
TIGR02951	DMSO_dmsB: dimethylsulfoxide reductase, chain B	226	3.4
PF04432	Coenzyme F420 hydrogenase/dehydrogenase, beta subunit C terminus	183	2.71
TIGR02912	sulfite_red_C: sulfite reductase, subunit C	114	1.7
PF01000	RNA polymerase Rpb3/RpoA insert domain	101	1.5
TIGR02700	flavo_MJ0208: archaeoflavoprotein, MJ0208 family	95	1.4
PF01656	CobQ/CobB/MinD/ParA nucleotide binding domain	92	1.4
TIGR01944	rnfB: electron transport complex, RnfABCDGE type, B subunit	79	1.2
TIGR03288	CoB_CoM_SS_B: CoB--CoM heterodisulfide reductase, subunit B	77	1.1
TIGR03149	cyt_nit_nrfC: cytochrome c nitrite reductase, Fe-S protein	76	1.1
TIGR02163	napH_: ferredoxin-type protein, NapH/MauN family	73	1.1
TIGR01969	minD_arch: cell division ATPase MinD	70	1.0
Other	-	1307	19.4
No PFAM or TIGRFAM Match	-	383	5.7

Table S3: Top co-occurring HMMs adjacent to archaeal cysteine-rich proteins. Only protein HMMs with a co-occurrence greater than 10% were included. Cysteine-rich proteins with NCBI annotated functions were retained for this analysis. The number of proteins retrieved by each HMM (count) and co-occurrence frequency were calculated.

HMM	Description	Count	Co-occur
PF00037	4Fe-4S binding domain	6929	102.8
PF12838	4Fe-4S dicluster domain	4168	61.8
TIGR01971	NuoI: NADH-quinone oxidoreductase, chain I	2225	33.0
TIGR03294	FrhG: coenzyme F420 hydrogenase, subunit gamma	1750	26.0
PF01058	NADH ubiquinone oxidoreductase, 20 Kd subunit	1623	24.1
TIGR03295	frhA: coenzyme F420 hydrogenase, subunit alpha	1603	23.8
PF12837	4Fe-4S binding domain	1418	21.0
TIGR01962	NuoD: NADH dehydrogenase (quinone), D subunit	1415	21.0
TIGR01591	Fdh-alpha: formate dehydrogenase, alpha subunit	1369	20.3
TIGR01957	nuoB_fam: NADH-quinone oxidoreductase, B subunit	1301	19.3
TIGR02179	PorD_KorD: 2-oxoacid:acceptor oxidoreductase, delta subunit, pyruvate/2-ketoisovalerate family	1133	16.8
PF12773	Double zinc ribbon	1106	16.4
PF02754	Cysteine-rich domain	1096	16.3
PF00571	CBS domain	1082	16.1
PF13240	zinc-ribbon domain	1024	15.2
TIGR01302	IMP_dehydrog: inosine-5'-monophosphate dehydrogenase	1023	15.2
PF00346	Respiratory-chain NADH dehydrogenase, 49 Kd subunit	988	14.6
TIGR01944	rnfB: electron transport complex, RnfABCDGE type, B subunit	866	12.8
PF04432	Coenzyme F420 hydrogenase/dehydrogenase, beta subunit C terminus	837	12.4
TIGR03289	frhB: coenzyme F420 hydrogenase, subunit beta	837	12.4
PF00146	NADH dehydrogenase	803	11.9
PF01656	CobQ/CobB/MinD/ParA nucleotide binding domain	792	11.8
PF04422	Coenzyme F420 hydrogenase/dehydrogenase, beta subunit N-term	791	11.7

TIGR03379	glycerol3P_GlpC: glycerol-3-phosphate dehydrogenase, anaerobic, C subunit	790	11.7
PF14697	4Fe-4S dicluster domain	766	11.4
TIGR01969	minD_arch: cell division ATPase MinD	764	11.3
TIGR03710	OAFO_sf: 2-oxoacid:acceptor oxidoreductase, alpha subunit	755	11.2
PF13248	zinc-ribbon domain	745	11.1
PF04055	Radical SAM superfamily	737	10.9
TIGR02700	flavo_MJ0208: archaeoflavoprotein, MJ0208 family	726	10.8
TIGR02951	DMSO_dmsB: dimethylsulfoxide reductase, chain B	711	10.6
PF01913	Formylmethanofuran-tetrahydromethanopterin formyltransferase	706	10.5
PF02741	FTR, proximal lobe	706	10.5
TIGR03119	one_C_fhcD: formylmethanofuran--tetrahydromethanopterin N-formyltransferase	706	10.5
PF02662	Methyl-viologen-reducing hydrogenase, delta subunit	679	10.1

Table S4: Descriptions of RS enzymes associated with archaeal cysteine-rich proteins. HMMs were tabulated per SSN group (**Figure 2A;** RS enzymes associated with cysteine-rich proteins, alignment score 8). The number of RS enzymes retrieved by each HMM in the specified SSN group is indicated, showing group diversity.

SSN Group	RS HMM	HMM Description	Count/Group
1	PF04055	Radical SAM superfamily	14
2	TIGR02668	moaA_archaeal: probable molybdenum cofactor biosynthesis protein A	10
	TIGR04545	rSAM_ahbD_hemeb: heme b synthase	1
	TIGR04278	viperin: antiviral radical SAM protein viperin	1
3	TIGR04084	rSAM_AF0577: putative peptide-modifying radical SAM enzyme, AF0577 family	1
	TIGR03942	sulfatase_rSAM: anaerobic sulfatase maturase	2
	TIGR03906	quino_hemo_SAM: quinohemoprotein amine dehydrogenase maturation protein	2
	TIGR04083	rSAM_pep_methan: putative peptide-modifying radical SAM enzyme, Mhun_1560 family	1
	TIGR02666	moaA: molybdenum cofactor biosynthesis protein A	1
	TIGR04280	geopep_mat_rSAM: putative geopeptide radical SAM maturase	1
	TIGR04463	rSAM_vs_C_rich: radical SAM/SPASM domain protein maturase	1
4	TIGR04337	AmmeMemoSam_rS: AmmeMemoRadiSam system radical SAM enzyme	3
Singletons	TIGR04470	rSAM_mob_pairB: radical SAM mobile pair protein B	1
	TIGR04043	rSAM_MSMEG_0568: radical SAM protein, MSMEG_0568 family	1
	TIGR04349	rSAM_QueE_gams: putative 7-cyano-7-deazaguanosine (preQ0) biosynthesis protein QueE	1

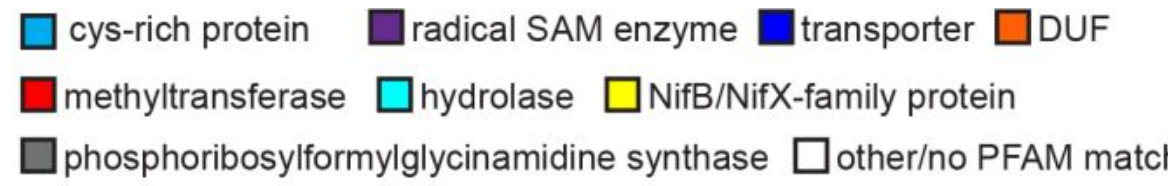

Methanimicrococcus hongohii (WP_316558037.1)

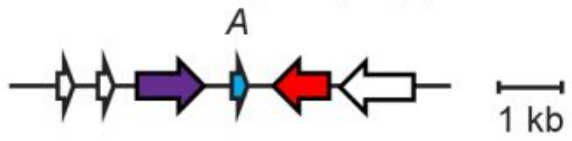


```
A: MRSVPAEPASLQLSFTATCSCWACQSATVFSCCCRSGLCFRLYLLFLIIIRS
   HSRTCRSYLQVSVCSWSEVFVCSWNQVSVSACLQVSVRQQLPPPRARRLTLS
```

Methanomethylophilus alvi (WP_022532465.1)

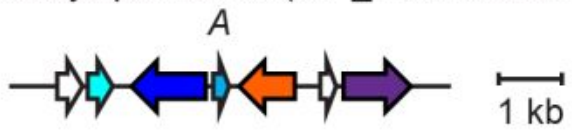


```
A: MSSIFIYNLLIDWNPHMTDERYGKCDVCRYCVHSDEDWACMINNKVVSAKQS
   CPRYRPGCCENCSHATIEFGNAVCGITKQPTDILNVCDKYDPCGRLSLNDQ
```

Methanocella arvoryzae MRE50 (WP_012037364.1)

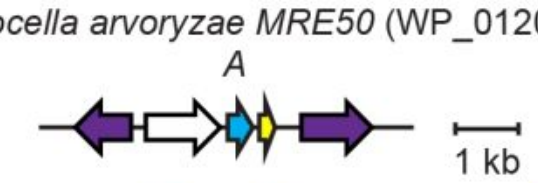


```
A: MAESRRYPLKTDPQGMVGRECPNPSCMTYFKVEASQVDCDELICPVCGRTDD
   CKKYTTVPQIDYINSLIFHKDSCPITHTGYSKTPPCRDYVELPAKCVYSCDS
   CNKKFGIDCKKPENCPYCNATREHLHEENKCDLNCSE
```

Methanosphaera sp. (WP_288975422.1)

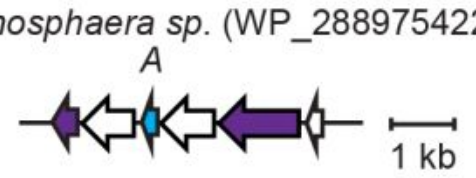


```
A: MSIQIKNDHINGTDSFNILDFNITETGIEEIFTGSNCVPVRQTKSTYLIQDCT
   TIDCTTIQCTTVQCTTVNCTTINCTTIQCNQVQCINCTECTHCSQCNCDCNDN
```

Candidatus Methanoprimaticola sp. MG2 (WP_414596229.1)

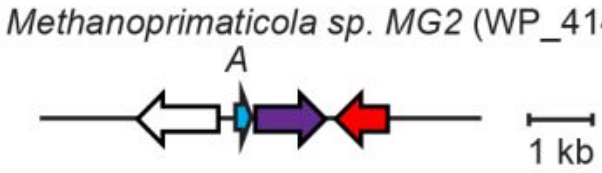


```
A: MAYDAESVDKLTALYESQSPAAFIAFAMEGLPENYDHDGRFDGAADEEHGGD
   SKCSYCCCGDNGYCNSLGWHACVVGLVVCCCACCCYVTNGFNGVWTYWPF
```

Candidatus Bathycorpusculum sp. (WP_343382477.1)

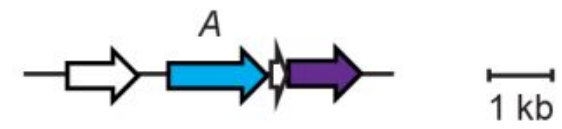


```
A: MSYNIYRTIPQTHPHHTHFGCHTVPPDIGGRGVTTWAHPGGVSYTIAPNQSK
   TISFTCTQPECYVQKDWCNVHFKATPKGTEEDTQLKFAVAGQTQDMWLSGVP
   DHGVWQTYELSAITITAYNDTGLNTIILTNNSSNSTIEISNFRILRAYEMKN
   NYNAICNPAVNTGSYGNLDSSRYDYPCRLNSPSCGERSVSDARDNICHPTNG
   FTIPAGGSVEWEFDWTNHTSTNYLTKDVTLFNFNEIAATTKNDVESDVKLTA
   YINGSLLTTFYLSKKEKHSAFPSYDLTTNVAYNDTGANTVKLVNNSSVPVRL
   WDGYGVNIYRVYKSAGICPPTCGSCVMCYNCYLCYAWYLSCSAGCESSCLDC
   QVGCEVSCYGCEGCQNSCEYACQNCQICEGCQFCVNCEGSELCPGGYYICQQ
   CETCQPYYTPGCEGCQNSCEYACQNCQACEAVCQDCQTCEDCQACISCQGSE
   LCPGDYVCNWCETCQPYQTPKHNPPPTNIPPIKDTSPIKDTSPIKDTSPPKD
   TSPPKDTKLTNQPAQKTETVN
```

Candidatus Bathycorpusculum sp. (WP_343378948.1)

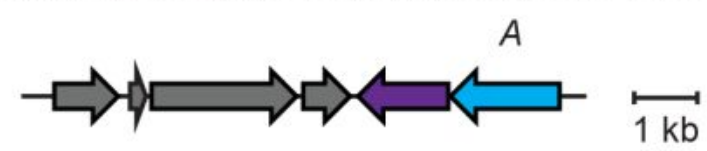


```
A: MSTPKKEYQHYQCDPNWAQTTGYNSQCRGITTWNLNNQYTIPPLAPNGTIEL
   TFTCTVPRCYSSGNTIYGESIQGRDSCIIVYNAYPDKTTPKDEEIELKYRLN
   NGTIDEKLCCISREEKHGGTQAFDLAFIRPATLYNDMGLNTLKITNAGESTV
   HIDHFKIYRIYQMCNIAKVNYDSMCAISGVCEPGTVAGASGNLDSSRIDKPC
   NYSTAGGLSFTQYHDTSHTLTILGPTQTCSWTFNFNNNTINNGYTGNYTGES
   ICLFNFNKIYTCSGTASSLDVAAEAYLNGNKINTYYLSKLPTDLHGMFPSHD
   LATSPYYNAYGPNTVTLKNASSATIQMSENDGIDIYRIFKTGEIYSPPCLGG
   CQTFCQASCQASCQGCQGCQTCYGCQNGCQFFCVTCDACYSFCQACQGGCDA
   TCQACQTTCQYCQNGCQDCQGGCQGCQQSCEVMCQTVCESCASCYQDCYNVC
   YEYTNRNNNPQTNNNTPTTNTTQNNDTTQTPQTNINTTNNQTTTTQPDTNNN
   NTIQQQNPTITINTQPLNQTQTQQQTTPTTNTQNQNHIRQNSNFNVAPITEE
   HIKEILLETLIELGLITQPPTRRQITNDNQ
```

Figure S3: Archaeal BGCs with cysteine-rich protein and RS enzymes. Genome architecture diagrams are shown for RS enzymes associated with cysteine-rich proteins in SSN cluster 3 (**Figure 2A**). Sequences of cysteine-rich protein are shown, and cysteine residues are bolded, underlined, and colored purple. The Crp BGC, which is included in this group, is shown in **Figure 2B**.

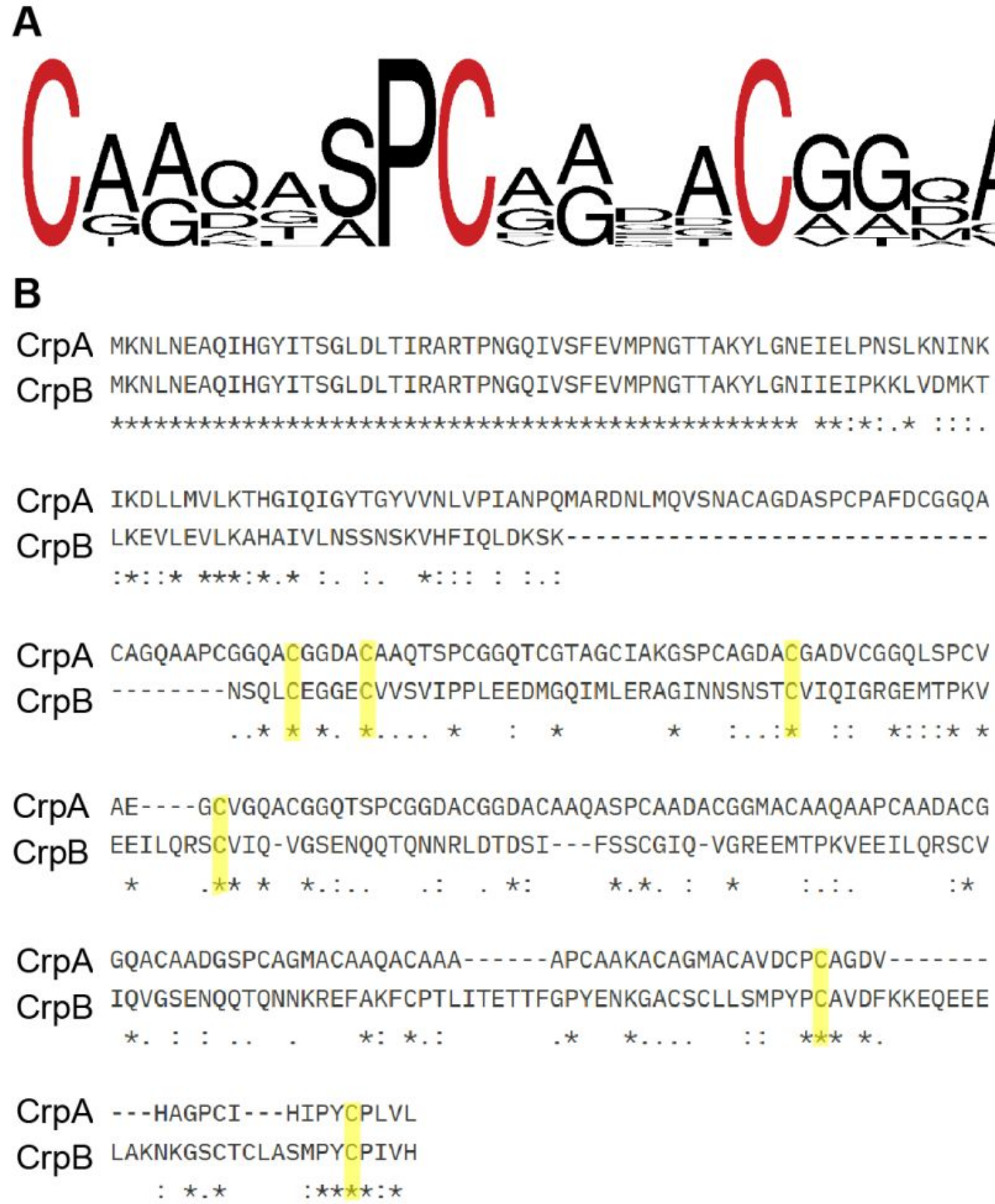

Figure S4: Sequence analysis of CrpA and CrpB. (A) Repeating cysteine motif identified in CrpA, with cysteines shown in red. The 17-reside motif that is repeated 10 times in CrpA was used as the input. The sequence logo was generated using weblogo.[7] (B) Multiple sequence alignment of CrpA and CrpB made with Muscle5.[8] Conserved Cys residues are highlighted.

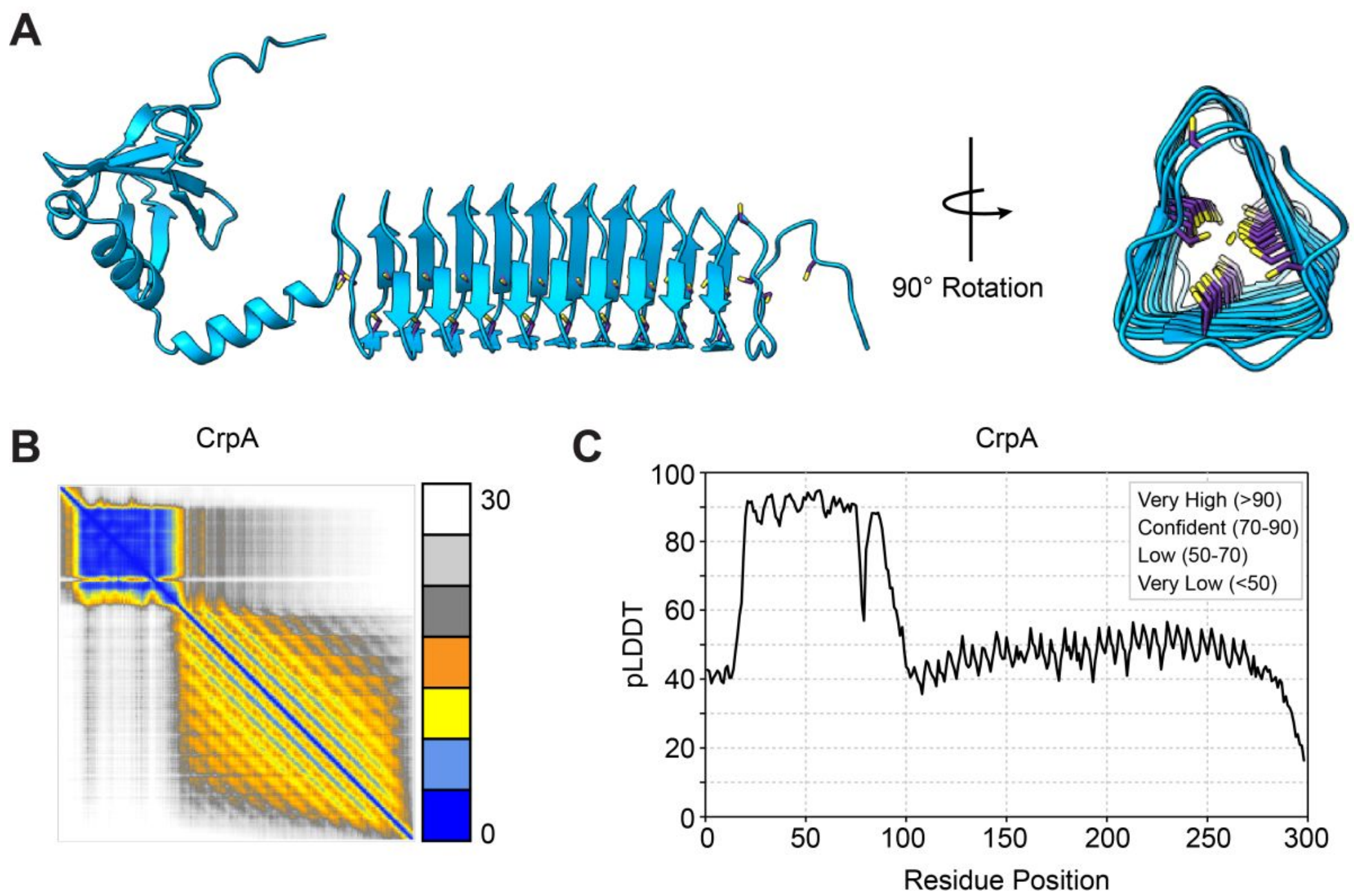

Figure S5: AlphaFold3 predicted structure of CrpA. Predicted structures for (A) CrpA. For the rotated CrpA structure, the N-terminal domain and last 7 residues were omitted for clarity. Cysteine residues are shown in sticks and colored purple (B) Predicted alignment error (PAE) plot for CrpA. (C) Predicted local distance difference test (pLDDT) plots for CrpA. Suggested pLDDT confidence region cutoffs are indicated.

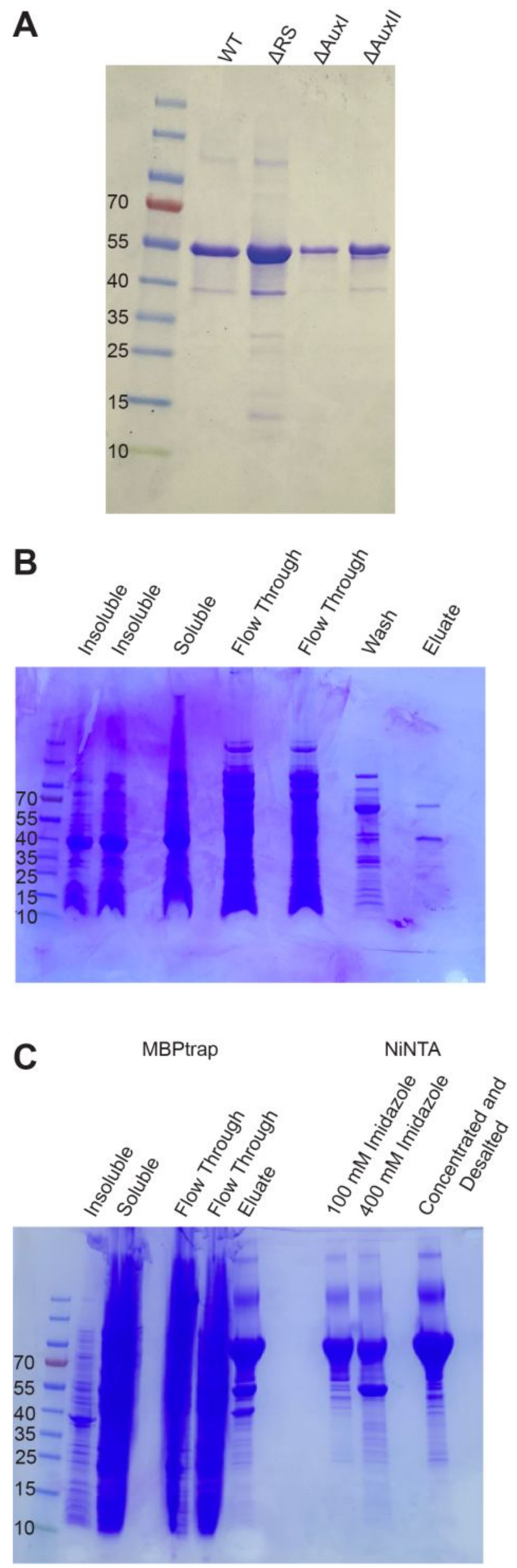

Figure S6: SDS-PAGE of purified CrpC and CrpA. (A) Purified His_{10}-CrpC (54.2 kDa) WT and [4Fe-4S] cluster variants. Protein was purified via Ni-NTA. Protein ladder was loaded into lane 1. The lanes correspond to: WT (wild type CrpC), ΔRS (C104A, C108A, and C111A), ΔAuxI (C333A, C352A, and C403A), ΔAuxII (C390A, C393A, C399A, and C422A). (B) Purified His_{10}-CrpC ΔRRE (43 kDa) purified via Ni-NTA. Protein ladder was loaded into lane 1. (C) Purified His_{6}-MBP-CrpA (73 kDa) from a CrpABC co-expression. Protein ladder was loaded into lane 1. CrpA was first purified via MBPtrap followed by NiNTA (See **Materials and Methods** for full details).

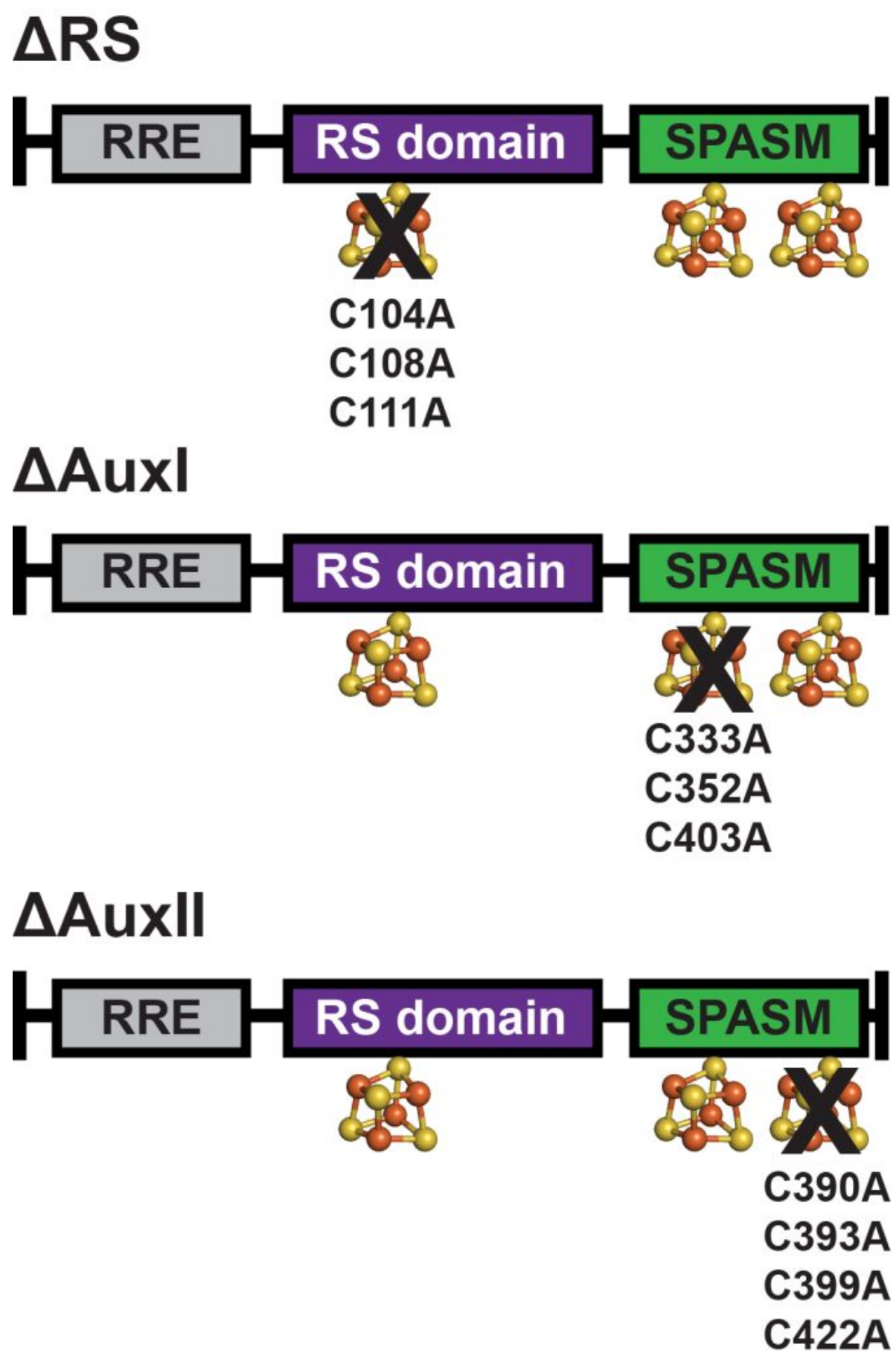

Figure S7: Line diagrams of CrpC [4Fe-4S] cluster variants. The predicted RRE, RS, and SPASM domains are indicated with residues that ligate the Fe-S clusters indicated. Cluster variants contain Cys to Ala substitutions.

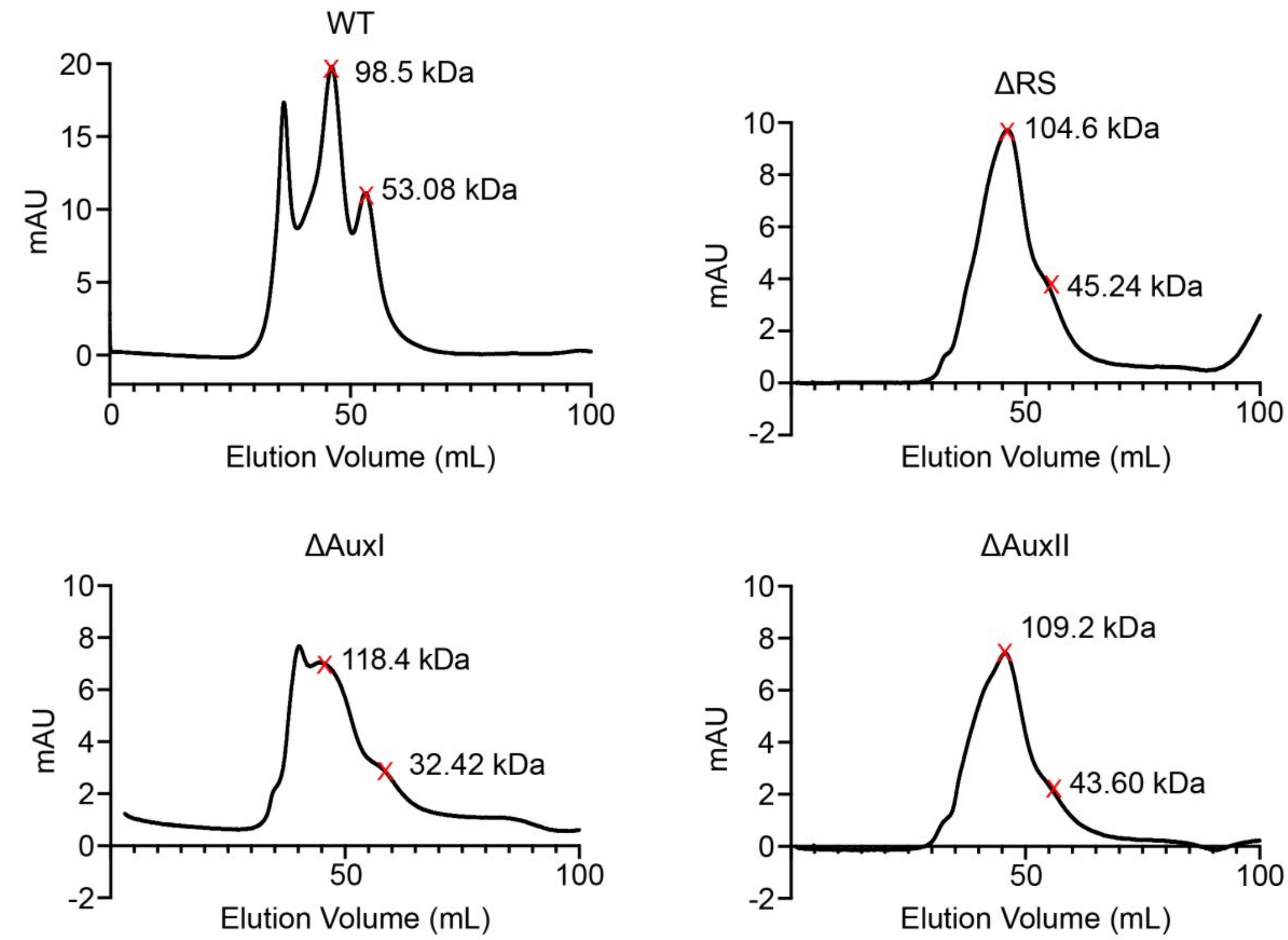

Figure S8: Size exclusion chromatography of CrpC WT and [4Fe-4S] cluster variants. The CrpC protein analyzed is listed above the relevant chromatogram. A standard curve was prepared using gel filtration standards which contained protein standards for 670 kDa (thyroglobulin), 158 kDa (γ-globulin), 44 kDa (ovalalbumin), 17 kDa (myoglobin), and 1.35 kDa (vitamin B_{12}). Injections of 250 μL containing 20 μM protein for each variant were used for analysis. The expected MW for His_{10}-CrpC is 54.2 kDa. SEC was performed on a HiPrep 16/60 Sephacryl S-200 High Resolution column with a flow rate of 0.5 mL/min in 75 mM Tris pH 8, 100 mM KCl, and 5% glycerol buffer.

Table S5: Quantified iron and sulfur content of CrpC constructs. Iron and sulfur content was determined using atomic absorption spectroscopy and Beinert's assay[9], respectively.

	Iron		Sulfur	
**Construct**	**Fe/monomer**	**Std. Dev.**	**S/monomer**	**Std Dev.**
CrpC WT	13.51	0.41	15.39	4.4
CrpC ΔRS cluster	6.47	0.79	8.19	3.02
CrpC ΔAuxI cluster	7.5	0.26	4.64	0.10
CrpC ΔAuxII cluster	6.05	0.34	9.23	1.14

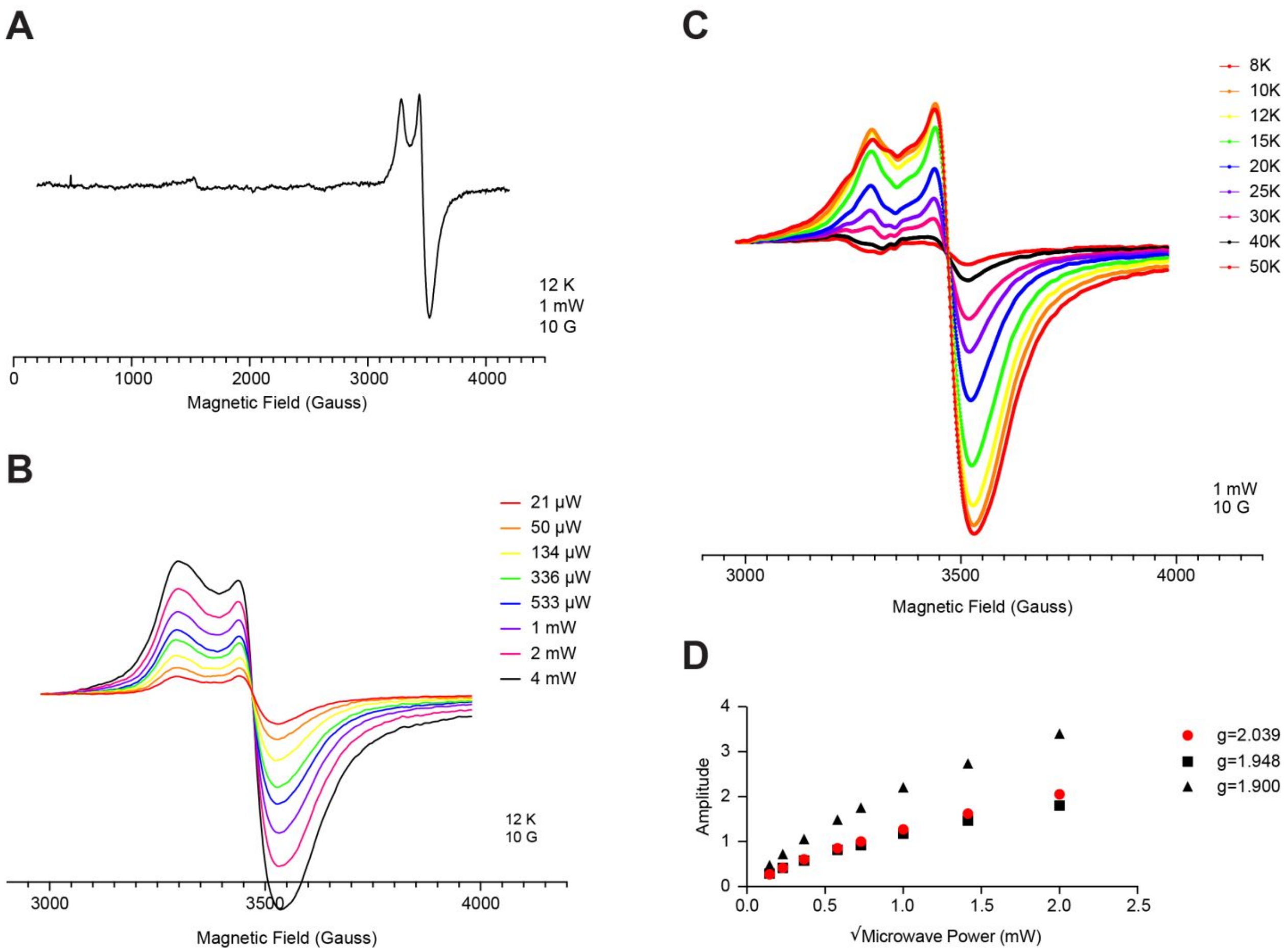

Figure S9: EPR spectroscopic characterization of WT CrpC. (A) Widefield CW X-band EPR spectrum of reconstituted and reduced WT CrpC. (B) Evaluation of power saturation for WT CrpC. (C) Temperature dependence of the EPR signal of WT CrpC. (D) EPR signal amplitude as a function of power for WT CrpC.

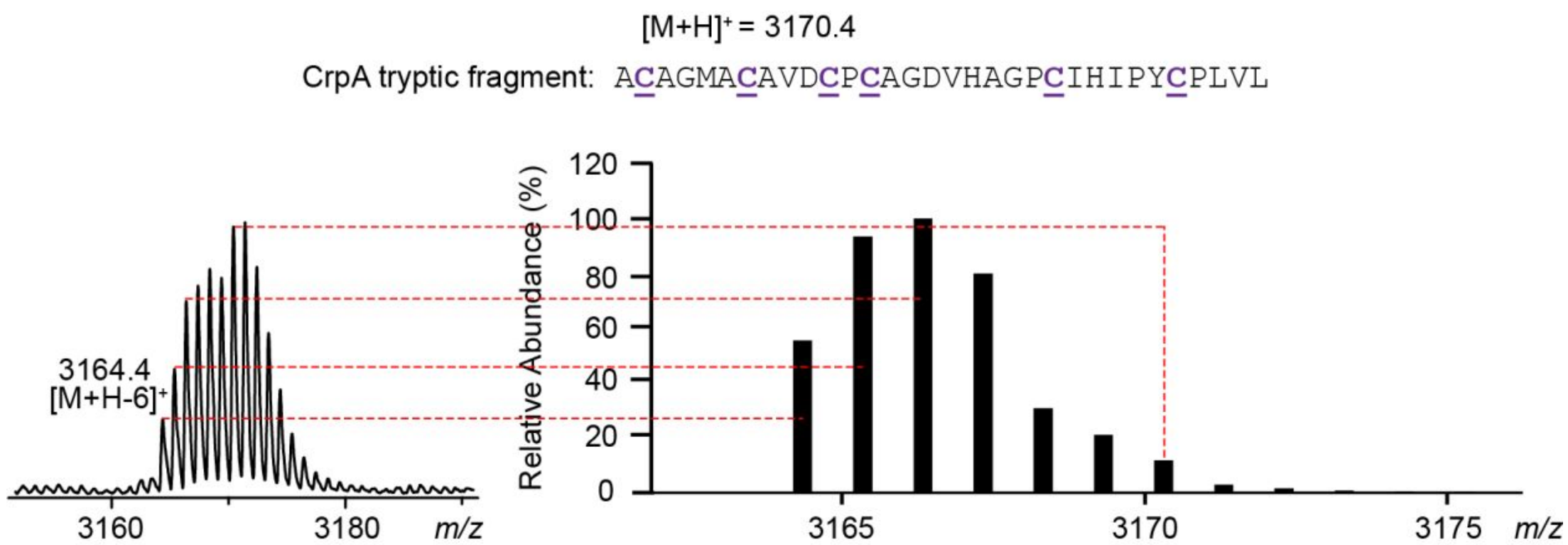

Figure S10: Isotopic distribution analysis of modified CrpA. *Left*: MALDI-TOF-MS spectra of the C-terminal portion of CrpA after co-expression with CrpBC (same as data shown in middle panel of **Figure 4B)**. *Right*: Expected isotopic distribution of a molecule with the same molecular formula. Differences in observed vs calculated relative abundances are shown with red dashed lines. Isotopic distribution was calculated via ChemCalc.[10]

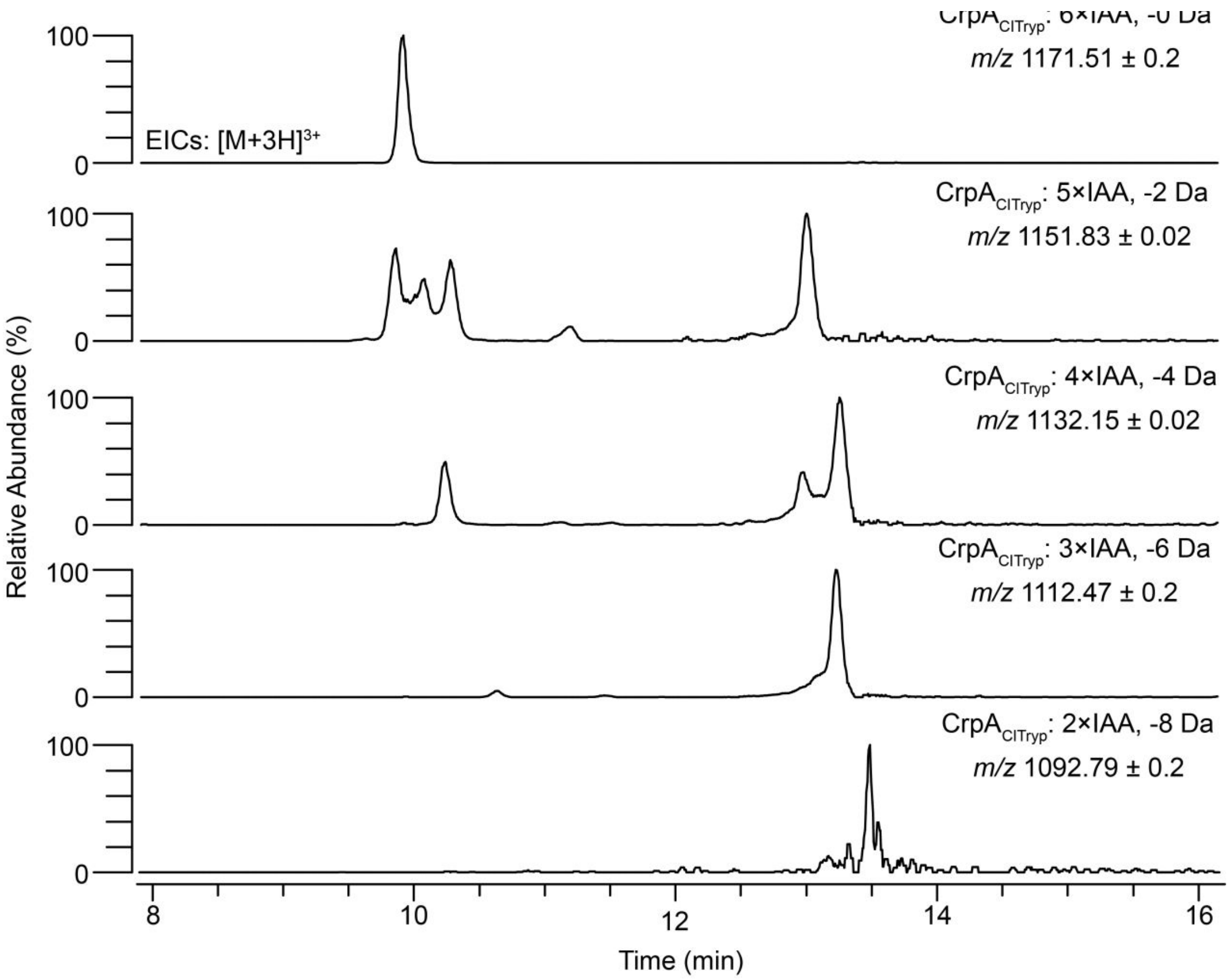

Figure S11: LC-MS chromatograms of IAA-labeled and trypsin-digested CrpA. CrpC-modified CrpA was subjected to IAA alkylation and trypsin digestion followed by LC-MS (see methods for the entire workflow). Extracted ion chromatograms (EICs) are shown. See **Figure 4C** for corresponding MALDI spectrum. MS/MS for each CrpA tryptic fragment is provided in **Figures S12–S20,** and exact masses for observed daughter ions are in **Tables S6-S14**.

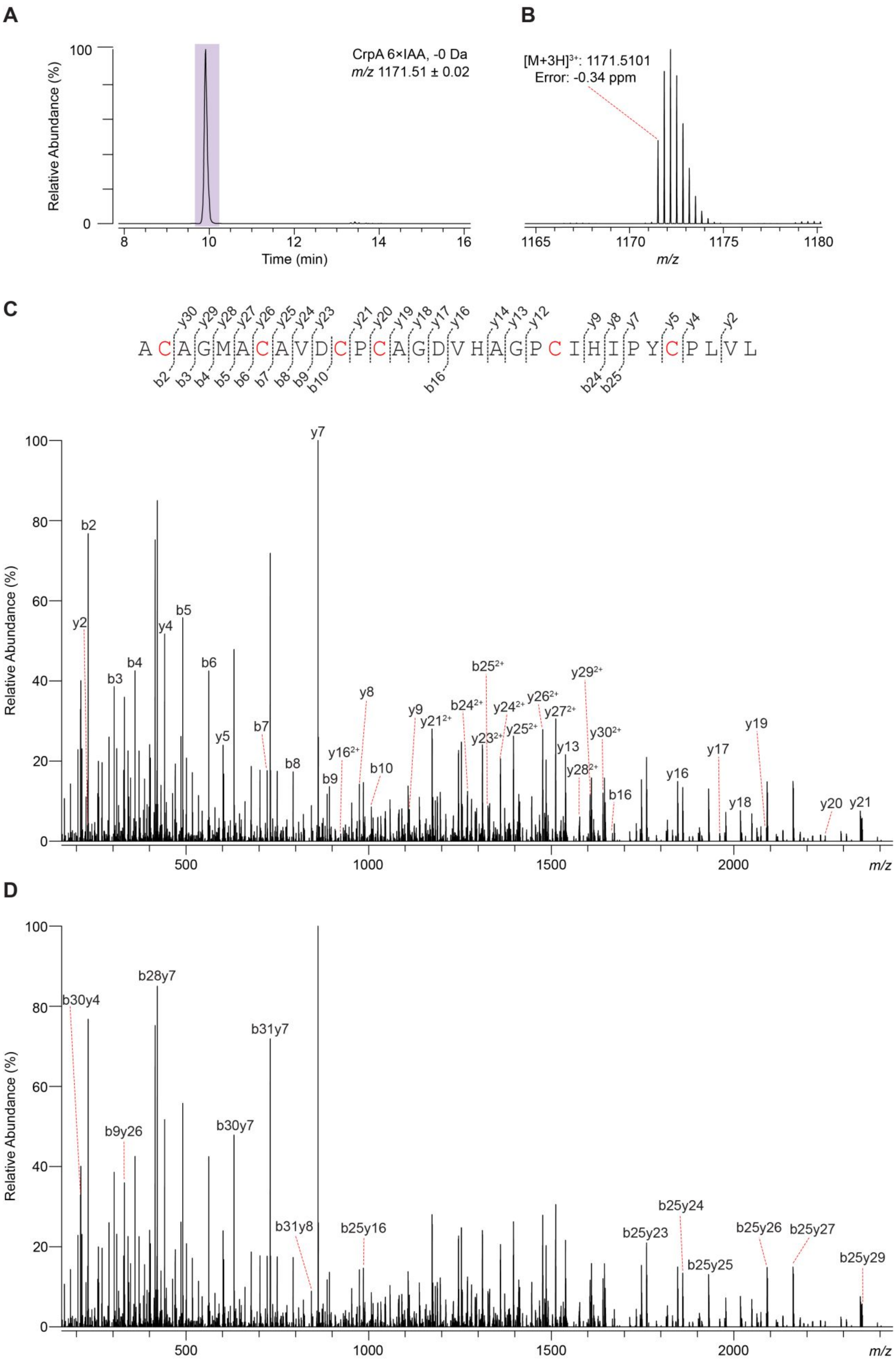
A
B
C
D
CrpA 6×IAA, -0 Da
m/z 1171.51 ± 0.02
[M+3H]3+: 1171.5101
Error: -0.34 ppm
Time (min)
m/z
Relative Abundance (%)
A C A G M A C A V D C P C A G D V H A G P C I H I P Y C P L V L

Figure S12 (previous page): HR-LC-MS/MS analysis of 6×IAA labeled CrpA tryptic fragment. (A) EIC for 6×IAA $CrpA_{CITryp}$, and (B) the HR-MS spectrum of the peptide fragment. The selected molecular ion (*m/z* 1171.51 ± 0.02) in the purple shaded area was fragmented via higher-energy collision induced dissociation (HCD). (C) *Top:* Summary of observed HCD daughter ions. Red cysteine indicates IAA modification. *Bottom:* HCD MS/MS spectrum. All ions are shown in the 1+ charge state unless otherwise stated. (D) An identical spectrum to (C) with key internal daughter ions indicated. Where masses can correspond to multiple possible internal fragments, the most likely one is shown. See **Table S6** for exact masses and errors of all observed daughter ions.

Table S6: Assignment of HCD daughter ions for the 6×IAA-labeled CrpA tryptic fragment. All ions are shown in the 1+ charge state unless otherwise stated. Corresponding HCD spectrum is shown in **Figure S12**. All observed internal ions are included, not just the key ones highlighted in **Figure S12**. Multiple possible internal fragments are listed if they have the same *m/z*.

Ion	Calculated *m/z*	Observed *m/z*	Error (ppm)		Ion	Calculated *m/z*	Observed *m/z*	Error (ppm)
[M+3H]$^{3+}$	1171.5106	1171.5101	-0.43		y9^{+}	1111.5968	1111.5969	0.09
b2^{+}	232.0751	232.0754	1.29		y13^{+}	1538.7859	1538.7859	0.00
b3^{+}	303.1122	303.1125	0.99		y16^{+}	1845.9503	1845.9484	-1.03
b4^{+}	360.1337	360.1340	0.83		y16^{2+}	923.4788	923.4789	0.11
b5^{+}	491.1741	491.1744	0.61		y17^{+}	1960.9773	1960.9826	2.70
b6^{+}	562.2113	562.2113	0.00		y18^{+}	2017.9987	2018.0004	0.84
b7^{+}	722.2419	722.2425	0.83		y19^{+}	2089.0358	2089.0297	-2.92
b8^{+}	793.2790	793.2775	-1.89		y20^{+}	2249.0665	2249.0628	-1.65
b9^{+}	892.3475	892.3481	0.67		y21^{+}	2346.1193	2346.1254	2.60
b10^{+}	1007.3744	1007.3761	1.69		y21^{2+}	1173.5633	1173.5646	1.11
b16^{+}	1667.5741	1667.5706	-2.10		y23^{2+}	1311.0921	1311.0948	2.06
b24^{2+}	1269.9968	1269.9992	1.89		y24^{2+}	1360.6263	1360.6279	1.18
b25^{2+}	1326.5389	1326.5422	2.49		y25^{2+}	1396.1449	1396.1453	0.29
b28^{2+}	1536.6123	1536.6135	0.78		y26^{2+}	1476.1602	1476.1628	1.76
y2^{+}	231.1703	231.1706	1.30		y27^{2+}	1511.6788	1511.6827	2.58
y4^{+}	441.3071	441.3074	0.68		y28^{2+}	1577.1990	1577.1960	-1.90
y5^{+}	601.3378	601.3383	0.83		y29^{2+}	1605.7097	1605.7101	0.25
y7^{+}	861.4539	861.4554	1.74		y30^{2+}	1641.2283	1641.2253	-1.83
y8^{+}	974.5380	974.5380	0.00					

Ion	Calculated *m/z*	Observed *m/z*	Error (ppm)		Ion	Calculated *m/z*	Observed *m/z*	Error (ppm)
b30y4	211.1441	211.1442	0.47		b23y12	371.1748	371.1752	1.08
b5y30	260.1063	260.1067	1.54		b30y5	371.1748	371.1752	1.08
b6y29	260.1063	260.1067	1.54		b15y21	386.1493	386.1495	0.52
b4y31	289.0965	289.0969	1.38		b22y14	386.1493	386.1495	0.52
b15y20	289.0965	289.0969	1.38		b9y27	402.1806	402.1810	0.99
b31y4	310.2125	310.2128	0.97		b5y31	420.1370	420.1373	0.71
b22y13	315.1122	315.1124	0.63		b7y29	420.1370	420.1373	0.71
b14y21	329.1278	329.1282	1.22		b28y7	421.1541	421.1546	1.19
b9y26	331.1435	331.1438	0.91		b29y6	421.1541	421.1546	1.19
b7y28	363.1156	363.116	1.10		b8y28	434.1527	434.1528	0.23
b31y5	470.2432	470.2435	0.64		b10y31	936.3373	936.3366	-0.75

Table S6 (Cont.)

b14y22	489.1585	489.1587	0.41		b11y30	936.3373	936.3366	-0.75
b16y21	501.1762	501.1768	1.20		b16y25	946.3394	946.3378	-1.69
b29y7	518.2068	518.2074	1.16		b19y22	968.3714	968.3733	1.96
b24y13	565.2552	565.2558	1.06		b25y16	985.5037	985.5044	0.71
b9y29	590.2426	590.2443	2.88		b19y23	1083.3983	1083.3998	1.38
b17y21	600.2447	600.2445	-0.33		b28y13	1098.4860	1098.4876	1.46
b11y26	606.2011	606.2024	2.14		b16y26	1106.3701	1106.3677	-2.17
b25y12	621.3178	621.3191	2.09		b25y18	1157.5521	1157.5548	2.33
b26y11	621.3178	621.3191	2.09		b25y19	1228.5892	1228.5891	-0.08
b29y8	631.2909	631.2905	-0.63		b24y21	1372.5886	1372.5901	1.09
b30y7	631.2909	631.2905	-0.63		b18y27	1413.5345	1413.5331	-0.99
b23y15	636.2923	636.2929	0.94		b19y26	1413.5345	1413.5331	-0.99
b24y14	636.2923	636.2929	0.94		b31y14	1478.7284	1478.7295	0.74
b15y23	661.2069	661.2080	1.66		b19y27	1484.5716	1484.5746	2.02
b16y22	661.2069	661.2080	1.66		b24y22	1532.6193	1532.6198	0.33
b9y30	661.2797	661.2805	1.21		b25y22	1645.7033	1645.7025	-0.49
b25y13	678.3392	678.3402	1.47		b31y16	1714.8557	1714.8525	-1.87
b31y7	730.3593	730.3602	1.23		b25y23	1760.7303	1760.7309	0.34
b15y21	737.3036	737.3049	1.76		b24y25	1817.7517	1817.7475	-2.31
b22y17	737.3036	737.3049	1.76		b25y24	1859.7987	1859.7992	0.27
b15y24	760.2753	760.2775	2.89		b27y22	1905.8194	1905.8184	-0.52
b17y22	760.2753	760.2775	2.89		b28y21	1905.8194	1905.8184	-0.52
b9y31	821.3104	821.3112	0.97		b29y20	1905.8194	1905.8184	-0.52
b31y8	843.4434	843.4453	2.25		b25y25	1930.8358	1930.8357	-0.05
b16y24	875.3023	875.3001	-2.51		b24y26	1977.7824	1977.7799	-1.26
b17y23	875.3023	875.3001	-2.51		b24y27	2048.8195	2048.8172	-1.12
b25y15	886.4353	886.4355	0.23		b25y26	2090.8665	2090.8616	-2.34
b18y22	897.3343	897.3342	-0.11		b25y27	2161.9036	2161.9032	-0.19
b13y27	934.3217	934.3223	0.64		b25y28	2292.9441	2292.9414	-1.18
b14y26	934.3217	934.3223	0.64		b25y29	2349.9655	2349.9683	1.19

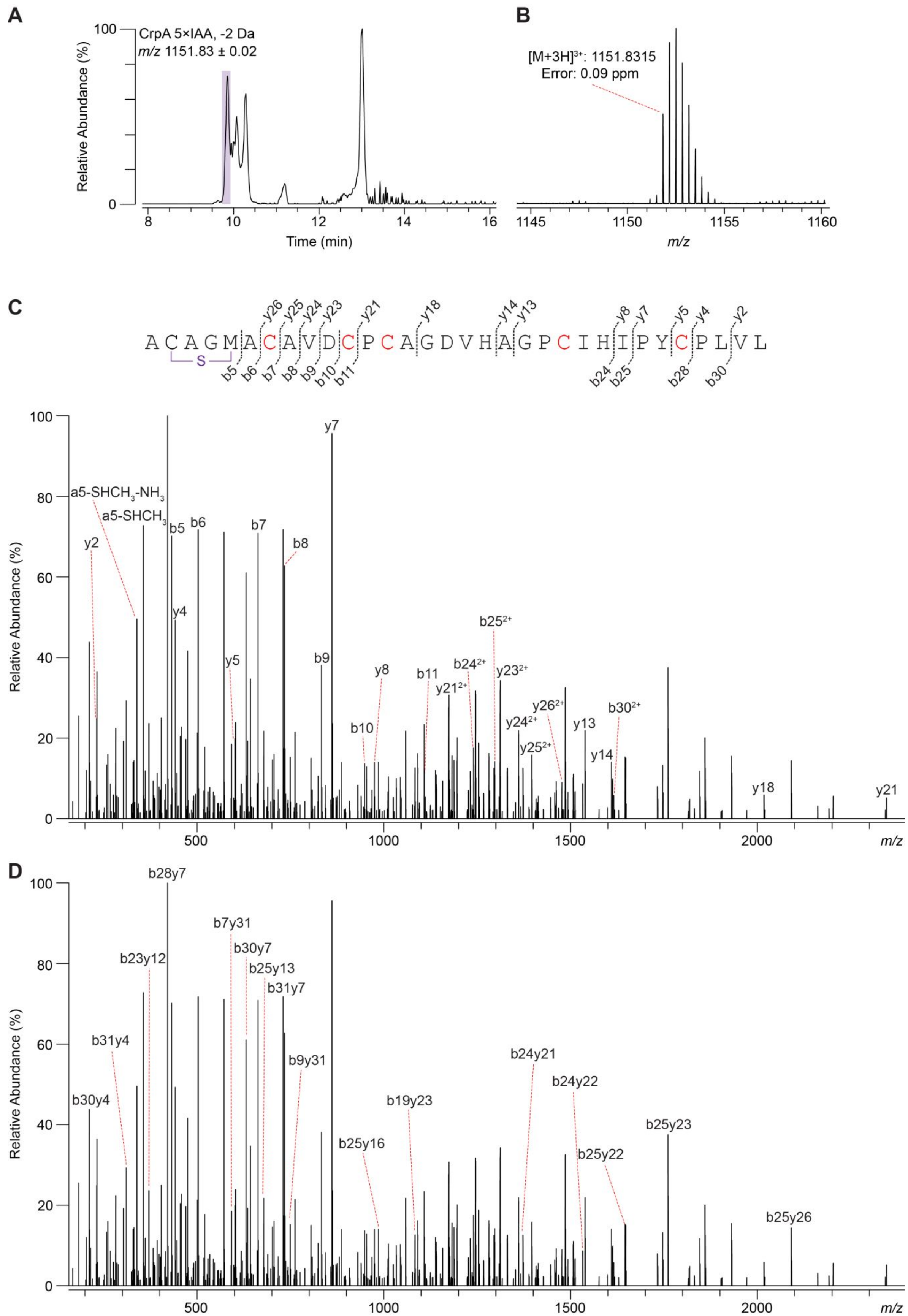
A
CrpA 5×IAA, -2 Da
m/z 1151.83 ± 0.02
Relative Abundance (%)
Time (min)
B
[M+3H]3+: 1151.8315
Error: 0.09 ppm
m/z
C
ACAGMACAVDCPCAGDVHAGPCIHIPYCPLVL
S
y26 y25 y24 y23 y21 y18 y14 y13 y8 y7 y5 y4 y2
b5 b6 b7 b8 b9 b10 b11 b24 b25 b28 b30
a5-SHCH3-NH3
a5-SHCH3
y2 b5 b6 b7 b8 y4 y5 b9 y8 b11 b10 b25 2+ b24 2+ y21 2+ y23 2+ y24 2+ y26 2+ y25 2+ y13 y14 b30 2+ y18 y21 y7
D
b28y7 b7y31 b30y7 b25y13 b31y7 b23y12 b31y4 b30y4 b9y31 b24y21 b24y22 b19y23 b25y16 b25y22 b25y23 b25y26

Figure S13 (previous page): HR-LC-MS/MS analysis of 5×IAA labeled CrpA^{C2}-modified tryptic fragment. (A) EIC for 5×IAA CrpA$_{CITryp}$ where Cys2 is modified, and (B) the HR-MS spectrum of the peptide fragment. The selected molecular ion (*m/z* 1151.83 ± 0.02) in the purple shaded area was fragmented via higher-energy collision induced dissociation (HCD). (C) *Top:* Summary of observed HCD daughter ions. Red cysteine indicates IAA modification. Hypothesized thioether bond is indicated in purple. *Bottom:* HCD MS/MS spectrum. All ions are shown in the 1+ charge state unless otherwise stated. The a5 ion (ACAGM) was observed with losses of $SHCH_3$ and NH_3 from methionine, neutral losses with precedence in the proteomics literature.[11,12] (D) An identical spectrum to (C) with key internal daughter ions indicated. Where masses can correspond to multiple possible internal fragments, the most likely one is shown. See **Table S7** for exact masses and errors of all observed daughter ions.

Table S7: Assignment of HCD daughter ions for the 5×IAA-labeled CrpA^{C2}-modified tryptic fragment. All ions are shown in the 1+ charge state unless otherwise stated. Corresponding HCD spectrum is highlighted in **Figure S13**. All observed internal ions are included, not just the key ones shown in **Figure S13**. Multiple possible internal fragments are listed if they have the same *m/z*.

Ion	Calculated *m/z*	Observed *m/z*	Error (ppm)		Ion	Calculated *m/z*	Observed *m/z*	Error (ppm)
$[M+3H]^{3+}$	1151.8314	1151.8315	0.09		$y4^{+}$	441.3071	441.3075	0.91
$b5^{+}$	432.1370	432.1373	0.69		$y5^{+}$	601.3378	601.3388	1.66
a5-$SHCH_3$	356.1387	356.1392	1.40		$y7^{+}$	861.4539	861.4555	1.86
a5-$SHCH_3$-NH_3	339.1122	339.1127	1.47		$y8^{+}$	974.5380	974.5369	-1.13
$b6^{+}$	503.1742	503.1745	0.60		$y13^{+}$	1538.7859	1538.7889	1.95
$b7^{+}$	663.2048	663.2054	0.90		$y14^{+}$	1609.8230	1609.8273	2.67
$b8^{+}$	734.2419	734.2418	-0.14		$y18^{+}$	2017.9987	2017.9966	-1.04
$b9^{+}$	833.3104	833.3103	-0.12		$y21^{+}$	2346.1193	2346.1172	-0.90
$b10^{+}$	948.3373	948.3389	1.69		$y21^{2+}$	1173.5633	1173.5674	3.49
$b11^{+}$	1108.3680	1108.3669	-0.99		$y23^{2+}$	1311.0921	1311.0957	2.75
$b24^{2+}$	1240.4783	1240.4785	0.16		$y24^{2+}$	1360.6263	1360.6280	1.25
$b25^{2+}$	1297.0203	1297.0227	1.85		$y25^{2+}$	1396.1449	1396.1491	3.01
$b30^{2+}$	1612.1621	1612.1662	2.54		$y26^{2+}$	1476.1573	1476.1602	1.96
$y2^{+}$	231.1703	231.1708	2.16					

Ion	Calculated *m/z*	Observed *m/z*	Error (ppm)		Ion	Calculated *m/z*	Observed *m/z*	Error (ppm)
b30y4	211.1441	211.1443	0.95		b7y31	592.1677	592.1685	1.35
b4y31	289.0965	289.0962	-1.04		b25y12	621.3178	621.3184	0.97
b31y4	310.2125	310.2129	1.29		b26y11	621.3178	621.3184	0.97
b14y21	329.1278	329.1277	-0.30		b29y8	631.2909	631.2921	1.90
b7y28	361.1000	361.1004	1.11		b30y7	631.2909	631.2921	1.90
b23y12	371.1748	371.1752	1.08		b25y13	678.3392	678.3411	2.80
b30y5	371.1748	371.1752	1.08		b31y7	730.3593	730.3602	1.23
b9y27	402.1806	402.1807	0.25		b15y21	737.3036	737.3044	1.09
b28y7	421.1541	421.1546	1.19		b22y17	737.3036	737.3044	1.09
b29y6	421.1541	421.1546	1.19		b25y14	749.3763	749.3771	1.07
b10y26	446.1704	446.1701	-0.67		b9y31	762.2733	762.2750	2.23
b11y25	446.1704	446.1701	-0.67		b31y8	843.4434	843.4415	-2.25
b31y5	470.2432	470.2430	-0.43		b16y24	875.3023	875.3048	2.86
b29y7	518.2068	518.2053	-2.89		b17y23	875.3023	875.3048	2.86
b28y8	534.2381	534.2381	0.00		b19y22	968.3714	968.3688	-2.68
b30y6	534.2381	534.2381	0.00		b25y16	985.5037	985.5010	-2.74

Table S7 (Cont.)

b18y23	1012.3612	1012.3590	-2.17		b30y16	1615.7873	1615.7832	-2.54
b19y23	1083.3983	1083.3968	-1.38		b31y15	1615.7873	1615.7832	-2.54
b30y13	1308.6228	1308.6222	-0.46		b25y22	1645.7033	1645.7057	1.46
b24y21	1372.5886	1372.5904	1.31		b25y24	1647.6462	1647.6500	2.31
b18y27	1413.5345	1413.5348	0.21		b25y23	1760.7303	1760.7323	1.14
b19y26	1413.5345	1413.5348	0.21		b25y26	2090.8665	2090.8611	-2.58
b24y22	1532.6193	1532.6169	-1.57					

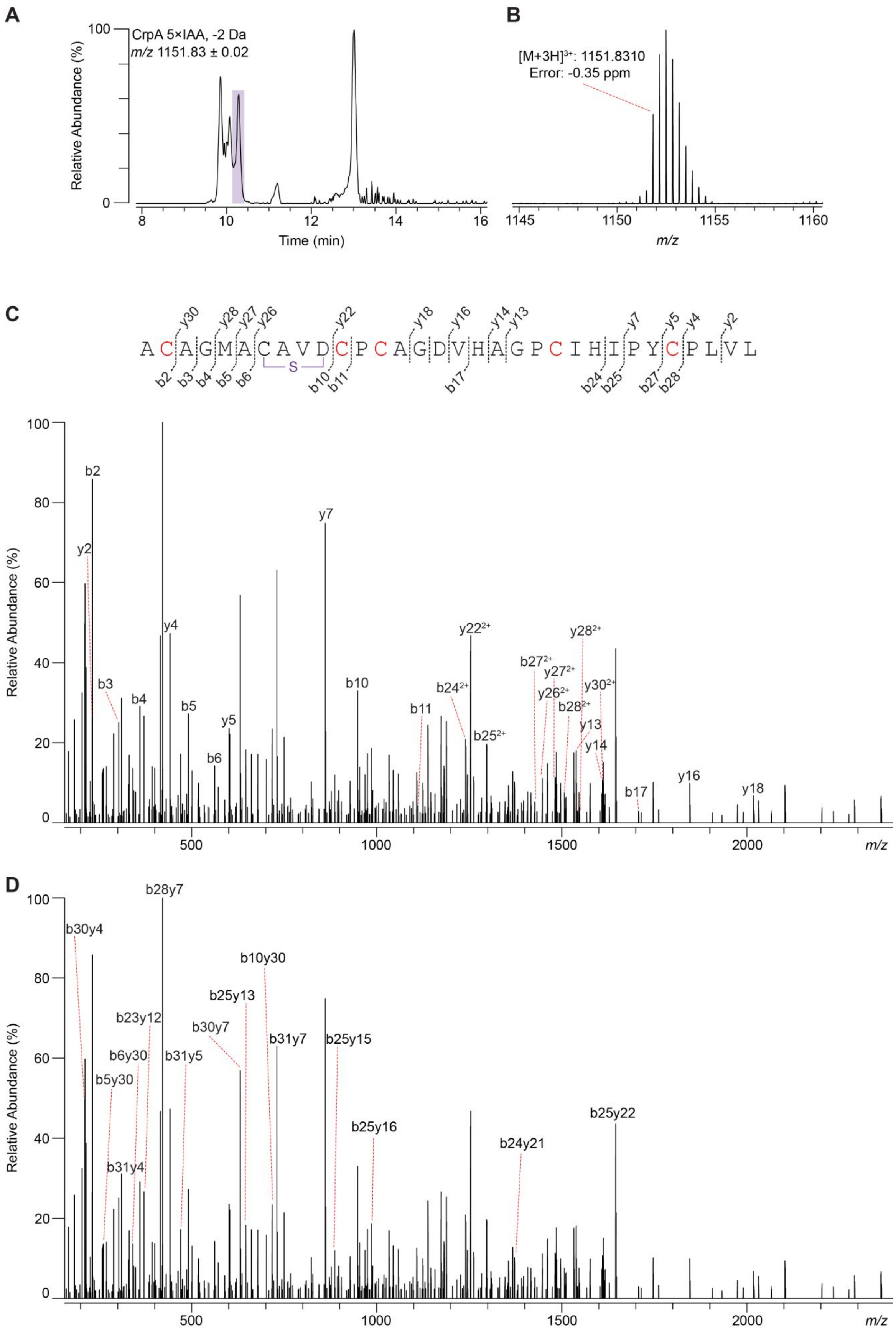
A
CrpA 5×IAA, -2 Da
m/z 1151.83 ± 0.02
Relative Abundance (%)
Time (min)
B
[M+3H]3+: 1151.8310
Error: -0.35 ppm
m/z
C
A C A G M A C A V D C P C A G D V H A G P C I H I P Y C P L V L
S
y30 y28 y27 y26 y22 y18 y16 y14 y13 y7 y5 y4 y2
b2 b3 b4 b5 b6 b10 b11 b17 b24 b25 b27 b28
Relative Abundance (%)
b2
y2
y7
y4
b3
b4
b5
y5
b6
b10
b11
b24 2+
y22 2+
b25 2+
b27 2+
y27 2+
y26 2+
b28 2+
y28 2+
y30 2+
y13
y14
b17
y16
y18
m/z
D
Relative Abundance (%)
b28y7
b30y4
b10y30
b25y13
b23y12
b30y7
b31y7
b25y15
b6y30
b31y5
b5y30
b31y4
b25y16
b24y21
b25y22
m/z

Figure S14 (previous page): HR-LC-MS/MS analysis of 5×IAA labeled CrpA^{C7}-modified tryptic fragment. (A) EIC for 5×IAA CrpA$_{CITryp}$ where Cys7 is modified, and (B) the HR-MS spectrum of the peptide fragment. Selected molecular ions (*m/z* 1151.83 ± 0.02) in the purple shaded area were fragmented via higher-energy collision induced dissociation (HCD). (C) *Top:* Summary of observed HCD daughter ions. Red cysteine indicates IAA modification. Hypothesized thioether bond is indicated in purple. *Bottom:* HCD MS/MS spectrum. All ions are shown in the 1+ charge state unless otherwise stated. (D) An identical spectrum to (C) with key internal daughter ions. Where masses can correspond to multiple possible internal fragments, the most likely one is shown. See **Table S8** for exact masses and errors of all observed daughter ions.

Table S8: Assignment of HCD daughter ions for the 5×IAA-labeled CrpA^{C7}-modified tryptic fragment. All ions are shown in the 1+ charge state unless otherwise stated. Corresponding HCD spectrum is highlighted in **Figure S14**. All observed internal ions are included, not just the key ones shown in **Figure S14**. Multiple possible internal fragments are listed if they have the same *m/z*.

Ion	Calculated *m/z*	Observed *m/z*	Error (ppm)		Ion	Calculated *m/z*	Observed *m/z*	Error (ppm)
$[M+3H]^{3+}$	1151.8314	1151.8310	-0.35		$y2^{+}$	231.1703	231.1709	2.60
$b2^{+}$	232.0751	232.0755	1.72		$y4^{+}$	441.3071	441.3076	1.13
$b3^{+}$	303.1122	303.1125	0.99		$y5^{+}$	601.3378	601.3385	1.16
$b4^{+}$	360.1337	360.1341	1.11		$y7^{+}$	861.4539	861.4549	1.16
$b5^{+}$	491.1741	491.1740	-0.20		$y13^{+}$	1538.7859	1538.7902	2.79
$b6^{+}$	562.2113	562.2111	-0.36		$y14^{+}$	1609.8230	1609.8229	-0.06
$b10^{+}$	948.3373	948.3394	2.21		$y16^{+}$	1845.9503	1845.9502	-0.05
$b11^{+}$	1108.3680	1108.3685	0.45		$y18^{+}$	2017.9987	2017.9971	-0.79
$b17^{+}$	1707.6054	1707.6082	1.64		$y22^{2+}$	1253.5786	1253.5829	3.43
$b24^{2+}$	1240.4783	1240.4766	-1.37		$y26^{2+}$	1446.6417	1446.6370	-3.25
$b25^{2+}$	1297.0203	1297.0216	1.00		$y27^{2+}$	1482.1602	1482.1619	1.15
$b27^{2+}$	1427.0784	1427.0819	2.45		$y28^{2+}$	1547.6804	1547.6768	-2.33
$b28^{2+}$	1507.0937	1507.0909	-1.86		$y30^{2+}$	1611.7097	1611.7047	-3.10

Ion	Calculated *m/z*	Observed *m/z*	Error (ppm)		Ion	Calculated *m/z*	Observed *m/z*	Error (ppm)
b30y4	211.1441	211.1442	0.47		b29y8	631.2909	631.2917	1.27
b5y30	260.1063	260.1069	2.31		b30y7	631.2909	631.2917	1.27
b6y29	260.1063	260.1069	2.31		b23y15	636.2923	636.2925	0.31
b4y31	289.0965	289.0969	1.38		b24y14	636.2923	636.2925	0.31
b15y20	289.0965	289.0969	1.38		b10y29	646.2324	646.2335	1.70
b30y4	310.2125	310.2130	1.61		b25y13	678.3392	678.3398	0.88
b14y21	329.1278	329.1283	1.52		b10y30	717.2695	717.2710	2.09
b6y30	331.1435	331.1443	2.42		b31y7	730.3593	730.3605	1.64
b23y12	371.1748	371.1752	1.08		b10y31	877.3002	877.3013	1.25
b30y5	371.1748	371.1752	1.08		b11y30	877.3002	877.3013	1.25
b15y21	386.1493	386.1501	2.07		b25y15	886.4353	886.4361	0.90
b22y14	386.1493	386.1501	2.07		b25y16	985.5037	985.5060	2.33
b5y31	420.1370	420.1372	0.48		b24y21	1372.5886	1372.5859	-1.97
b28y7	421.1541	421.1547	1.42		b31y13	1407.6912	1407.6916	0.28
b29y6	421.1541	421.1547	1.42		b20y28	1613.5965	1613.5956	-0.56
b31y5	470.2432	470.2442	2.13		b18y30	1613.5965	1613.5956	-0.56
b29y7	518.2068	518.2061	-1.35		b19y29	1613.5965	1613.5956	-0.56
b10y28	589.2109	589.2109	0.00		b25y22	1645.7033	1645.7054	1.28

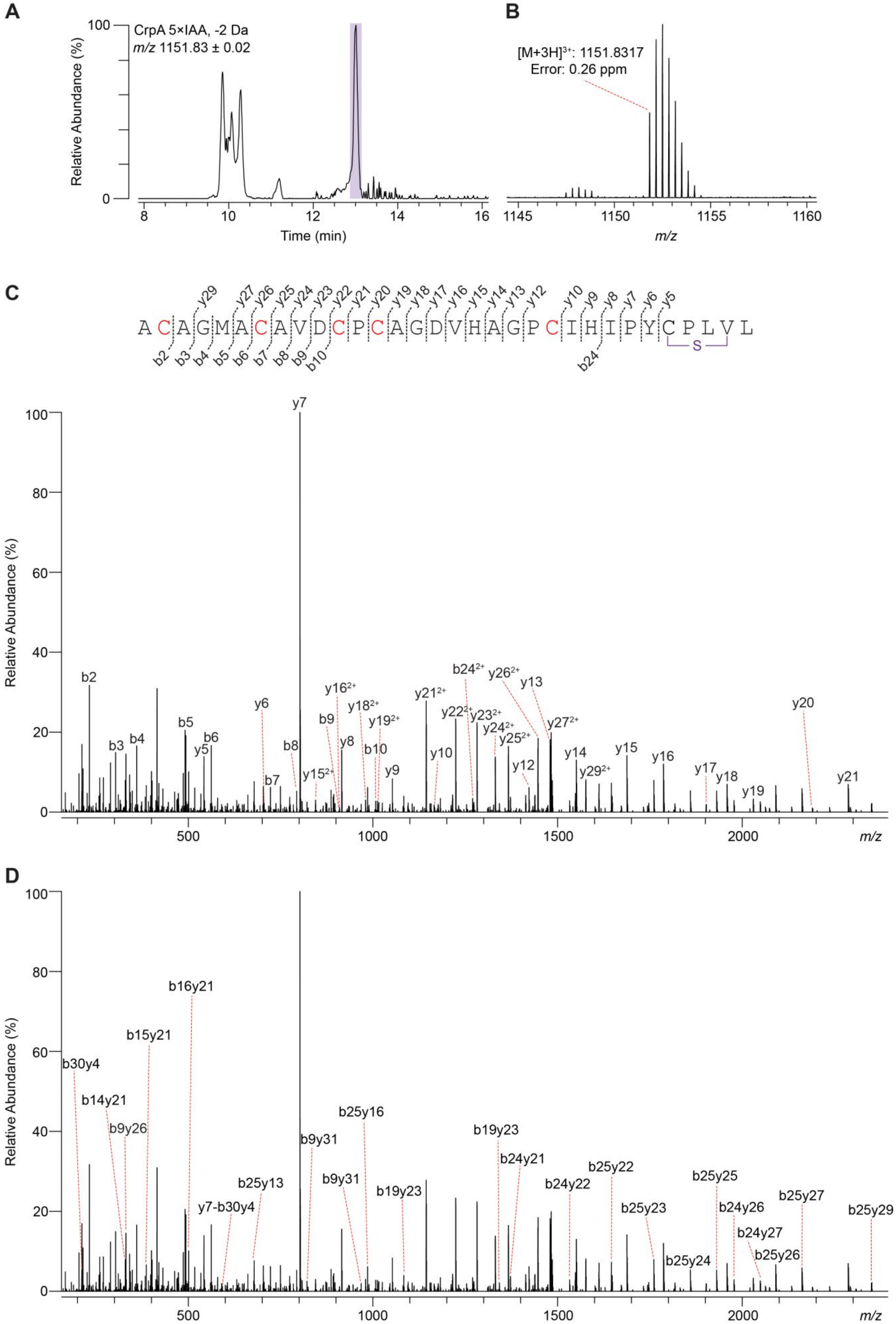
A
CrpA 5×IAA, -2 Da
m/z 1151.83 ± 0.02
Relative Abundance (%)
Time (min)
B
[M+3H]3+: 1151.8317
Error: 0.26 ppm
m/z
C
A C A G M A C A V D C P C A G D V H A G P C I H I P Y C P L V L
S
y29 y27 y26 y25 y24 y23 y22 y21 y20 y19 y18 y17 y16 y15 y14 y13 y12 y10 y9 y8 y7 y6 y5
b2 b3 b4 b5 b6 b7 b8 b9 b10 b24
Relative Abundance (%)
m/z
D
b30y4
b14y21
b9y26
b15y21
b16y21
y7-b30y4
b25y13
b9y31
b25y16
b19y23
b24y21
b24y22
b25y22
b25y23
b25y24
b25y25
b24y26
b24y27
b25y26
b25y27
b25y29
Relative Abundance (%)
m/z

Figure S15 (previous page): HR-LC-MS/MS analysis of 5×IAA labeled CrpA^{C28} -modified tryptic fragment. (A) EIC for 5×IAA CrpA$_{CITryp}$ where Cys28 is modified, and (B) the HR-MS spectrum of the peptide fragment. The selected molecular ion (*m/z* 1151.83 ± 0.02) in the purple shaded area were fragmented via higher-energy collision induced dissociation (HCD). (C) *Top:* Summary of observed HCD daughter ions. Red cysteine indicates IAA modification. Hypothesized thioether bond is indicated in purple. *Bottom:* HCD MS/MS spectrum. All ions are shown in the 1+ charge state unless otherwise stated. (D) An identical spectrum to (C) with key internal daughter ions indicated. Where masses can correspond to multiple possible internal fragments, the most likely one is shown. See **Table S9** for exact masses and errors of all observed daughter ions.

Table S9: Assignment of HCD daughter ions for the 5×IAA-labeled CrpA^{C28}-modified tryptic fragment. All ions are shown in the 1+ charge state unless otherwise stated. Corresponding HCD spectrum is highlighted in **Figure S15**. All observed internal ions are included, not just the key ones shown in **Figure S15**. Multiple possible internal fragments are listed where if have the same *m/z*.

Ion	Calculated *m/z*	Observed *m/z*	Error (ppm)		Ion	Calculated *m/z*	Observed *m/z*	Error (ppm)
$[M+3H]^{3+}$	1151.8314	1151.8317	0.26		$y15^{+}$	1687.8448	1687.8488	2.37
$b2^{+}$	232.0751	232.0755	1.72		$y15^{2+}$	844.4260	844.4275	1.78
$b3^{+}$	303.1122	303.1126	1.32		$y16^{+}$	1786.9132	1786.9154	1.23
$b4^{+}$	360.1337	360.1342	1.39		$y16^{2+}$	893.9602	893.9622	2.24
$b5^{+}$	491.1741	491.1745	0.81		$y17^{+}$	1901.9402	1901.9378	-1.26
$b6^{+}$	562.2113	562.2118	0.89		$y18^{+}$	1958.9616	1958.9666	2.55
$b7^{+}$	722.2419	722.2429	1.38		$y18^{2+}$	979.9844	979.9842	-0.20
$b8^{+}$	793.2790	793.2772	-2.27		$y19^{+}$	2029.9987	2029.9920	-3.30
$b9^{+}$	892.3475	892.3475	0.00		$y19^{2+}$	1015.5030	1015.5054	2.36
$b10^{+}$	1007.3744	1007.3732	-1.19		$y20^{+}$	2190.0294	2190.0227	-3.06
$b24^{2+}$	1269.9968	1269.9943	-1.97		$y21^{+}$	2287.0822	2287.0853	1.36
$y5^{+}$	542.3007	542.3013	1.11		$y21^{2+}$	1144.0447	1144.0458	0.96
$y6^{+}$	705.3641	705.3654	1.84		$y22^{2+}$	1224.0601	1224.0629	2.29
$y7^{+}$	802.4168	802.4181	1.62		$y23^{2+}$	1281.5735	1281.5773	2.97
$y8^{+}$	915.5009	915.5032	2.51		$y24^{2+}$	1331.1078	1331.1090	0.90
$y9^{+}$	1052.5598	1052.5600	0.19		$y25^{2+}$	1366.6263	1366.6298	2.56
$y10^{+}$	1165.6439	1165.6422	-1.46		$y26^{2+}$	1446.6417	1446.6442	1.73
$y12^{+}$	1422.7273	1422.7293	1.41		$y27^{2+}$	1482.1602	1482.1620	1.21
$y13^{+}$	1479.7488	1479.7503	1.01		$y29^{2+}$	1576.1912	1576.1928	1.02
$y14^{+}$	1550.7859	1550.7903	2.84					

Ion	Calculated *m/z*	Observed *m/z*	Error (ppm)		Ion	Calculated *m/z*	Observed *m/z*	Error (ppm)
b30y4	211.1441	211.1443	0.95		b5y31	420.1370	420.1375	1.19
b4y31	289.0965	289.0970	1.73		b7y29	420.1370	420.1375	1.19
b15y20	289.0965	289.0970	1.73		b14y22	489.1585	489.1589	0.82
b22y13	315.1122	315.1129	2.22		b16y21	501.1762	501.1768	1.20
b14y21	329.1278	329.1283	1.52		b24y13	565.2552	565.2565	2.30
b9y26	331.1435	331.1440	1.51		b9y29	590.2426	590.2439	2.20
b7y28	363.1156	363.1155	-0.28		y7-b30y4	592.2800	592.2818	3.04
b15y21	386.1493	386.1496	0.78		b17y21	600.2447	600.2454	1.17
b22y14	386.1493	386.1496	0.78		b14y23	604.1855	604.1869	2.32
b9y27	402.1806	402.1814	1.99		b25y12	621.3178	621.3187	1.45

Table S9 (Cont.)

b26y11	621.3178	621.3187	1.45		b16y26	1106.3701	1106.3711	0.90
b23y15	636.2923	636.2923	0.00		b18y26	1342.4974	1342.4948	-1.94
b24y14	636.2923	636.2923	0.00		b24y21	1372.5886	1372.5904	1.31
b7y31	651.2048	651.2065	2.61		b18y27	1413.5345	1413.5375	2.12
b11y27	677.2382	677.2367	-2.21		b19y26	1413.5345	1413.5375	2.12
b25y13	678.3392	678.3406	2.06		b19y27	1484.5716	1484.5743	1.82
b15y21	737.3036	737.3033	-0.41		b24y22	1532.6193	1532.6166	-1.76
b22y17	737.3036	737.3033	-0.41		b25y22	1645.7033	1645.7062	1.76
b10y30	776.3066	776.3086	2.58		b18y30	1672.6336	1672.6348	0.72
b31y8	784.4063	784.4075	1.53		b19y29	1672.6336	1672.6348	0.72
b9y31	821.3104	821.3127	2.80		b20y28	1672.6336	1672.6348	0.72
b16y24	875.3023	875.3041	2.06		b24y24	1746.7146	1746.7127	-1.09
b17y23	875.3023	875.3041	2.06		b25y23	1760.7303	1760.7314	0.62
b18y22	897.3343	897.3337	-0.67		b24y25	1817.7517	1817.7567	2.75
b13y27	934.3217	934.3240	2.46		b25y24	1859.7987	1859.8018	1.67
b14y26	934.3217	934.3240	2.46		b25y25	1930.8358	1930.8370	0.62
b16y25	946.3394	946.3378	-1.69		b24y26	1977.7824	1977.7830	0.30
b19y22	968.3714	968.3697	-1.76		b24y27	2048.8195	2048.8137	-2.83
b25y16	985.5037	985.5062	2.54		b25y26	2090.8665	2090.8713	2.30
b18y23	1012.3612	1012.3637	2.47		b25y27	2161.9036	2161.9006	-1.39
b19y23	1083.3983	1083.3981	-0.18		b25y29	2349.9655	2349.9644	-0.47

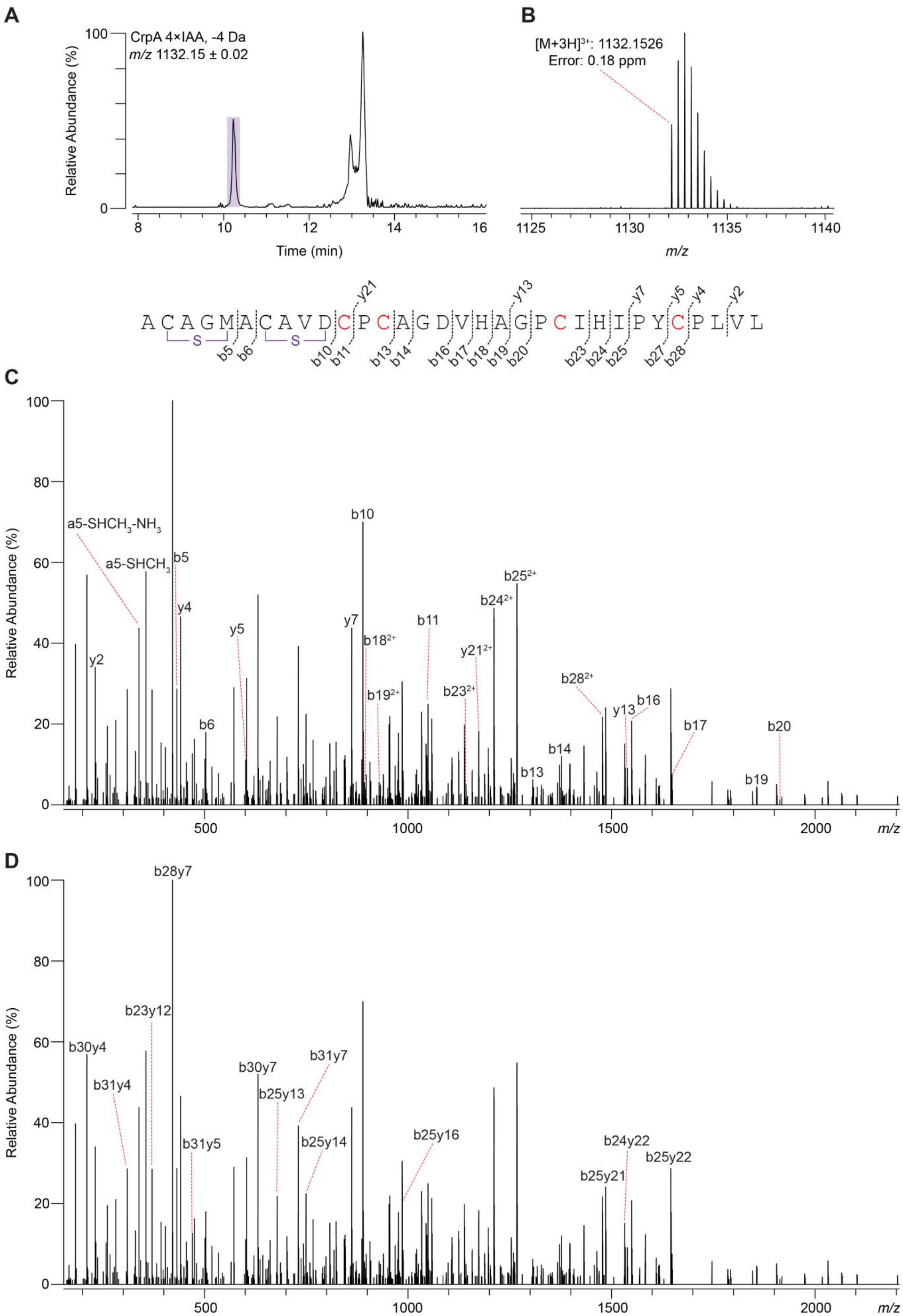
A
CrpA 4×IAA, -4 Da
m/z 1132.15 ± 0.02
Relative Abundance (%)
Time (min)
B
[M+3H]3+: 1132.1526
Error: 0.18 ppm
m/z
A C A G M A C A V D C P C A G D V H A G P C I H I P Y C P L V L
S
S
y21
y13
y7
y5
y4
y2
b5
b6
b10
b11
b13
b14
b16
b17
b18
b19
b20
b23
b24
b25
b27
b28
C
Relative Abundance (%)
a5-SHCH3-NH3
a5-SHCH3
b5
y4
y2
b6
y5
y7
b10
b18 2+
b19 2+
b11
b23 2+
y21 2+
b24 2+
b25 2+
b28 2+
y13
b16
b13
b14
b17
b19
b20
m/z
D
Relative Abundance (%)
b28y7
b23y12
b30y4
b31y4
b30y7
b31y7
b25y13
b25y14
b31y5
b25y16
b24y22
b25y21
b25y22
m/z

Figure S16 (previous page): HR-LC-MS/MS analysis of 4×IAA labeled CrpA^{C2C7}-modified tryptic fragment. (A) EIC for 4×IAA CrpA$_{CITryp}$ where Cys2 and Cys7 are modified, and (B) the HR-MS spectrum of the peptide fragment. The selected molecular ion (*m/z* 1132.15 ± 0.02) in the purple shaded area was fragmented via higher-energy collision induced dissociation (HCD). (C) *Top:* Summary of observed HCD daughter ions. Red cysteine indicates IAA modification. Hypothesized thioether bond is indicated in purple. *Bottom:* HCD MS/MS spectrum. All ions are shown in the 1+ charge state unless otherwise stated. The a5 ion (ACAGM) was observed with losses of $SHCH_3$ and NH_3 from methionine, neutral losses with precedence in the proteomics literature.[11,12] (D) An identical spectrum to (C) with key internal daughter ions indicated. Where masses can correspond to multiple possible internal fragments, the most likely one is shown. See **Table S10** for exact masses and errors of all observed daughter ions.

Table S10: Assignment of HCD daughter ions for the 4×IAA-labeled CrpA^{C2C7}-modified tryptic fragment. All ions are shown in the 1+ charge state unless otherwise stated. Corresponding HCD spectrum is shown in **Figure S16**. All observed internal ions are included, not just the key ones highlighted in **Figure S16**. Multiple possible internal fragments are listed if they have the same *m/z*.

Ion	Calculated *m/z*	Observed *m/z*	Error (ppm)		Ion	Calculated *m/z*	Observed *m/z*	Error (ppm)
$[M+3H]^{3+}$	1132.1524	1132.1526	0.18		$b19^{2+}$	928.8358	928.8367	0.97
$b5^{+}$	432.1370	432.1373	0.69		$b20^{+}$	1913.6858	1913.6835	1.20
a5-$SHCH_3$	356.1387	356.1391	1.12		$b23^{2+}$	1142.4303	1142.4288	1.31
a5-$SHCH_3$-NH_3	339.1122	339.1126	1.18		$b24^{2+}$	1210.9597	1210.9623	2.15
$b6^{+}$	503.1742	503.1741	0.20		$b25^{2+}$	1267.5018	1267.5049	2.45
$b10^{+}$	889.3002	889.3022	2.25		$b28^{2+}$	1477.5752	1477.5802	3.38
$b11^{+}$	1049.3309	1049.333	2.00		$y2^{+}$	231.1703	231.1706	1.30
$b13^{+}$	1306.4143	1306.4099	3.37		$y4^{+}$	441.3071	441.3073	0.45
$b14^{+}$	1377.4515	1377.4477	2.76		$y5^{+}$	601.3378	601.3389	1.83
$b16^{+}$	1549.4999	1549.5013	0.90		$y7^{+}$	861.4539	861.4549	1.16
$b17^{+}$	1648.5683	1648.5629	3.28		$y13^{+}$	1538.7859	1538.7905	2.99
$b18^{2+}$	893.3172	893.3177	0.56		$y21^{2+}$	1173.5633	1173.5671	3.24
$b19^{+}$	1856.6643	1856.6586	3.07					

Ion	Calculated *m/z*	Observed *m/z*	Error (ppm)		Ion	Calculated *m/z*	Observed *m/z*	Error (ppm)
b30y4	211.1441	211.1443	0.95		b29y8	631.2909	631.2920	1.74
b31y4	310.2125	310.2129	1.29		b30y7	631.2909	631.2920	1.74
b23y12	371.1748	371.1753	1.35		b23y15	636.2923	636.2937	2.20
b30y5	371.1748	371.1753	1.35		b24y14	636.2923	636.2937	2.20
b28y7	421.1541	421.1545	0.95		b16y22	661.2069	661.2071	0.30
b29y6	421.1541	421.1545	0.95		b25y13	678.3392	678.3404	1.77
b31y5	470.2432	470.2438	1.28		b31y7	730.3593	730.3598	0.68
b18y19	480.2201	480.2215	2.92		b15y21	737.3036	737.3039	0.41
b19y18	480.2201	480.2215	2.92		b22y17	737.3036	737.3039	0.41
b20y17	480.2201	480.2215	2.92		b25y14	749.3763	749.3781	2.40
b29y7	518.2068	518.2073	0.96		b10y31	818.2631	818.2634	0.37
b28y8	534.2381	534.2377	-0.75		b20y21	865.3622	865.3599	-2.66
b30y6	534.2381	534.2377	-0.75		b21y20	865.3622	865.3599	-2.66
b24y13	565.2552	565.2568	2.83		b22y19	865.3622	865.3599	-2.66
b25y12	621.3178	621.3189	1.77		b25y15	886.4353	886.4371	2.03
b26y11	621.3178	621.3189	1.77		b18y22	897.3343	897.3333	-1.11

Table S10 (Cont.)

b25y16	985.5037	985.5028	-0.91		b26y20	1485.6726	1485.6758	2.15
b28y13	1098.4860	1098.4834	-2.37		b24y22	1532.6193	1532.6213	1.30
b25y21	1485.6726	1485.6758	2.15		b25y22	1645.7033	1645.7059	1.58

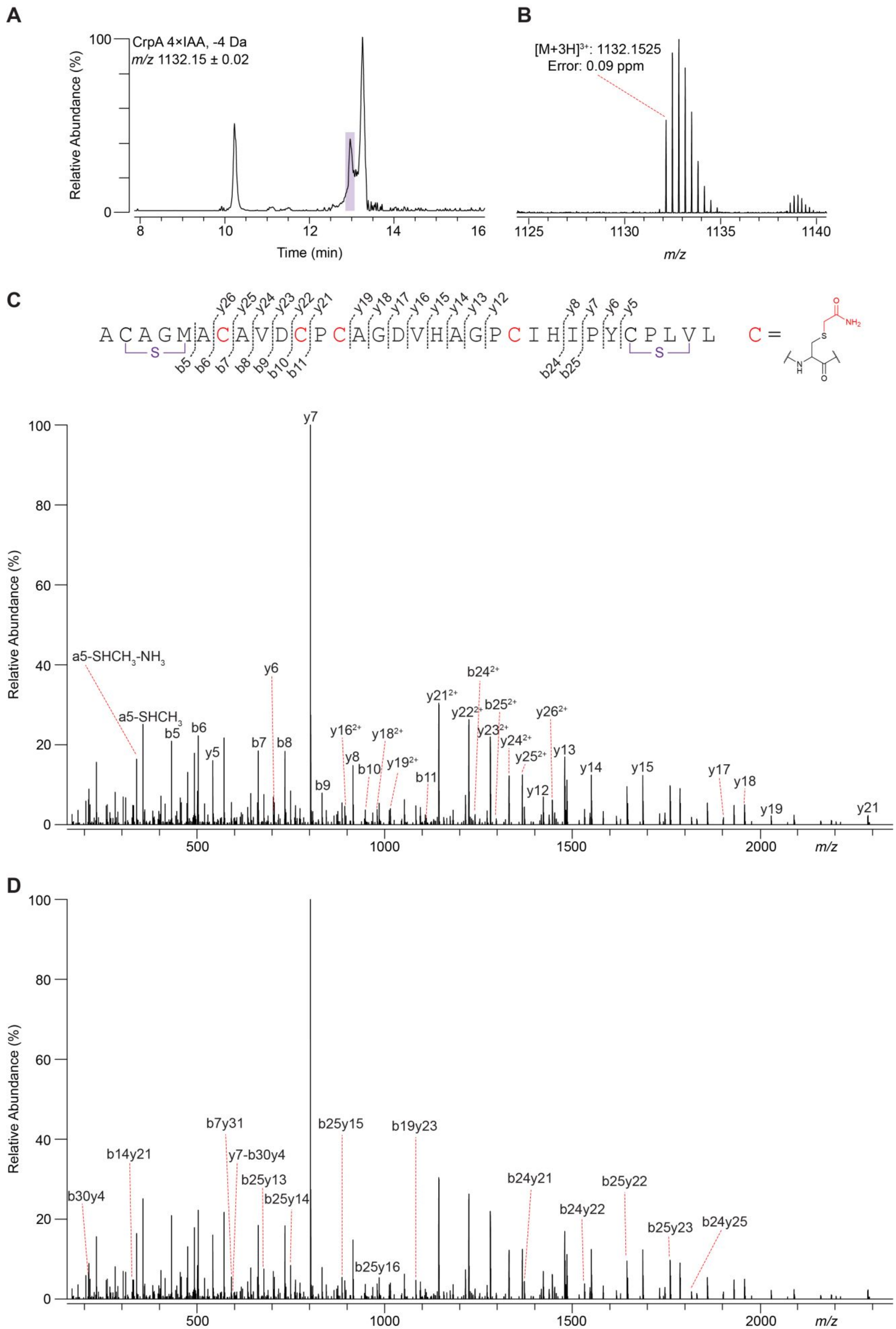
A
CrpA 4×IAA, -4 Da
m/z 1132.15 ± 0.02
Relative Abundance (%)
Time (min)
B
[M+3H]3+: 1132.1525
Error: 0.09 ppm
m/z
C
ACAGMACAVDCPCAGDVHAGPCIHIPYCPLVL
C =
Relative Abundance (%)
y7
y6
a5-SHCH3-NH3
a5-SHCH3
b5
b6
y5
b7
b8
b9
y16 2+
y8
b10
y18 2+
y19 2+
b11
y21 2+
y22 2+
b24 2+
b25 2+
y23 2+
y24 2+
y25 2+
y26 2+
y12
y13
y14
y15
y17
y18
y19
y21
m/z
D
Relative Abundance (%)
b30y4
b14y21
b7y31
y7-b30y4
b25y13
b25y14
b25y15
b25y16
b19y23
b24y21
b24y22
b25y22
b25y23
b24y25
m/z

Figure S17 (previous page): HR-LC-MS/MS analysis of 4×IAA labeled CrpA^{C2C28}-modified tryptic fragment. (A) EIC for 4×IAA CrpA$_{CITryp}$ where Cys2 and Cys28 are modified, and (B) the HR-MS spectrum of the peptide fragment. The selected molecular ion (*m/z* 1132.15 ± 0.02) in the purple shaded area was fragmented via higher-energy collision induced dissociation (HCD). (C) *Top:* Summary of observed HCD daughter ions. Red cysteine indicates IAA modification. Hypothesized thioether bond is indicated in purple. *Bottom:* HCD MS/MS spectrum. All ions are shown in the 1+ charge state unless otherwise stated. The a5 ion (ACAGM) was observed with losses of $SHCH_3$ and NH_3 from methionine, neutral losses with precedence in the proteomics literature.[11,12] (D) An identical spectrum to (C) with key internal daughter ions indicated. Where masses can correspond to multiple possible internal fragments, the most likely one is shown. See **Table S11** for exact masses and errors of all observed daughter ions.

Table S11: Assignment of HCD daughter ions for the 4×IAA-labeled CrpA^{C2C28}-modified tryptic fragment. All ions are shown in the 1+ charge state unless otherwise stated. Corresponding HCD spectrum is shown in **Figure S17**. All observed internal ions are included, not just the key ones highlighted in **Figure S17**. Multiple possible internal fragments are listed if they have the same *m/z*.

Ion	Calculated *m/z*	Observed *m/z*	Error (ppm)		Ion	Calculated *m/z*	Observed *m/z*	Error (ppm)
$[M+3H]^{3+}$	1132.1524	1132.1525	0.09		$y13^{+}$	1479.7488	1479.7488	0.00
a5-$SHCH_3$	356.1387	356.1393	1.68		$y14^{+}$	1550.7859	1550.7881	1.42
a5-$SHCH_3$-NH_3	339.1122	339.1128	1.77		$y15^{+}$	1687.8448	1687.8458	0.59
$b5^{+}$	432.1370	432.1375	1.16		$y16^{2+}$	893.9602	893.9613	1.23
$b6^{+}$	503.1742	503.1748	1.19		$y17^{+}$	1901.9402	1901.9364	-2.00
$b7^{+}$	663.2048	663.2054	0.90		$y18^{+}$	1958.9616	1958.96	-0.82
$b8^{+}$	734.2419	734.2414	-0.68		$y18^{2+}$	979.9844	979.9817	-2.76
$b9^{+}$	833.3104	833.3098	-0.72		$y19^{+}$	2029.9987	2029.9974	-0.64
$b10^{+}$	948.3373	948.34	2.85		$y19^{2+}$	1015.503	1015.5044	1.38
$b11^{+}$	1108.3680	1108.3671	-0.81		$y21^{+}$	2287.0822	2287.0755	-2.93
$b24^{2+}$	1240.4783	1240.476	-1.85		$y21^{2+}$	1144.0447	1144.0459	1.05
$b25^{2+}$	1297.0203	1297.0186	-1.31		$y22^{2+}$	1224.0601	1224.0624	1.88
$y5^{+}$	542.3007	542.3013	1.11		$y23^{2+}$	1281.5735	1281.5776	3.20
$y6^{+}$	705.3641	705.3637	-0.57		$y24^{2+}$	1331.1078	1331.1097	1.43
$y7^{+}$	802.4168	802.4179	1.37		$y25^{2+}$	1366.6263	1366.629	1.98
$y8^{+}$	915.5009	915.5023	1.53		$y26^{2+}$	1446.6417	1446.6432	1.04
$y12^{+}$	1422.7273	1422.7272	-0.07					

Ion	Calculated *m/z*	Observed *m/z*	Error (ppm)		Ion	Calculated *m/z*	Observed *m/z*	Error (ppm)
b30y4	211.1441	211.1443	0.95		b17y21	600.2447	600.2453	1.00
b15y20	289.0965	289.0966	0.35		b25y12	621.3178	621.3195	2.74
b14y21	329.1278	329.1282	1.22		b26y11	621.3178	621.3195	2.74
b9y26	331.1435	331.1437	0.60		b23y15	636.2923	636.2939	2.51
b15y21	386.1493	386.15	1.81		b24y14	636.2923	636.2939	2.51
b22y14	386.1493	386.15	1.81		b25y13	678.3392	678.34	1.18
b9y27	402.1806	402.1812	1.49		b25y14	749.3763	749.3775	1.60
b14y22	489.1585	489.1582	-0.61		b15y24	760.2753	760.2771	2.37
b24y13	565.2552	565.2554	0.35		b17y22	760.2753	760.2771	2.37
b7y31	592.1677	592.1676	-0.17		b9y31	762.2733	762.2736	0.39
y7-b30y4	592.2800	592.2808	1.35		b31y8	784.4063	784.4085	2.80

Table S11 (Cont.)

b19y21	808.3407	808.3405	-0.25		b19y23	1083.3983	1083.3974	-0.83
b20y21	865.3622	865.3634	1.39		b20y23	1140.4198	1140.418	-1.58
b21y20	865.3622	865.3634	1.39		b25y18	1157.5521	1157.5548	2.33
b22y19	865.3622	865.3634	1.39		b25y19	1228.5892	1228.5864	-2.28
b24y16	872.4196	872.4205	1.03		b24y21	1372.5886	1372.5866	-1.46
b16y24	875.3023	875.3012	-1.26		b24y22	1532.6193	1532.6224	2.02
b17y23	875.3023	875.3012	-1.26		b25y22	1645.7033	1645.7043	0.61
b25y15	886.4353	886.4362	1.02		b24y24	1746.7146	1746.7151	0.29
b18y22	897.3343	897.3337	-0.67		b25y23	1760.7303	1760.7267	-2.04
b25y16	985.5037	985.5053	1.62		b24y25	1817.7517	1817.7517	0.00

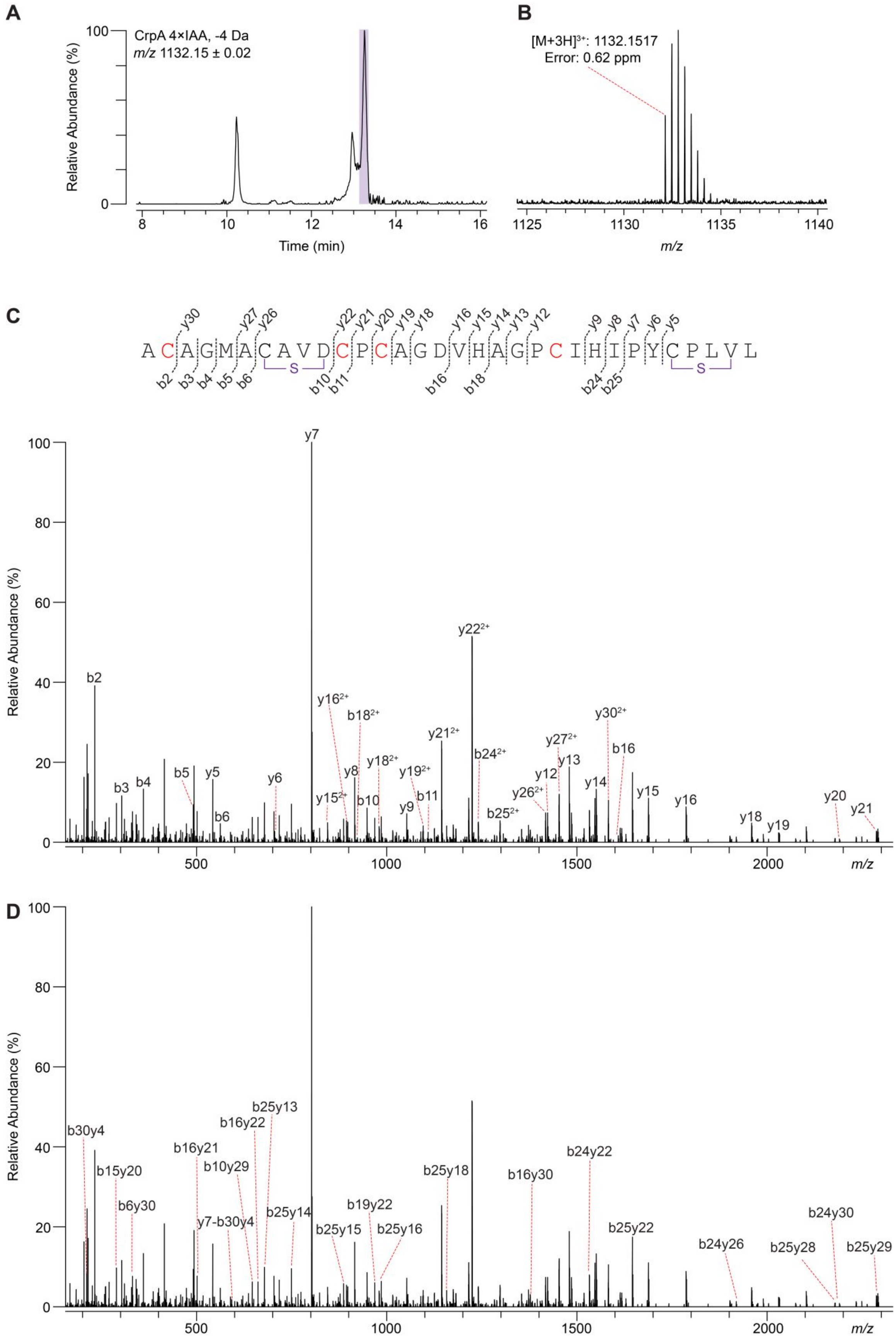
A
CrpA 4×IAA, -4 Da
m/z 1132.15 ± 0.02
Relative Abundance (%)
Time (min)
B
[M+3H]3+: 1132.1517
Error: 0.62 ppm
m/z
C
ACAGMACAVDCPCAGDVHAGPCIHIPYCPLVL
S
S
y30 y27 y26 y22 y21 y20 y19 y18 y16 y15 y14 y13 y12 y9 y8 y7 y6 y5
b2 b3 b4 b5 b6 b10 b11 b16 b18 b24 b25
y7
y22 2+
b2
y16 2+
b18 2+
y21 2+
y18 2+
y19 2+
b24 2+
y27 2+
y30 2+
b16
y13
y12
y26 2+
y14
y15
y16
b3
b4
b5
y5
b6
y6
y15 2+
y8
b10
y9
b11
b25 2+
y18
y19
y20
y21
D
b30y4
b15y20
b6y30
b16y21
b10y29
b16y22
b25y13
y7-b30y4
b25y14
b25y15
b19y22
b25y16
b25y18
b16y30
b24y22
b25y22
b24y26
b25y28
b24y30
b25y29
m/z

Figure S18 (previous page): HR-LC-MS/MS analysis of 4×IAA labeled CrpA^{C7C28}-modified tryptic fragment. (A) EIC for the 4×IAA CrpA$_{CITryp}$ where Cys7 and Cys28 are modified, and (B) the HR-MS spectrum of the peptide fragment. The selected molecular ion (*m/z* 1132.15 ± 0.02) in the purple shaded area was fragmented via higher-energy collision induced dissociation (HCD). (C) *Top:* Summary of observed HCD daughter ions. Red cysteine indicates IAA modification. Hypothesized thioether bond is indicated in purple. *Bottom:* HCD MS/MS spectrum. All ions are shown in the 1+ charge state unless otherwise stated. (D) An identical spectrum to (C) with key internal daughter ions indicated. Where masses can correspond to multiple possible internal fragments, the most likely one is shown. See **Table S12** for exact masses and errors of all observed daughter ions.

Table S12: Assignment of HCD daughter ions for the 4×IAA-labeled CrpA^{C2C7}-modified tryptic fragment. All ions are shown in the 1+ charge state unless otherwise stated. Corresponding HCD spectrum is shown in **Figure S18**. All observed internal ions are included, not just the key ones shown in **Figure S18**. Multiple possible internal fragments are listed if they have the same *m/z*.

Ion	Calculated *m/z*	Observed *m/z*	Error (ppm)	Ion	Calculated *m/z*	Observed *m/z*	Error (ppm)
[M+3H]$^{3+}$	1132.1524	1132.1517	0.62	y13^{+}	1479.7488	1479.7513	1.69
b2^{+}	232.0751	232.0756	2.15	y14^{+}	1550.7859	1550.7897	2.45
b3^{+}	303.1122	303.1128	1.98	y15^{+}	1687.8448	1687.8474	1.54
b4^{+}	360.1337	360.1343	1.67	y15^{2+}	844.4260	844.4276	1.89
b5^{+}	491.1741	491.1748	1.43	y16^{+}	1786.9132	1786.9163	1.73
b6^{+}	562.2113	562.2118	0.89	y16^{2+}	893.9602	893.9622	2.24
b10^{+}	948.3373	948.3388	1.58	y18^{+}	1958.9616	1958.964	1.23
b11^{+}	1108.3680	1108.3647	2.98	y18^{2+}	979.9844	979.9861	1.73
b16^{+}	1608.5370	1608.5400	1.87	y19^{+}	2029.9987	2030.0029	2.07
b18^{2+}	922.8358	922.8367	0.98	y20^{+}	2190.0294	2190.0313	0.87
b24^{2+}	1240.4783	1240.4814	2.50	y20^{2+}	1095.5183	1095.5214	2.83
b25^{2+}	1297.0203	1297.0234	2.39	y21^{+}	2287.0822	2287.0886	2.80
y5^{+}	542.3007	542.3010	0.55	y21^{2+}	1144.0447	1144.0468	1.84
y6^{+}	705.3641	705.3635	0.85	y22^{2+}	1224.0601	1224.0619	1.47
y7^{+}	802.4168	802.4148	2.49	y26^{2+}	1417.1231	1417.1239	0.56
y8^{+}	915.5009	915.5023	1.53	y27^{2+}	1452.6417	1452.642	0.21
y9^{+}	1052.5598	1052.5605	0.67	y30^{2+}	1582.1912	1582.1947	2.21
y12^{+}	1422.7273	1422.7317	3.09				

Ion	Calculated *m/z*	Observed *m/z*	Error (ppm)	Ion	Calculated *m/z*	Observed *m/z*	Error (ppm)
b30y4	211.1441	211.1444	1.42	b10y26	387.1333	387.1338	1.29
b5y30	260.1063	260.1070	2.69	b13y22	418.1214	418.1220	1.43
b6y29	260.1063	260.1070	2.69	b5y31	420.1370	420.1382	2.86
b4y31	289.0965	289.0970	1.73	b10y27	458.1704	458.1716	2.62
b15y20	289.0965	289.0970	1.73	b18y19	480.2201	480.2193	-1.67
b19y16	308.1717	308.1725	2.60	b19y18	480.2201	480.2193	-1.67
b22y13	315.1122	315.1127	1.59	b20y17	480.2201	480.2193	-1.67
b6y30	331.1435	331.1442	2.11	b14y22	489.1585	489.1585	0.00
b15y21	386.1493	386.1499	1.55	b16y21	501.1762	501.1771	1.80
b22y14	386.1493	386.1499	1.55	b24y12	508.2337	508.2350	2.56

Table S12 (Cont.)

b11y26	547.1640	547.1643	0.55		b11y30	877.3002	877.2978	-2.74
b24y13	565.2552	565.2548	-0.71		b25y15	886.4353	886.4370	1.92
b10y28	589.2109	589.2115	1.02		b19y22	968.3714	968.3728	1.45
y7-b30y4	592.2800	592.2792	-1.35		b25y16	985.5037	985.5046	0.91
b17y21	600.2447	600.2443	-0.67		b20y22	1025.3928	1025.3925	-0.29
b25y12	621.3178	621.3175	-0.48		b22y20	1025.3928	1025.3925	-0.29
b26y11	621.3178	621.3175	-0.48		b25y18	1157.5521	1157.5546	2.16
b23y15	636.2923	636.2925	0.31		b25y19	1228.5892	1228.5927	2.85
b24y14	636.2923	636.2925	0.31		b16y29	1306.4321	1306.4313	-0.61
b10y29	646.2324	646.2338	2.17		b18y27	1354.4974	1354.4952	-1.62
b16y22	661.2069	661.2085	2.42		b19y26	1354.4974	1354.4952	-1.62
b25y13	678.3392	678.3400	1.18		b16y30	1377.4692	1377.4663	-2.11
b15y21	737.3036	737.3056	2.71		b23y22	1395.5603	1395.5603	0.00
b22y17	737.3036	737.3056	2.71		b24y22	1532.6193	1532.6219	1.70
b25y14	749.3763	749.3771	1.07		b18y30	1613.5965	1613.6000	2.17
b17y22	760.2753	760.2772	2.50		b19y29	1613.5965	1613.6000	2.17
b31y8	784.4063	784.4055	-1.02		b20y28	1613.5965	1613.6000	2.17
b11y29	806.2631	806.2648	2.11		b25y22	1645.7033	1645.7041	0.49
b20y21	865.3622	865.3621	-0.12		b24y26	1918.7453	1918.7471	0.94
b21y20	865.3622	865.3621	-0.12		b25y28	2233.907	2233.9021	-2.19
b22y19	865.3622	865.3621	-0.12		b24y30	2248.8815	2248.884	1.11
b10y31	877.3002	877.2978	-2.74		b25y29	2290.9284	2290.9329	1.96
b18y22	897.3343	897.3358	1.67					

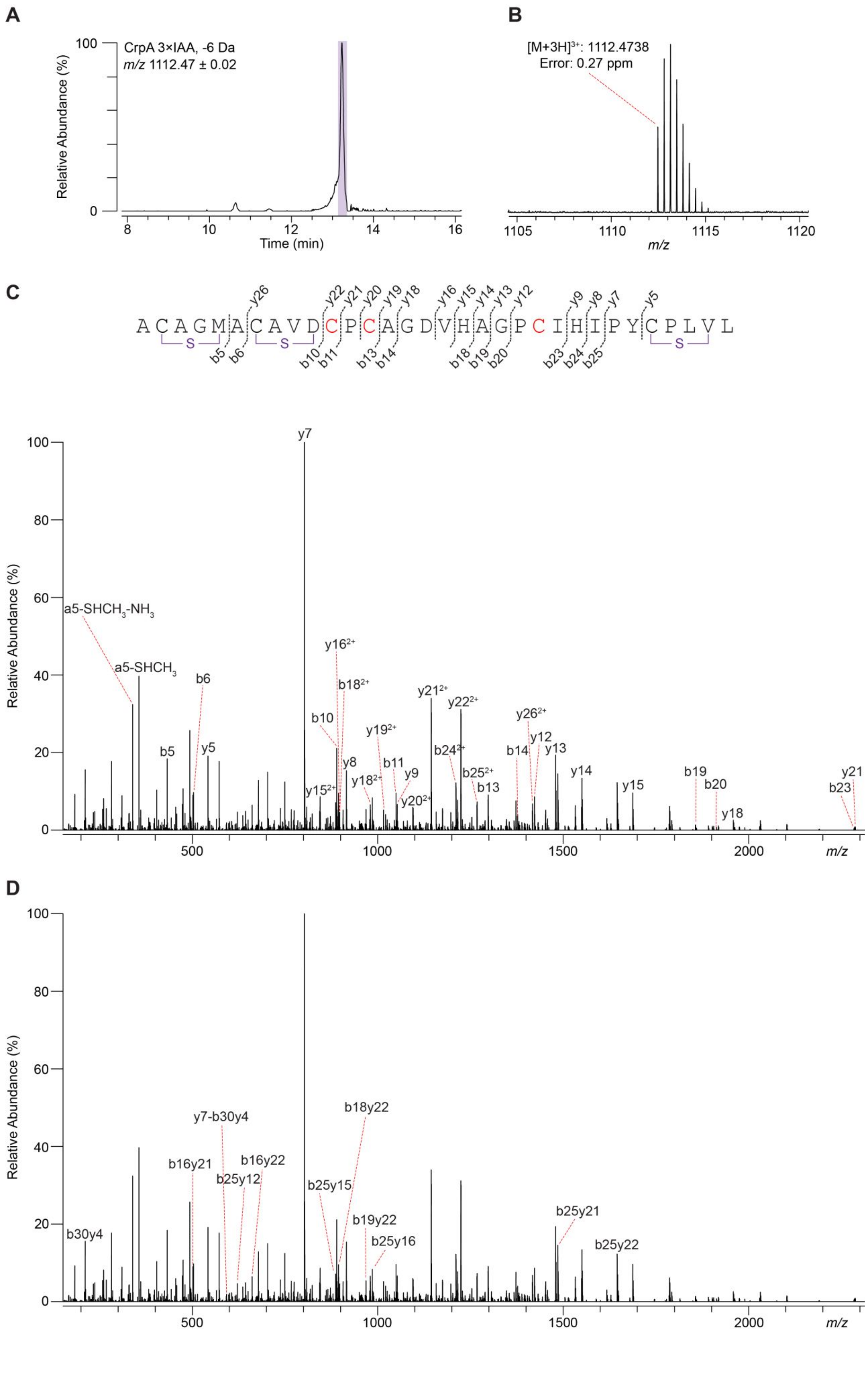
A
CrpA 3×IAA, -6 Da
m/z 1112.47 ± 0.02
Relative Abundance (%)
Time (min)
B
[M+3H]3+: 1112.4738
Error: 0.27 ppm
m/z
C
A C A G M A C A V D C P C A G D V H A G P C I H I P Y C P L V L
S
S
S
y26 y22 y21 y20 y19 y18 y16 y15 y14 y13 y12 y9 y8 y7 y5
b5 b6 b10 b11 b13 b14 b18 b19 b20 b23 b24 b25
Relative Abundance (%)
y7
a5-SHCH3-NH3
a5-SHCH3
b6
b5
y5
y16 2+
b18 2+
b10
y8
y15 2+
y18 2+
y19 2+
b11
y9
y20 2+
y21 2+
y22 2+
b24 2+
b25 2+
b13
y26 2+
b14
y12
y13
y14
y15
b19
b20
y18
y21
b23
m/z
D
Relative Abundance (%)
y7-b30y4
b18y22
b16y21
b16y22
b25y12
b25y15
b19y22
b25y16
b30y4
b25y21
b25y22
m/z

Figure S19 (previous page): HR-LC-MS/MS analysis of 3×IAA labeled CrpA^{C2C7C28}-modified tryptic fragment. (A) EIC for the 3×IAA CrpA$_{CITryp}$ where Cys2, Cys7, and Cys28 are modified, and (B) the HR-MS spectrum of the peptide fragment. The selected molecular ion (*m/z* 1112.47 ± 0.02) in the purple shaded area was fragmented via higher-energy collision induced dissociation (HCD). (C) *Top:* Summary of observed HCD daughter ions. Red cysteine indicates IAA modification. Hypothesized thioether bond is indicated in purple. *Bottom:* HCD MS/MS spectrum. All ions are shown in the 1+ charge state unless otherwise stated. The a5 ion (ACAGM) was observed with losses of $SHCH_3$ and NH_3 from methionine, neutral losses with precedence in the proteomics literature.[11,12] (D) An identical spectrum to (C) with key internal daughter ions indicated. Where masses can correspond to multiple possible internal fragments, the most likely one is shown. See **Table S13** for exact masses and errors of all observed daughter ions.

Table S13: Assignment of HCD daughter ions for the 3×IAA-labeled CrpA^{C2C7C28}-modified tryptic fragment. All ions are shown in the 1+ charge state unless otherwise stated. Corresponding HCD spectrum is shown in **Figure S19**. All observed internal ions are included, not just the key ones highlighted in **Figure S19**. Multiple possible internal fragments are listed if they have the same *m/z*.

Ion	Calculated *m/z*	Observed *m/z*	Error (ppm)		Ion	Calculated *m/z*	Observed *m/z*	Error (ppm)
$[M+3H]^{3+}$	1112.4735	1112.4738	0.27		$y8^{+}$	915.5009	915.5025	1.75
$b5^{+}$	432.1370	432.1373	0.69		$y9^{+}$	1052.5598	1052.5593	-0.48
a5-$SHCH_3$	356.1387	356.1392	1.40		$y12^{+}$	1422.7273	1422.7307	2.39
a5-$SHCH_3$-NH_3	339.1122	339.1127	1.47		$y13^{+}$	1479.7488	1479.7480	-0.54
$b6^{+}$	503.1742	503.1744	0.40		$y14^{+}$	1550.7859	1550.7876	1.10
$b10^{+}$	889.3002	889.3021	2.14		$y15^{+}$	1687.8448	1687.8455	0.41
$b11^{+}$	1049.3309	1049.3322	1.24		$y15^{2+}$	844.4260	844.4282	2.61
$b13^{+}$	1306.4143	1306.4157	1.07		$y16^{2+}$	893.9602	893.9621	2.13
$b14^{+}$	1377.4515	1377.4515	0.00		$y18^{+}$	1958.9616	1958.9600	-0.82
$b18^{2+}$	893.3172	893.3179	0.78		$y18^{2+}$	979.9844	979.9871	2.76
$b19^{+}$	1856.6643	1856.6681	2.05		$y19^{2+}$	1015.5030	1015.5057	2.66
$b20^{+}$	1913.6858	1913.6864	0.31		$y20^{2+}$	1095.5183	1095.5190	0.64
$b23^{+}$	2283.8533	2283.8470	-2.76		$y21^{+}$	2287.0822	2287.0757	-2.84
$b24^{2+}$	1210.9597	1210.9579	-1.49		$y21^{2+}$	1144.0447	1144.0461	1.22
$b25^{2+}$	1267.5018	1267.5018	0.00		$y22^{2+}$	1224.0601	1224.0612	0.90
$y5^{+}$	542.3007	542.3010	0.55		$y26^{2+}$	1417.1231	1417.1248	1.20
$y7^{+}$	802.4168	802.4178	1.25					

Ion	Calculated *m/z*	Observed *m/z*	Error (ppm)		Ion	Calculated *m/z*	Observed *m/z*	Error (ppm)
b30y4	211.1441	211.1442	0.47		b23y15	636.2923	636.2935	1.89
b19y16	308.1717	308.1722	1.62		b24y14	636.2923	636.2935	1.89
b5y31	361.0999	361.1005	1.66		b16y22	661.2069	661.2076	1.06
b18y19	480.2201	480.2199	-0.42		b25y13	678.3392	678.3400	1.18
b19y18	480.2201	480.2199	-0.42		b15y21	737.3036	737.3047	1.49
b20y17	480.2201	480.2199	-0.42		b22y17	737.3036	737.3047	1.49
b16y21	501.1762	501.1747	-2.99		b25y14	749.3763	749.3779	2.14
b24y13	565.2552	565.2547	-0.88		b19y21	808.3407	808.3417	1.24
y7-b30y4	592.2800	592.2806	1.01		b10y31	818.2631	818.2632	0.12
b25y12	621.3178	621.3190	1.93		b24y16	872.4196	872.4208	1.38
b26y11	621.3178	621.3190	1.93		b25y15	886.4353	886.4367	1.58

Table S13 (Cont.)

b18y22	897.3343	897.3363	2.23	b25y19	1228.5892	1228.5910	1.47
b19y22	968.3714	968.3737	2.38	b24y21	1372.5886	1372.5887	0.07
b25y16	985.5037	985.5050	1.32	b25y21	1485.6726	1485.6741	1.01
b20y22	1025.3928	1025.3937	0.88	b26y20	1485.6726	1485.6741	1.01
b22y20	1025.3928	1025.3937	0.88	b25y22	1645.7033	1645.7034	0.06
b25y18	1157.5521	1157.5549	2.42				

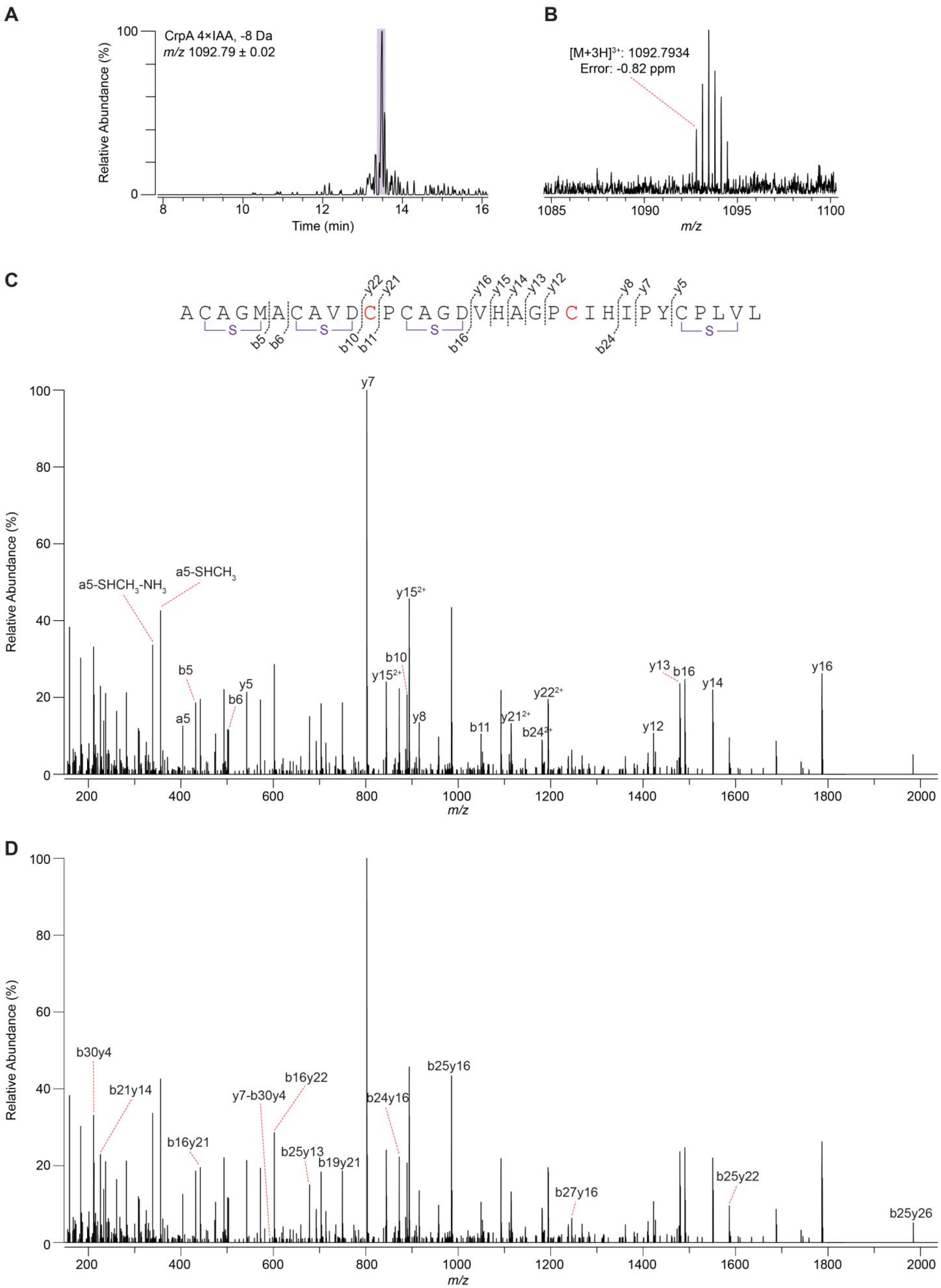
A
CrpA 4×IAA, -8 Da
m/z 1092.79 ± 0.02
Relative Abundance (%)
Time (min)
B
[M+3H]3+: 1092.7934
Error: -0.82 ppm
m/z
C
ACAGMACAVDCPCAGDVHAGPCIHIPYCPLVL
y22 y21 y16 y15 y14 y13 y12 y8 y7 y5
b5 b6 b10 b11 b16 b24
S
Relative Abundance (%)
y7
a5-SHCH3-NH3
a5-SHCH3
y15 2+
b5
a5
b6
y5
b10
y8
b11
y21 2+
y22 2+
b24 2+
y12
y13
b16
y14
y16
m/z
D
Relative Abundance (%)
b30y4
b21y14
b16y21
y7-b30y4
b16y22
b25y13
b19y21
b24y16
b25y16
b27y16
b25y22
b25y26
m/z

Figure S20 (previous page): HR-LC-MS/MS analysis of 2×IAA labeled CrpA^{C2C7C13C28}-modified tryptic fragment. (A) EIC for the 2×IAA CrpA$_{CITryp}$ where Cys2, Cys7, Cys13, and Cys28 are modified, and (B) the HR-MS spectrum of the peptide fragment. The selected molecular ion (*m/z* 1092.79 ± 0.02) in the purple shaded area was fragmented via higher-energy collision induced dissociation (HCD). (C) *Top:* Summary of observed HCD daughter ions. Red cysteine indicates IAA modification. Hypothesized thioether bond is indicated in purple. *Bottom:* HCD MS/MS spectrum. All ions are shown in the 1+ charge state unless otherwise stated. The a5 ion (ACAGM) was observed with losses of $SHCH_3$ and NH_3 from methionine, neutral losses with precedence in the proteomics literature.[11,12] (D) An identical spectrum to (C) with key internal daughter ions indicated. Where masses can correspond to multiple possible internal fragments, the most likely one is shown. See **Table S14** for exact masses and errors of all observed daughter ions.

Table S14: Assignment of HCD daughter ions for the 2×IAA-labeled CrpA^{C2C7C13C28}-modified tryptic fragment. All ions are shown in the 1+ charge state unless otherwise stated. Corresponding HCD spectrum is shown in **Figure S20**. All observed internal ions are included, not just the key ones highlighted in **Figure S20**. Multiple possible internal fragments are listed if they have the same *m/z*.

Ion	Calculated *m/z*	Observed *m/z*	Error (ppm)		Ion	Calculated *m/z*	Observed *m/z*	Error (ppm)
$[M+3H]^{3+}$	1092.7943	1092.7934	-0.82		$y7^{+}$	802.4168	802.4185	2.12
$b5^{+}$	432.1370	432.1373	0.69		$y8^{+}$	915.5009	915.5030	2.29
a5-$SHCH_3$	356.1387	356.1394	1.97		$y12^{+}$	1422.7273	1422.7268	-0.35
a5-$SHCH_3$-NH_3	339.1122	339.1128	1.77		$y13^{+}$	1479.7488	1479.7502	0.95
$b6^{+}$	503.1742	503.1744	0.40		$y14^{+}$	1550.7859	1550.7830	-1.87
$b10^{+}$	889.3002	889.3027	2.81		$y15^{2+}$	844.4260	844.4282	2.61
$b11^{+}$	1049.3309	1049.3315	0.57		$y16^{+}$	1786.9132	1786.9170	2.13
$b16^{+}$	1490.4628	1490.463	0.13		$y16^{2+}$	893.9602	893.9627	2.80
$b24^{2+}$	1181.4412	1181.4412	0.00		$y21^{2+}$	1114.5262	1114.5245	-1.53
$y5^{+}$	542.3007	542.3012	0.92		$y22^{2+}$	1194.5415	1194.5423	0.67

Ion	Calculated *m/z*	Observed *m/z*	Error (ppm)		Ion	Calculated *m/z*	Observed *m/z*	Error (ppm)
b30y4	211.1441	211.1444	1.42		b25y14	749.3763	749.3765	0.27
b21y14	226.1186	226.1187	0.44		b18y22	838.2972	838.2982	1.19
b5y31	361.0999	361.1005	1.66		b24y16	872.4196	872.4217	2.41
b7y29	361.0999	361.1005	1.66		b25y15	886.4353	886.4333	-2.26
b20y16	365.1932	365.1934	0.55		b19y22	909.3343	909.3329	-1.54
b16y21	442.1391	442.1398	1.58		b25y16	985.5037	985.5056	1.93
y7-b30y4	592.2800	592.2796	-0.68		b27y16	1245.6198	1245.6234	2.89
b15y23	602.1698	602.1706	1.33		b18y27	1295.4603	1295.4609	0.46
b16y22	602.1698	602.1706	1.33		b19y26	1295.4603	1295.4609	0.46
b25y13	678.3392	678.3395	0.44		b24y22	1473.5822	1473.5804	-1.22
b23y16	735.3607	735.3615	1.09		b25y22	1586.6662	1586.6667	0.32
b19y21	749.3036	749.3051	2.00		b25y26	1972.7923	1972.7959	1.82

A

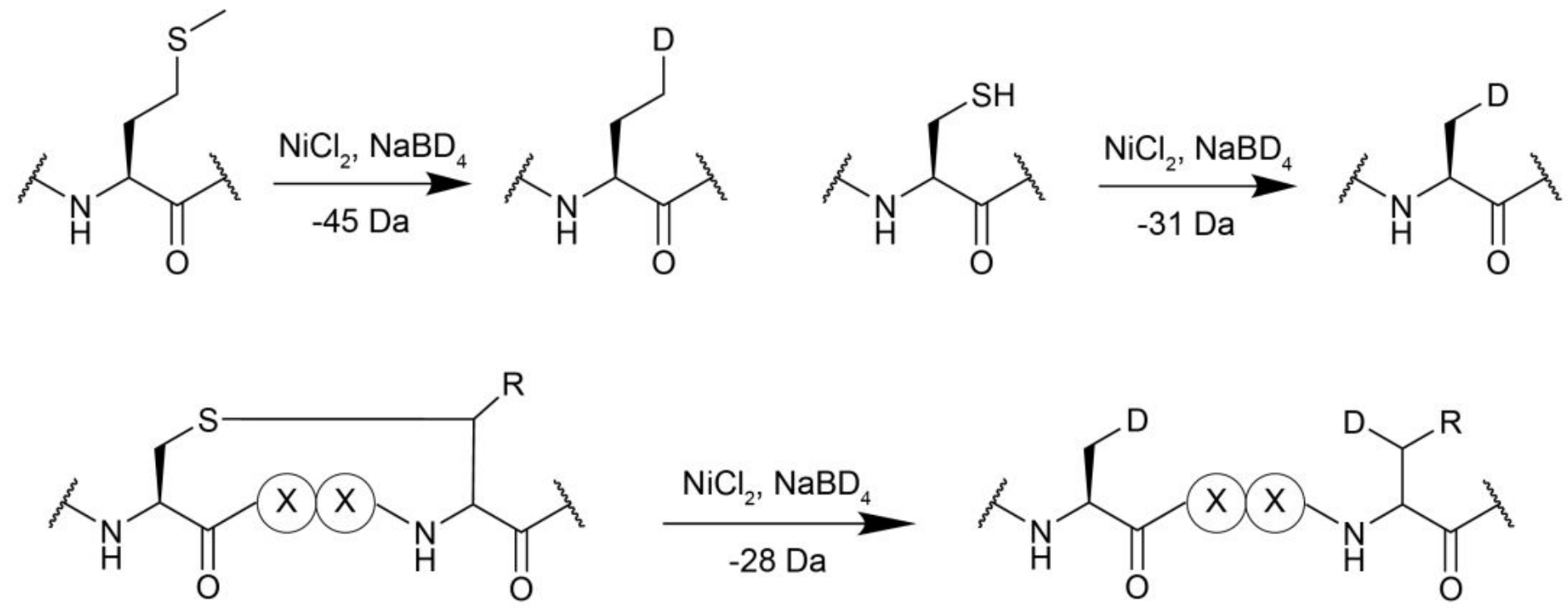

B

Precursor Mass Loss	Precursor Exact Mass Post Trypsinolysis and Desulfurization	Calculated $[M+3H]^{3+}$	Observed $[M+3H]^{3+}$	Error (ppm)
0 Da	2938.6046 Da	980.5422	980.5468	4.69
-2 Da	2939.6109 Da	980.8776	980.8778	0.20
-4 Da	2940.6172 Da	981.2130	981.2122	-0.82
-6 Da	2941.6234 Da	981.5484	981.5482	-0.20
-8 Da	2942.6297 Da	981.8839	981.8833	-0.61

C

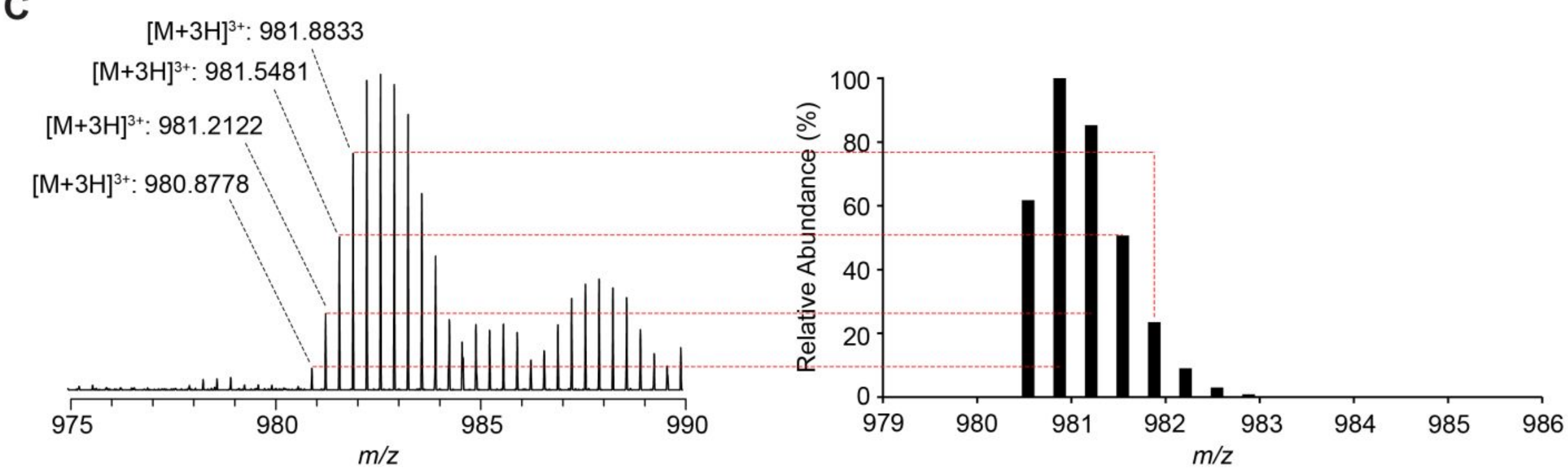

Figure S21: Summary of CrpA desulfurization. (A) Scheme for desulfurization reactions with Nickel Boride catalyst (*Top left:* Methionine; *Top right:* Cysteine; *Bottom:* Thioether crosslinked residues). The expected mass loss for each reaction outcome is indicated. See **Methods** for full reaction conditions. (B) Table of different modified species and resulting masses post trypsinolysis and desulfurization. Only calculations and data for the C-terminal CrpA tryptic fragment (ACAGMACAVDCPCAGDVHAGPCIHIPYCPLVL) is shown. (C) *Left:* HR-MS of CrpA post desulfurization with the masses for each modified species indicated. *Right:* Expected isotopic distribution for triply charged unmodified C-terminal tryptic CrpA fragment post desulfurization (*m/z* 980.5422). Differences in observed and calculated relative abundances are shown with red dashed lines. Isotopic distribution was calculated via ChemCalc.[10] See **Figures S23-S24** and **Tables S15-16** for MS/MS spectra and daughter ion assignments.

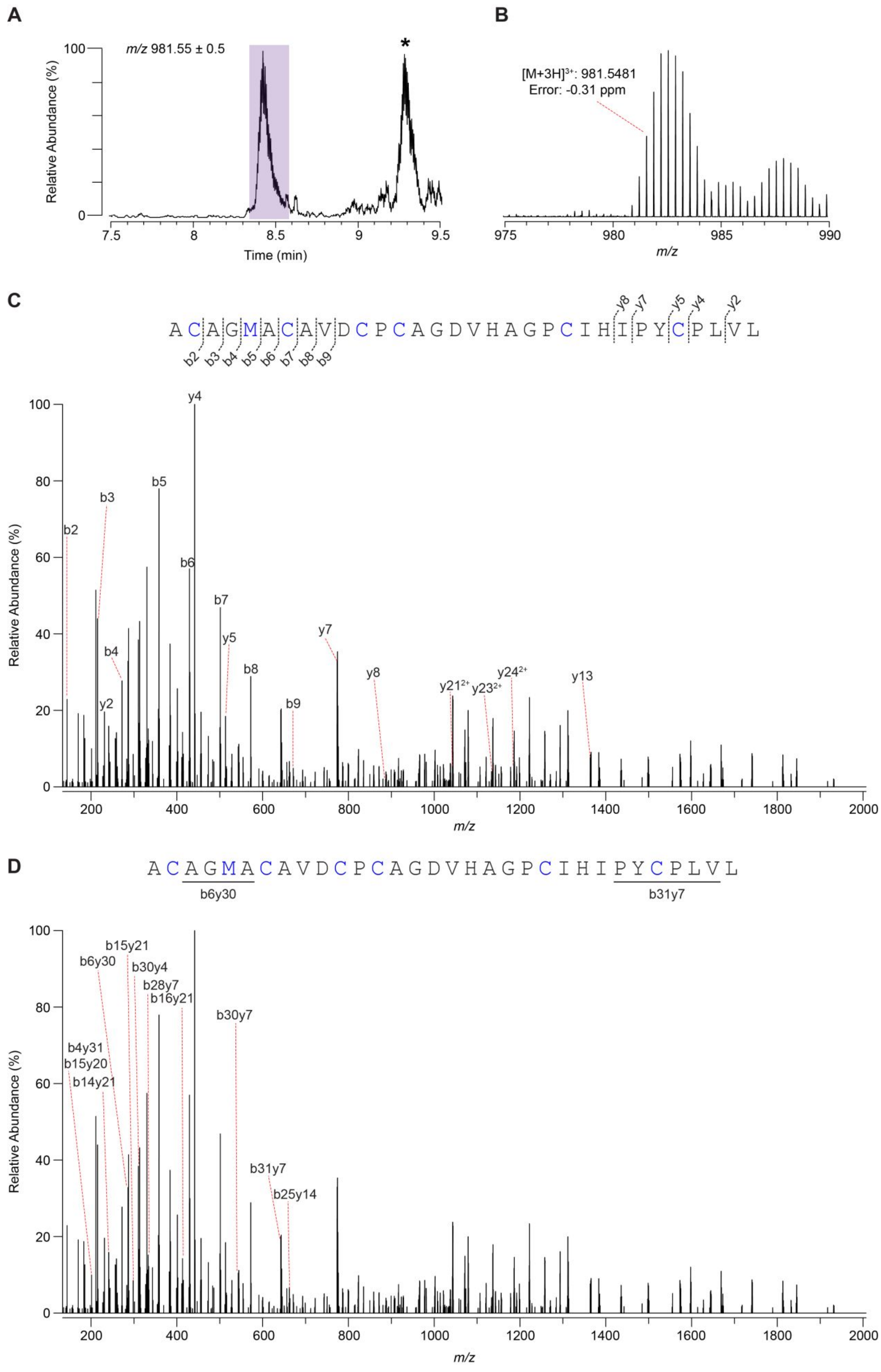
A
B
C
D
m/z 981.55 ± 0.5
Relative Abundance (%)
Time (min)
*
[M+3H]3+: 981.5481
Error: -0.31 ppm
m/z
ACAGMACAVDCPCAGDVHAGPCIHIPYCPLVL
b2 b3 b4 b5 b6 b7 b8 b9
y8 y7 y5 y4 y2
y4 b5 b3 b2 b6 b7 y5 b4 y7 y2 b8 y8 b9 y21 2+ y23 2+ y24 2+ y13
b6y30
b31y7
b15y21 b6y30 b30y4 b28y7 b16y21 b30y7 b4y31 b15y20 b14y21 b31y7 b25y14

Figure S22 (previous page): HR-LC-MS/MS analysis of unmodified and trypsin-digested CrpA post desulfurization. (A) EIC for the C-terminal desulfurized CrpA tryptic fragment and (B) the HR-MS spectrum of the peptide fragment. The selected molecular ion (*m/z* 981.55 ± 0.5; corresponding to the CrpA tryptic fragment with 3 modifications as indicated in **Figure S21)** in the purple shaded area was isolated for MS/MS. * indicates an unrelated species. After desulfurization, the masses for each CrpA peptide with 0-4 modifications were separated by 1 Da each; however, at the 3+ charge state, each modified species overlaps with the ^{13}C isotope of the preceding CrpA peptide (see **Figure S21**). Therefore, there were mixtures of unmodified and modified peptide collected in the same spectrum. (C) *Top:* Summary of observed HCD daughter ions with a focus on unmodified CrpA. Blue cysteine and methionine residues were desulfurized with replacement of a deuterium. *Bottom:* HCD MS/MS spectrum. All ions are shown in the 1+ charge state unless otherwise stated. See **Table S15** for exact masses and errors. The highlighted daughter ions correspond to unmodified fragments (no additional deuterium labeling). An identical MS/MS spectrum with observed daughter ions corresponding to additional deuterium labeling events is shown in **Figure S23**. See **Figure S24** and **Table S17** for comparison and further analysis of key ions. (D) An identical spectrum to (C) with key internal daughter ions indicated. Where masses can correspond to multiple possible internal fragments, the most likely one is shown. See **Table S15** for exact masses and errors of all observed internal ions. The highlighted daughter ions correspond to unmodified fragments (no additional deuterium labeling beyond desulfurization). An identical MS/MS spectrum with observed daughter ions corresponding to additional deuterium labeling events is shown in **Figure S23**. See **Figure S24** and **Table S17** for comparison and further analysis of key ions.

Table S15: Assignment of HCD daughter ions for unmodified and trypsin-digested CrpA post desulfurization. All ions are shown in the 1+ charge state unless otherwise stated. Corresponding HCD spectrum is shown in **Figure S22**. All observed internal ions are included, not just the key ones highlighted in **Figure S22**. Where masses can correspond to multiple possible internal fragments, all are listed.

Ion	Calculated *m/z*	Observed *m/z*	Error (ppm)		Ion	Calculated *m/z*	Observed *m/z*	Error (ppm)
$b2^+$	144.0879	144.088	0.69		$b9^+$	671.3914	671.3909	-0.74
$b3^+$	215.1249	215.1254	2.32		$y2^+$	231.1703	231.1707	1.73
$b4^+$	272.1464	272.1469	1.84		$y4^+$	441.3071	441.3073	0.45
$b5^+$	358.2053	358.2055	0.56		$y5^+$	513.3505	513.3506	0.19
$b6^+$	429.2425	429.2432	1.63		$y7^+$	773.4666	773.4696	3.88
$b7^+$	501.2858	501.2866	1.60		$y8^+$	886.5507	886.5498	-1.02
$b8^+$	572.3229	572.3228	-0.17					

Ion	Calculated *m/z*	Observed *m/z*	Error (ppm)		Ion	Calculated *m/z*	Observed *m/z*	Error (ppm)
b4y31	201.1092	201.1093	0.50		b29y6	333.1668	333.1672	1.20
b15y20	201.1092	201.1093	0.50		b31y5	382.2559	382.2567	2.09
b22y13	227.1249	227.125	0.44		b16y21	413.1889	413.1896	1.69
b14y21	241.1405	241.1407	0.83		b7y31	430.2487	430.2495	1.86
b13y22	242.1468	242.1467	-0.41		b29y8	543.3036	543.304	0.74
b6y30	286.162	286.1625	1.75		b30y7	543.3036	543.304	0.74
b15y21	298.162	298.1622	0.67		b31y7	642.372	642.3726	0.93
b22y14	298.162	298.1622	0.67		b25y14	661.389	661.3904	2.12
b30y4	310.2125	310.2128	0.97		b16y24	699.3277	699.3276	-0.14
b14y22	313.1839	313.1841	0.64		b17y23	699.3277	699.3276	-0.14
b28y7	333.1668	333.1672	1.20					

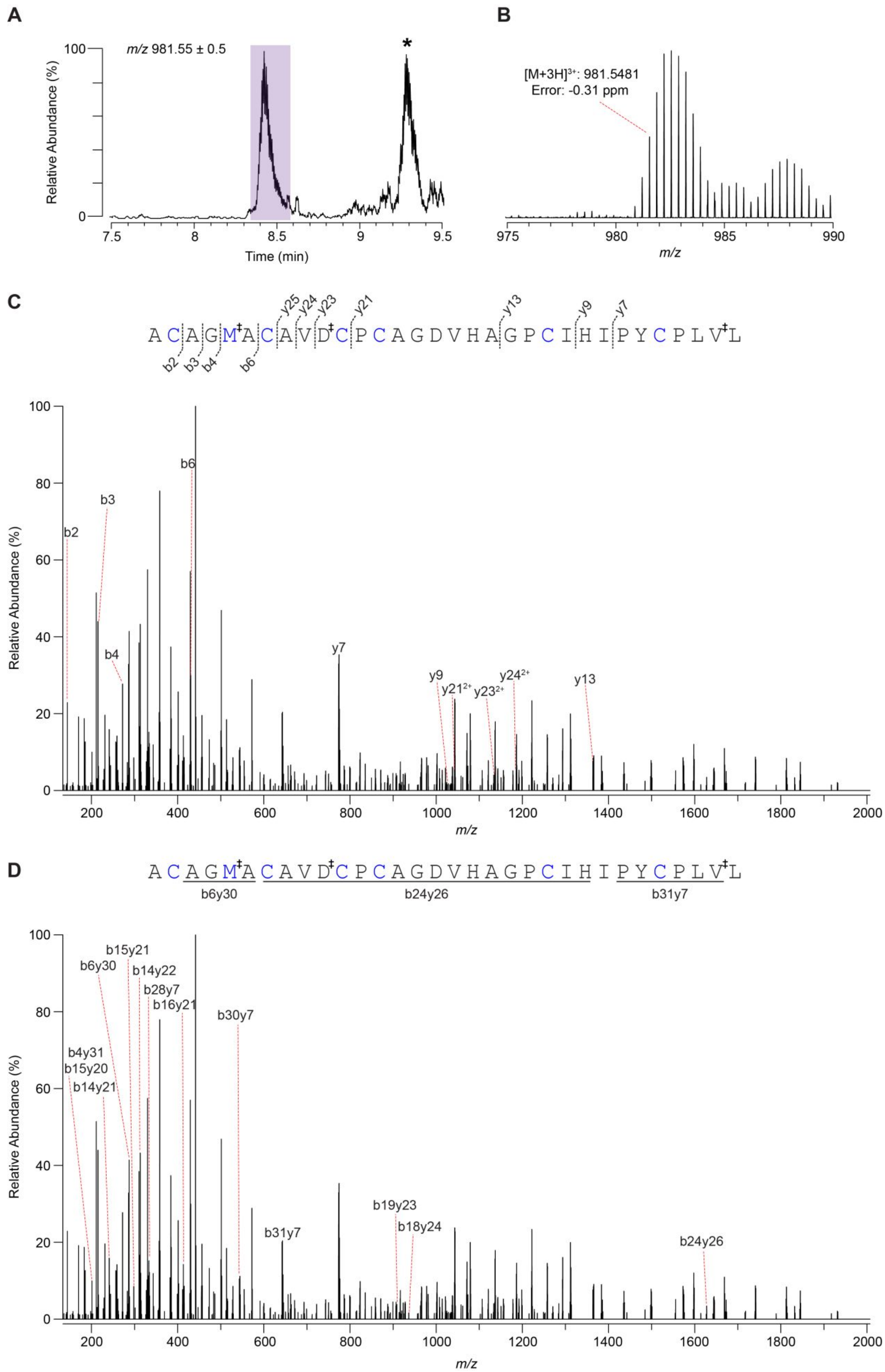

A
m/z 981.55 ± 0.5
Relative Abundance (%)
Time (min)
*
B
[M+3H]3+: 981.5481
Error: -0.31 ppm
m/z
C
A C A G M ‡ A C A V D ‡ C P C A G D V H A G P C I H I P Y C P L V ‡ L
b2 b3 b4 b6
y25 y24 y23 y21 y13 y9 y7
Relative Abundance (%)
b2
b3
b4
b6
y7
y9
y21 2+
y23 2+
y24 2+
y13
m/z
D
A C A G M ‡ A C A V D ‡ C P C A G D V H A G P C I H I P Y C P L V ‡ L
b6y30
b24y26
b31y7
Relative Abundance (%)
b15y21
b6y30
b14y22
b28y7
b16y21
b30y7
b4y31
b15y20
b14y21
b31y7
b19y23
b18y24
b24y26
m/z

Figure S23 (previous page): HR-LC-MS/MS analysis of modified and trypsin-digested CrpA post desulfurization. (A) EIC for the C-terminal desulfurized CrpA tryptic fragment and (B) the HR-MS spectrum of the peptide fragment. The selected molecular ion (*m/z* 981.55 ± 0.5; corresponding to the CrpA tryptic fragment with 3 modifications as indicated in Figure S19) in the purple shaded area was fragmented via higher-energy collision induced dissociation (HCD). * indicates an unrelated species. After desulfurization, the masses for each CrpA peptide with 0-4 modifications were separated by 1 Da each; however, at the 3+ charge state, each modified species overlaps with the ^{13}C isotope of the preceding CrpA peptide (see **Figure S21**). Therefore, there were mixtures of unmodified and modified peptide collected in the same spectrum. (C) *Top:* Summary of observed HCD daughter ions. Blue cysteine and methionine residues were desulfurized with replacement of a deuterium atom. Residues marked with ‡ harbor an additional deuterium atom. *Bottom:* HCD MS/MS spectrum. All ions are shown in the 1+ charge state unless otherwise stated. See **Table S16** for exact masses and errors. While multiple daughter ions present in the spectrum correspond to unmodified, but desulfurized, CrpA, there is evidence for additional deuterium labeling based on expected and observed isotopic ratios. See **Figure S24** and **Table S17** for further analysis of these key ions. (D) An identical spectrum to (C) with key internal daughter ions indicated. Where masses can correspond to multiple possible internal fragments, the most likely one is shown. See **Table S16** for exact masses and errors of all observed internal ions. While multiple daughter ions present in the spectrum correspond to unmodified, but desulfurized, CrpA, there is evidence for additional deuterium labeling based on expected and observed isotopic ratios. See **Figure S24** and **Table S17** for further analysis of these key ions.

Table S16: Assignment of HCD daughter ions for modified and trypsin-digested CrpA post desulfurization. All ions are shown in the 1+ charge state unless otherwise stated. Corresponding HCD spectrum is shown in **Figure S23**. All observed internal ions are included, not just the key ones highlighted in **Figure S23**. Multiple possible internal fragments are listed if they have the same *m/z*.

Ion	Calculated *m/z*	Observed *m/z*	Error (ppm)		Ion	Calculated *m/z*	Observed *m/z*	Error (ppm)
$[M+3H]^{3+}$	981.5484	981.5481	-0.31		y9	1024.6159	1024.6165	0.59
b2	144.0878	144.088	1.39		y13	1363.8176	1363.8161	-1.10
b3	215.1249	215.1254	2.32		$y21^{2+}$	1042.0855	1042.0856	0.10
b4	272.1464	272.1469	1.84		$y23^{2+}$	1136.1238	1136.1213	-2.20
b6	430.2488	430.2495	1.63		$y24^{2+}$	1185.658	1185.658	0.00
y7	774.4729	774.4739	1.29		$y25^{2+}$	1221.1766	1221.1733	-2.70

Ion	Calculated *m/z*	Observed *m/z*	Error (ppm)		Ion	Calculated *m/z*	Observed *m/z*	Error (ppm)
b4y31	201.1092	201.1093	0.50		b16y21	413.1889	413.1896	1.69
b15y20	201.1092	201.1093	0.50		b29y8	543.3036	543.304	0.74
b14y21	241.1405	241.1407	0.83		b30y7	543.3036	543.304	0.74
b6y30	287.1683	287.1684	0.35		b31y7	643.3783	643.3798	2.33
b15y21	298.162	298.1622	0.67		b18y22	721.3597	721.3587	-1.39
b22y14	298.162	298.1622	0.67		b19y23	908.43	908.4282	-1.98
b14y22	313.1839	313.1841	0.64		b18y24	936.4613	936.4616	0.32
b28y7	333.1668	333.1672	1.20		b24y26	1626.8395	1626.841	0.92
b29y6	333.1668	333.1672	1.20					

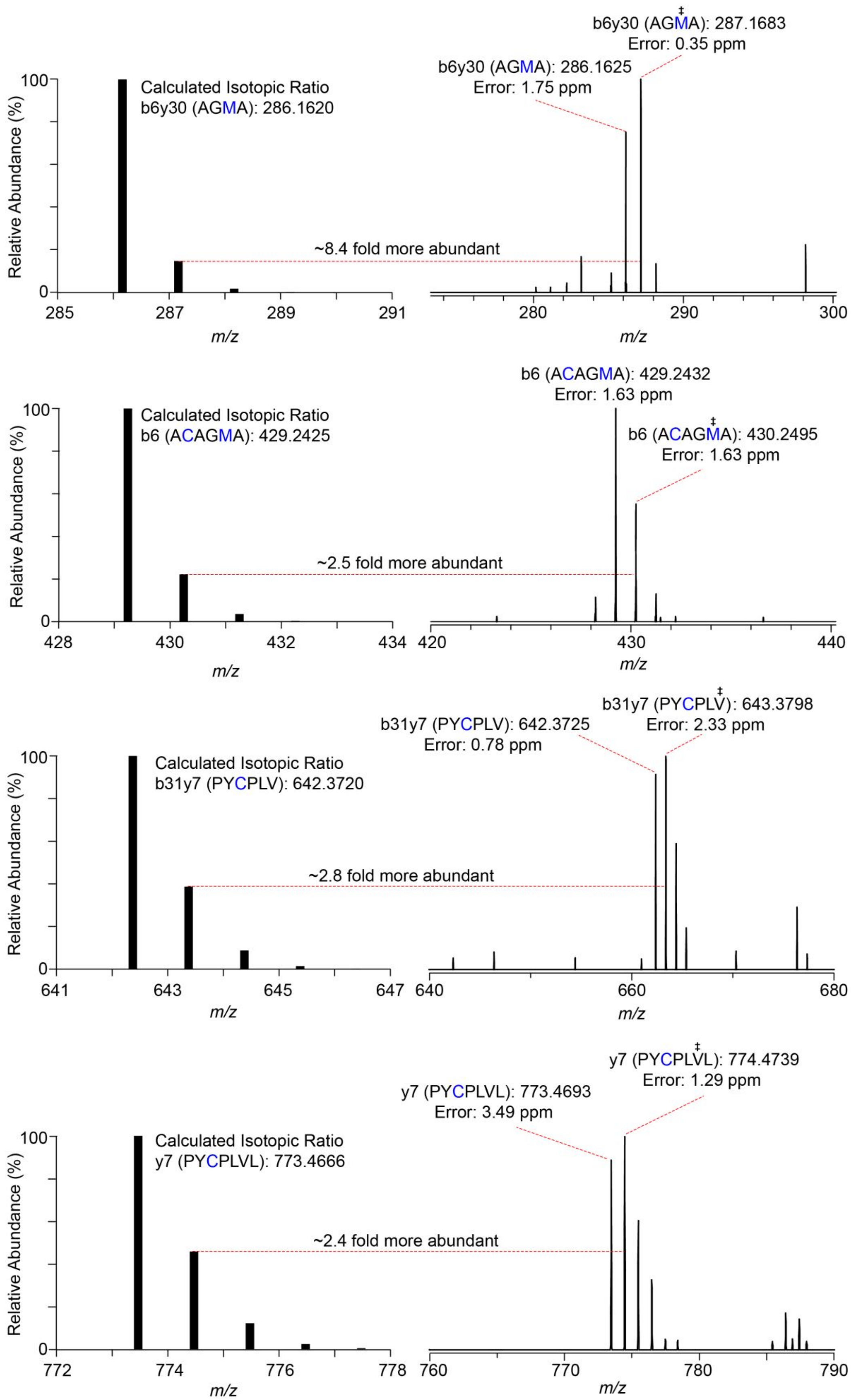

b6y30 (AGM‡A): 287.1683
Error: 0.35 ppm
b6y30 (AGMA): 286.1625
Error: 1.75 ppm
Calculated Isotopic Ratio
b6y30 (AGMA): 286.1620
~8.4 fold more abundant
Relative Abundance (%)
m/z
b6 (ACAGMA): 429.2432
Error: 1.63 ppm
Calculated Isotopic Ratio
b6 (ACAGMA): 429.2425
b6 (ACAGM‡A): 430.2495
Error: 1.63 ppm
~2.5 fold more abundant
b31y7 (PYCPLV‡): 643.3798
Error: 2.33 ppm
b31y7 (PYCPLV): 642.3725
Error: 0.78 ppm
Calculated Isotopic Ratio
b31y7 (PYCPLV): 642.3720
~2.8 fold more abundant
y7 (PYCPLV‡L): 774.4739
Error: 1.29 ppm
y7 (PYCPLVL): 773.4693
Error: 3.49 ppm
Calculated Isotopic Ratio
y7 (PYCPLVL): 773.4666
~2.4 fold more abundant

Figure S24 (previous page): Analysis of isotopic ratios for assignment of selected HCD daughter ions for modified and trypsin-digested CrpA post desulfurization. See methods for full experimental details. Four key daughter ions demonstrating the incorporation of additional deuterium atoms on non-sulfur containing residues. See **Figures S22-S23** for the full HCD spectrum. *Left:* The expected isotopic ratio for unmodified CrpA (no additional deuterium atoms added beyond sulfur replacement). *Right:* Observed MS/MS data for each selected ion. The unmodified CrpA (no additional deuterium label) and CrpA fragment with additional deuterium labeling. Cysteine and methionine residues colored blue were successfully desulfurized with concurrent replacement with deuterium, while residues marked with ‡ indicate an additional 1 Da mass increase (deuterium incorporation). The fold change in relative abundance is indicated. See **Table S17** for a comparison of expected and observed m/z values for ^{13}C and deuterium labeling.

Table S17: Comparison ^{13}C isotope and deuterium labeling *m/z* errors for selected daughter ions for modified and trypsin-digested CrpA post desulfurization. See **Figure S24** for spectral and isotopic ratio comparisons of the same four daughter ions of interest and **Figures S22-S23** for the full HCD spectra. For each ion, the top row is the unmodified *m/z*, the middle row is the calculated ^{13}C *m/z* from the unmodified peptide, and the bottom row is the calculated *m/z* for the fragment with an additional deuterium incorporated.

Ion	Molecular Formula	Calculated *m/z*	Observed *m/z*	Error (ppm)
b6y30	$C_{12}H_{20}DN_4O_4^+$	286.1620	286.1625	1.75
	$C_{11}{}^{13}CH_{20}DN4O_4^+$	287.1654	287.1684	10.45
	$C_{12}H_{19}D_2N_4O_4^+$	287.1683	287.1684	0.35
b6	$C_{18}H_{29}D_2N_6O_6^+$	429.2425	429.2433	1.86
	$C_{17}{}^{13}CH_{29}D_2N_6O_6^+$	430.2459	430.2495	8.37
	$C_{18}H_{28}D_3N_6O_6^+$	430.2488	430.2495	1.63
b31y7	$C_{33}H_{48}DN_6O_7^+$	642.3720	642.3726	0.93
	$C_{32}{}^{13}CH_{48}DN_6O_7^+$	643.3754	643.3798	6.84
	$C_{33}H_{47}D_2N_6O_7^+$	643.3783	643.3798	2.33
y7	$C_{39}H_{61}DN_7O_9^+$	773.4666	773.4693	3.49
	$C_{38}{}^{13}CH_{61}DN_7O_9^+$	774.4700	774.4733	4.26
	$C_{39}H_{60}D_2N_7O_9^+$	774.4729	774.4733	0.52

A

% Similarity (lower left) / % Identity (upper right)

Group		CrpC	AlbA	ThnB	StsB	SkfB	GggB	RumMc1	RumMc2	TrnC	KgrC	QmpB	CteB	PapB	NxxcB	QhpD	SuiB	AnSME
	CrpC	100	0	0	0	0	33	0	0	0	0	33	33	17	33	17	17	17
Sactisynthases	AlbA	50	100	50	67	67	33	17	17	17	50	17	17	33	17	33	17	17
Sactisynthases	ThnB	33	50	100	33	33	17	0	0	0	33	33	0	17	33	17	0	0
Sactisynthases	StsB	50	83	50	100	67	33	17	17	17	50	17	17	33	17	33	17	17
Sactisynthases	SkfB	33	67	33	67	100	33	17	17	17	67	17	17	33	17	33	17	17
Sactisynthases	GggB	83	50	33	50	33	100	17	17	17	33	33	33	67	33	67	17	17
Sactisynthases	RumMc1	33	17	0	17	17	50	100	100	83	17	0	17	17	0	17	17	17
Sactisynthases	RumMc2	33	17	0	17	17	50	100	100	83	17	0	17	17	0	17	17	17
Sactisynthases	TrnC	33	17	0	17	17	50	100	100	100	17	17	33	33	17	33	33	17
Sactisynthases	KgrC	33	50	33	50	67	33	17	17	17	100	17	17	33	17	33	17	17
Sactisynthases	QmpB	50	50	33	50	50	50	50	50	50	33	100	33	50	100	50	17	0
Ranthisynthases	CteB	50	33	17	50	33	50	50	50	50	33	67	100	67	33	67	50	33
Ranthisynthases	PapB	50	33	17	50	33	67	67	67	67	33	67	83	100	50	100	33	17
Ranthisynthases	NxxcB	50	50	33	50	50	50	50	50	50	33	100	67	67	100	50	17	0
Ranthisynthases	QhpD	50	33	17	50	33	67	67	67	67	33	67	83	100	67	100	33	17
Other	SuiB	33	33	17	33	33	33	33	33	33	33	50	50	50	50	50	100	33
Other	AnSME	33	33	17	33	33	33	33	33	33	33	33	33	33	33	33	33	100

B

Group		CrpC	AlbA	ThnB	StsB	SkfB	GggB	RumMc1	RumMc2	TrnC	KgrC	QmpB	CteB	PapB	NxxcB	QhpD	SuiB	AnSME
	CrpC	1	-0.1	-0.09	-0.03	-0.12	0.46	0	0	0.06	-0.11	0.31	0.38	0.22	0.31	0.22	0.11	-0.15
Sactisynthases	AlbA	-0.08	1	0.58	0.72	0.61	0.18	-0.13	-0.13	-0.1	0.41	0.18	0	0.11	0.18	0.11	0.11	0.06
Sactisynthases	ThnB	-0.08	0.64	1	0.51	0.41	0.09	-0.24	-0.24	-0.2	0.29	0.28	-0.05	0.02	0.28	0.02	-0.03	-0.03
Sactisynthases	StsB	-0.02	0.75	0.48	1	0.67	0.21	-0.06	-0.06	-0.03	0.41	0.15	0.02	0.14	0.15	0.14	0.03	0.06
Sactisynthases	SkfB	-0.1	0.67	0.41	0.72	1	0.09	-0.03	-0.03	-0.03	0.58	0.12	0.02	0.14	0.12	0.14	0	0.09
Sactisynthases	GggB	0.4	0.21	0.09	0.24	0.09	1	0.1	0.1	0.16	0.08	0.28	0.38	0.6	0.28	0.6	0.22	-0.12
Sactisynthases	RumMc1	0	-0.14	-0.22	-0.06	-0.03	0.09	1	1	0.86	-0.08	0.03	0.26	0.25	0.03	0.25	0	0.06
Sactisynthases	RumMc2	0	-0.14	-0.22	-0.06	-0.03	0.09	1	1	0.86	-0.08	0.03	0.26	0.25	0.03	0.25	0	0.06
Sactisynthases	TrnC	0.05	-0.1	-0.19	-0.03	-0.03	0.15	0.89	0.89	1	-0.08	0.15	0.38	0.37	0.15	0.37	0.14	0.03
Sactisynthases	KgrC	-0.1	0.5	0.32	0.48	0.64	0.09	-0.1	-0.1	-0.1	1	0.09	0	0.11	0.09	0.11	0.03	0
Sactisynthases	QmpB	0.27	0.21	0.29	0.17	0.12	0.28	0.03	0.03	0.16	0.08	1	0.32	0.4	1	0.4	0.29	-0.06
Ranthisynthases	CteB	0.35	0	-0.06	0.03	0.03	0.4	0.31	0.31	0.43	0	0.34	1	0.77	0.34	0.77	0.4	0.06
Ranthisynthases	PapB	0.21	0.14	0.03	0.17	0.16	0.65	0.31	0.31	0.43	0.11	0.43	0.79	1	0.43	1	0.29	-0.03
Ranthisynthases	NxxcB	0.27	0.21	0.29	0.17	0.12	0.28	0.03	0.03	0.16	0.08	1	0.32	0.4	1	0.4	0.29	-0.06
Ranthisynthases	QhpD	0.21	0.14	0.03	0.17	0.16	0.65	0.31	0.31	0.43	0.11	0.43	0.79	1	0.43	1	0.29	-0.03
Other	SuiB	0.08	0.1	-0.03	0.03	0	0.18	0	0	0.13	0.02	0.25	0.32	0.22	0.25	0.22	1	0.06
Other	AnSME	-0.13	0.07	-0.03	0.06	0.09	-0.12	0.06	0.06	0.03	0	-0.06	0.05	-0.02	-0.06	-0.02	0.07	1

Figure S25: Catalytic proximity profiling of CrpC. (A) Residue similarity and identity matrix and (B) Global similarity matrix of the catalytic proximity profiling residues for CrpC compared to sactisyntheases, ranthisyntheases, and other RS enzymes. Matrix was generated via Sequence Similarity and Identity (SIAS; http://imed.med.ucm.es/Tools/sias.html) using BLOSUM62 and default settings.

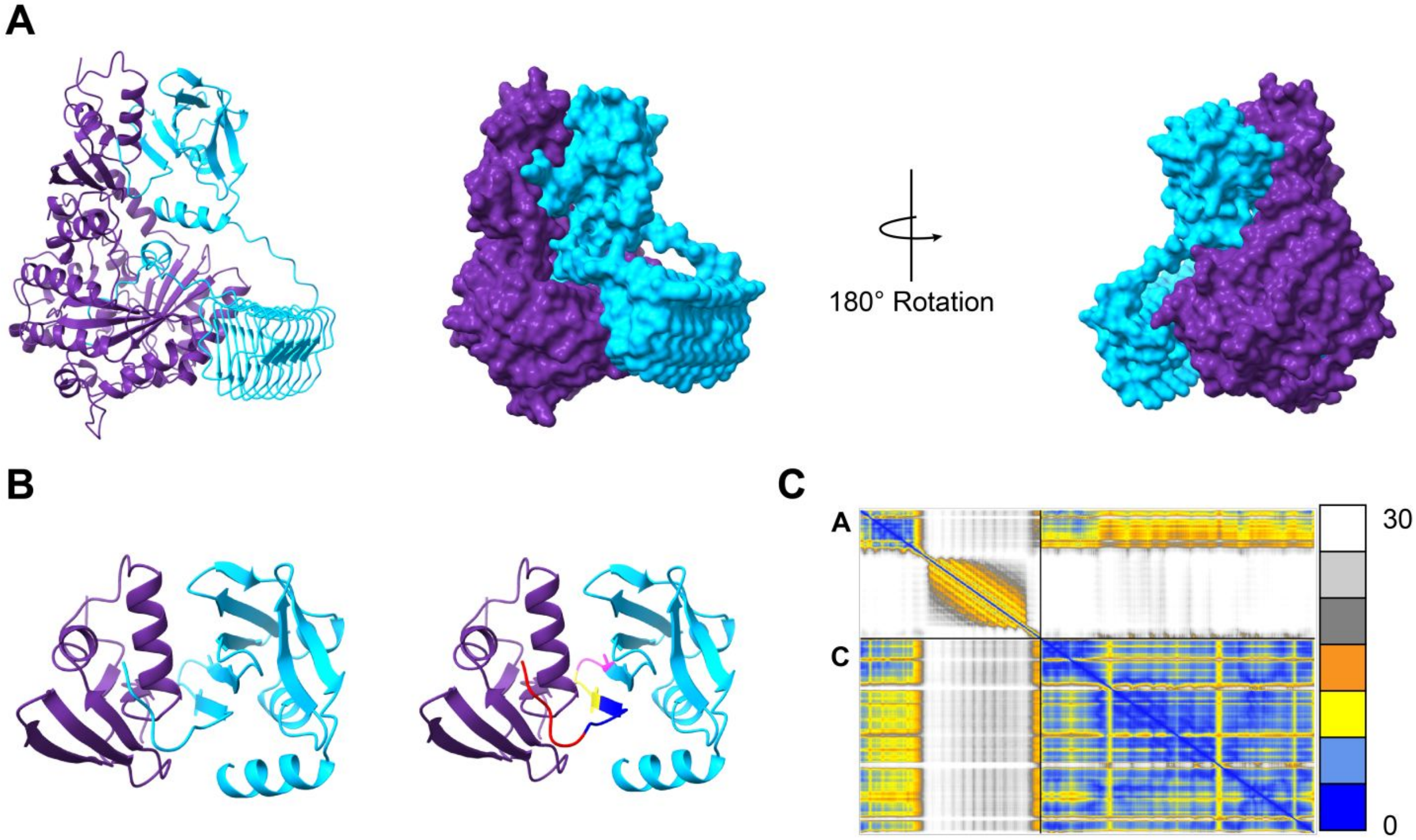

Figure S26: AlphaFold3 multimer structures of CrpAC. AlphaFold3[13] multimer predicted structures and of CrpA (light blue) and CrpC (purple). The multimer structure is shown in both cartoon and surface. (B) AlphaFold3 multimer structures with a focus on the predicted CrpC RRE (purple) and CrpA N-terminal domain interaction. The right structure is colored according to N-terminal truncations (red = 5ΔN, dark blue = 10ΔN, yellow = 15ΔN, and pink = 20ΔN). For CrpA 86ΔN, the entire region shown was removed. For CrpC ΔRRE, the shown RRE domain was removed from CrpC. (C) PAE plots for the CrpAC multimer model. The PAE scale increases from low error (blue) to high error (white). See **Figure S5** for the individual PAE and pLDDT plots for CrpA.

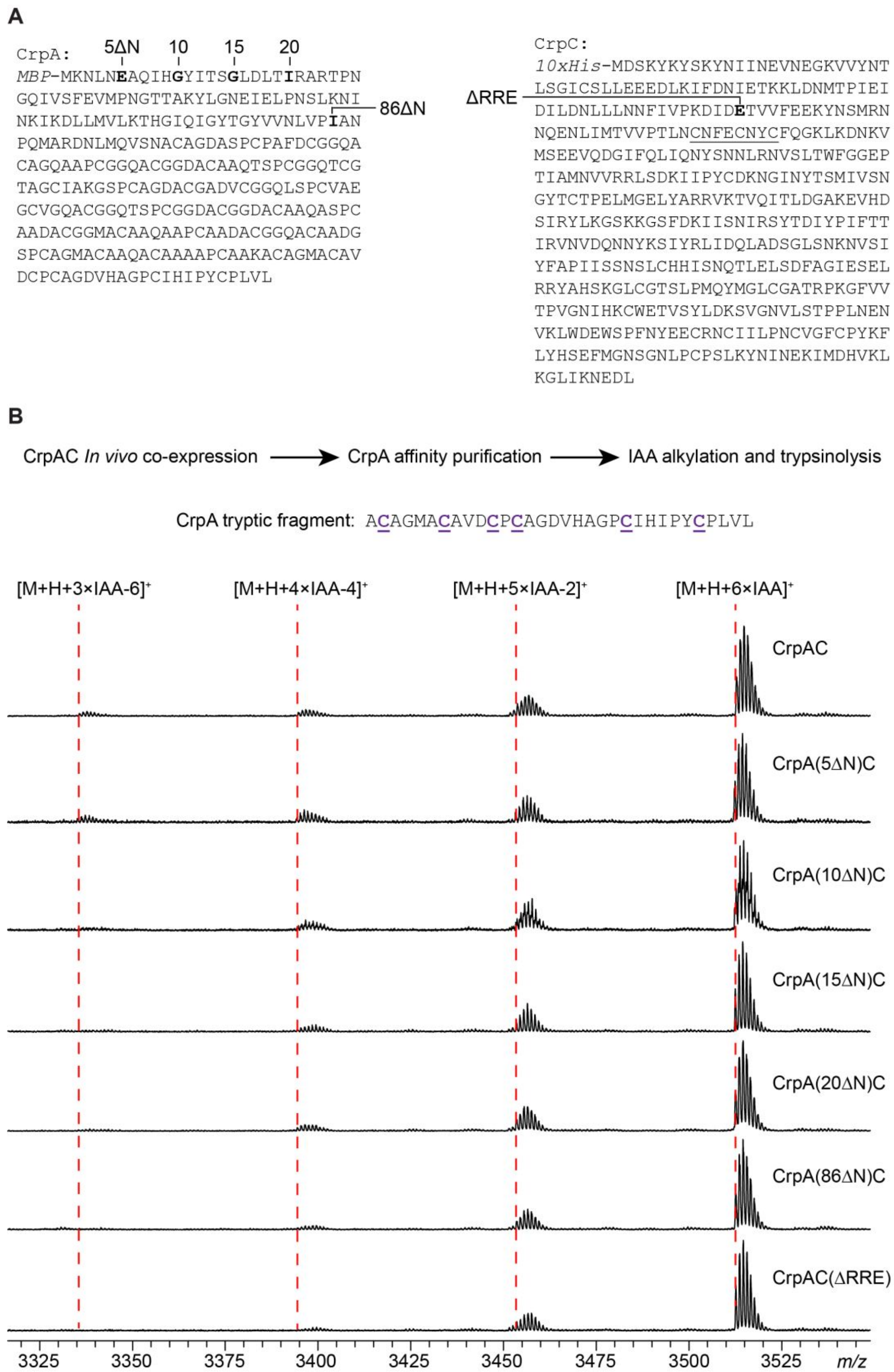

Figure S27: Mass spectrometry analysis of CrpAC N-terminal truncations. (A) Sequences of CrpA and CrpC with truncation positions indicated by numbering and bolded residues. The active site RS [4Fe-4S] binding motif in CrpC is underlined. (B) *Top:* Workflow and tryptic fragment of interest are shown. Cysteine residues are colored purple and underlined. *Bottom:* MALDI-TOF-MS spectra of IAA alkylated and trypsin digested CrpAC N-terminal truncations. Masses for the different modified species are labeled.

References

(1) Repka, L. M.; Chekan, J. R.; Nair, S. K.; van der Donk, W. A. Mechanistic Understanding of Lanthipeptide Biosynthetic Enzymes. *Chem. Rev.* **2017**, *117* (8), 5457–5520. https://doi.org/10.1021/acs.chemrev.6b00591

(2) Lu, J.; Li, Y.; Bai, Z.; Lv, H.; Wang, H. Enzymatic Macrocyclization of Ribosomally Synthesized and Posttranslational Modified Peptides via C–S and C–C Bond Formation. *Nat. Prod. Rep.* **2021**, *38* (5), 981–992. https://doi.org/10.1039/D0NP00044B

(3) Montalbán-López, M.; Scott, T. A.; Ramesh, S.; Rahman, I. R.; Heel, A. J. van; Viel, J. H.; Bandarian, V.; Dittmann, E.; Genilloud, O.; Goto, Y.; Burgos, M. J. G.; Hill, C.; Kim, S.; Koehnke, J.; Latham, J. A.; Link, A. J.; Martínez, B.; Nair, S. K.; Nicolet, Y.; Rebuffat, S.; Sahl, H.-G.; Sareen, D.; Schmidt, E. W.; Schmitt, L.; Severinov, K.; Süssmuth, R. D.; Truman, A. W.; Wang, H.; Weng, J.-K.; Wezel, G. P. van; Zhang, Q.; Zhong, J.; Piel, J.; Mitchell, D. A.; Kuipers, O. P.; Donk, W. A. van der. New Developments in RiPP Discovery, Enzymology and Engineering. *Nat. Prod. Rep.* **2021**, *38* (1), 130–239. https://doi.org/10.1039/D0NP00027B

(4) Datta, S.; Mori, Y.; Takagi, K.; Kawaguchi, K.; Chen, Z.-W.; Okajima, T.; Kuroda, S.; Ikeda, T.; Kano, K.; Tanizawa, K.; Mathews, F. S. Structure of a Quinohemoprotein Amine Dehydrogenase with an Uncommon Redox Cofactor and Highly Unusual Crosslinking. *Proc. Natl. Acad. Sci.* **2001**, *98* (25), 14268–14273. https://doi.org/10.1073/pnas.241429098

(5) Nakai, T.; Ito, H.; Kobayashi, K.; Takahashi, Y.; Hori, H.; Tsubaki, M.; Tanizawa, K.; Okajima, T. The Radical *S*-Adenosyl-l-Methionine Enzyme QhpD Catalyzes Sequential Formation of Intra-Protein Sulfur-to-Methylene Carbon Thioether Bonds*. *J. Biol. Chem.* **2015**, *290* (17), 11144–11166. https://doi.org/10.1074/jbc.M115.638320

(6) Tietz, J. I.; Schwalen, C. J.; Patel, P. S.; Maxson, T.; Blair, P. M.; Tai, H.-C.; Zakai, U. I.; Mitchell, D. A. A New Genome-Mining Tool Redefines the Lasso Peptide Biosynthetic Landscape. *Nat. Chem. Biol.* **2017**, *13* (5), 470–478. https://doi.org/10.1038/nchembio.2319

(7) Crooks, G. E.; Hon, G.; Chandonia, J.-M.; Brenner, S. E. WebLogo: A Sequence Logo Generator. *Genome Res.* **2004**, *14* (6), 1188–1190. https://doi.org/10.1101/gr.849004

(8) Edgar, R. C. Muscle5: High-Accuracy Alignment Ensembles Enable Unbiased Assessments of Sequence Homology and Phylogeny. *Nat. Commun.* **2022**, *13* (1), 6968. https://doi.org/10.1038/s41467-022-34630-w

(9) Beinert, H. Semi-Micro Methods for Analysis of Labile Sulfide and of Labile Sulfide plus Sulfane Sulfur in Unusually Stable Iron-Sulfur Proteins. *Anal. Biochem.* **1983**, *131* (2), 373–378. https://doi.org/10.1016/0003-2697(83)90186-0

(10) Patiny, L.; Borel, A. ChemCalc: A Building Block for Tomorrow's Chemical Infrastructure. *J. Chem. Inf. Model.* **2013**, *53* (5), 1223–1228. https://doi.org/10.1021/ci300563h

(11) Liang, Y.; Neta, P.; Yang, X.; Stein, S. E. Collision-Induced Dissociation of Deprotonated Peptides. Relative Abundance of Side-Chain Neutral Losses, Residue-Specific Product Ions, and Comparison with Protonated Peptides. *J. Am. Soc. Mass Spectrom.* **2018**, *29* (3), 463–469. https://doi.org/10.1007/s13361-017-1842-5

(12) Simón-Manso, Y.; Neta, P.; Yang, X.; Stein, S. E. Loss of 45 Da from A2 Ions and Preferential Loss of 48 Da from A2 Ions Containing Methionine in Peptide Ion Tandem Mass Spectra. *J. Am. Soc. Mass Spectrom.* **2011**, *22* (2), 280–289. https://doi.org/10.1007/s13361-010-0025-4

(13) Abramson, J.; Adler, J.; Dunger, J.; Evans, R.; Green, T.; Pritzel, A.; Ronneberger, O.; Willmore, L.; Ballard, A. J.; Bambrick, J.; Bodenstein, S. W.; Evans, D. A.; Hung, C.-C.; O'Neill, M.; Reiman, D.; Tunyasuvunakool, K.; Wu, Z.; Žemgulytė, A.; Arvaniti, E.; Beattie, C.; Bertolli, O.; Bridgland, A.; Cherepanov, A.; Congreve, M.; Cowen-Rivers, A. I.; Cowie, A.; Figurnov, M.; Fuchs, F. B.; Gladman, H.; Jain, R.; Khan, Y. A.; Low, C. M. R.; Perlin, K.; Potapenko, A.; Savy, P.; Singh, S.; Stecula, A.; Thillaisundaram, A.; Tong, C.; Yakneen, S.; Zhong, E. D.; Zielinski, M.; Žídek, A.; Bapst, V.; Kohli, P.; Jaderberg, M.; Hassabis, D.; Jumper, J. M. Accurate Structure

Prediction of Biomolecular Interactions with AlphaFold 3. *Nature* **2024**, *630* (8016), 493–500. https://doi.org/10.1038/s41586-024-07487-w
